# Resequencing of 388 cassava accessions identifies valuable loci and selection for variation in heterozygosity

**DOI:** 10.1186/s13059-021-02524-7

**Published:** 2021-11-16

**Authors:** Wei Hu, Changmian Ji, Zhe Liang, Jianqiu Ye, Wenjun Ou, Zehong Ding, Gang Zhou, Weiwei Tie, Yan Yan, Jinghao Yang, Liming Ma, Xiaoying Yang, Yunxie Wei, Zhiqiang Jin, Jianghui Xie, Ming Peng, Wenquan Wang, Anping Guo, Biyu Xu, Jianchun Guo, Songbi Chen, Mingcheng Wang, Yang Zhou, Xiaolong Li, Ruoxi Li, Xinhui Xiao, Zhongqing Wan, Feifei An, Jie Zhang, Qingyun Leng, Yin Li, Haitao Shi, Ray Ming, Kaimian Li

**Affiliations:** 1 Sanya Research Institute of Chinese Academy of Tropical Agricultural Sciences, Sanya, Hainan China; 2 Hainan Key Laboratory for Biosafety Monitoring and Molecular Breeding in Off-Season Reproduction Regions, Key Laboratory of Biology and Genetic Resources of Tropical Crops, Institute of Tropical Bioscience and Biotechnology, Chinese Academy of Tropical Agricultural Sciences, Haikou, Hainan China; 3 Hainan Key Laboratory for Protection and Utilization of Tropical Bioresources, Hainan Institute for Tropical Agricultural Resources, Chinese Academy of Tropical Agricultural Sciences, Haikou, Hainan China; 4 Biotechnology Research Institute, Chinese Academy of Agricultural Sciences, Beijing, China; 5 Tropical Crops Genetic Resources Institute, Chinese Academy of Tropical Agricultural Sciences, Haikou, Hainan China; 6 Biomarker Technologies Corporation, Beijing, China; 7 College of Food Science and Technology, Qilu University of Technology (Shandong Academy of Sciences), Jinan, China; 8 Hainan Key Laboratory for Sustainable Utilization of Tropical Bioresources, College of Tropical Crops, Hainan University, Haikou, Hainan China; 9 Fu Foundation School of Engineering and Applied Science, Columbia University, New York, NY 10027 USA; 10 Waksman Institute of Microbiology, Rutgers, The State University of New Jersey, Piscataway, NJ USA; 11 FAFU and UIUC-SIB Joint Center for Genomics and Biotechnology, Fujian Provincial Key Laboratory of Haixia Applied Plant Systems Biology, Key Laboratory of Genetics, Breeding and Multiple Utilization of Crops, Ministry of Education, Fujian Agriculture and Forestry University, Fuzhou, China; 12 Department of Plant Biology, University of Illinois at Urbana-Champaign, Urbana, IL USA

**Keywords:** Resequencing, Cassava, Heterozygosity, Selection, Agronomic traits

## Abstract

**Background:**

Heterozygous genomes are widespread in outcrossing and clonally propagated crops. However, the variation in heterozygosity underlying key agronomic traits and crop domestication remains largely unknown. Cassava is a staple crop in Africa and other tropical regions and has a highly heterozygous genome.

**Results:**

We describe a genomic variation map from 388 resequenced genomes of cassava cultivars and wild accessions. We identify 52 loci for 23 agronomic traits through a genome-wide association study. Eighteen allelic variations in heterozygosity for nine candidate genes are significantly associated with seven key agronomic traits. We detect 81 selective sweeps with decreasing heterozygosity and nucleotide diversity, harboring 548 genes, which are enriched in multiple biological processes including growth, development, hormone metabolisms and responses, and immune-related processes. Artificial selection for decreased heterozygosity has contributed to the domestication of the large starchy storage root of cassava. Selection for homozygous GG allele in *MeTIR1* during domestication contributes to increased starch content. Selection of homozygous AA allele in *MeAHL17* is associated with increased storage root weight and cassava bacterial blight (CBB) susceptibility. We have verified the positive roles of *MeTIR1* in increasing starch content and *MeAHL17* in resistance to CBB by transient overexpression and silencing analysis. The allelic combinations in *MeTIR1* and *MeAHL17* may result in high starch content and resistance to CBB.

**Conclusions:**

This study provides insights into allelic variation in heterozygosity associated with key agronomic traits and cassava domestication. It also offers valuable resources for the improvement of cassava and other highly heterozygous crops.

**Supplementary Information:**

The online version contains supplementary material available at 10.1186/s13059-021-02524-7.

## Background

The capacity and efficiency of plant breeding contribute greatly to global food production and human life [1]. The development and application of genomic technologies offer powerful tools for precise selection and directional breeding [2]. Associations between genotypes and phenotypes in populations have revealed homozygous allelic variations that are significantly associated with key agronomic traits in many crops, including rice [3], maize [4], tomato [2], and cotton [5], thereby accelerating the breeding process. However, many crops are highly heterozygous due to outcrossing or clonal propagation, such as cassava [6], rubber tree [7], and mango [8]. Currently, the effect of variation in heterozygosity on agronomic traits is still less known.

Throughout the history of crop domestication, numerous wild species were domesticated into cultivated crops by artificial selection. Subsequently, along with human migration, directional selection, and further trait improvement to meet human demands, cultivated crops acquired region-specific differences in traits [2]. Using genome sequencing technologies, we can track crop domestication and breeding history, and thereby better understand how human selection shaped crop genomes, as shown in tomato [2], cotton [9], rice [10], maize [11], soybean [12], peach [13], melon [14], watermelon [15], and pineapple [16]. However, the variation in heterozygosity underlying crop domestication remains largely unknown.

Cassava (*Manihot esculenta* Crantz) belongs to the Euphorbiaceae family, and represents one of the most widely cultivated crops in tropical areas [6, 17]. Annual production of cassava is 276.7 million tons, and it provides staple food for nearly one billion people in 105 countries in tropical regions across Africa, South America, and Asia [18, 19]. Cultivated cassava was domesticated from its wild progenitor, *Manihot esculenta* ssp. *flabellifolia*, in the Amazon basin over 6000 years ago [6, 20]. Domestication of cassava has resulted in characteristics including an annualized growth cycle, high initial growth rate, increased yield, and high starch content [20–24], which makes cassava a desirable energy source both for human consumption and industrial biofuel applications. Although cassava contributes greatly to food security in tropical areas and developing countries, research on cassava genomics and breeding is relatively lagging behind due to the limited genomic resources [6, 20, 24, 25]. The development of population genomic resources will accelerate the progress of genetic improvement for this important crop.

The cassava genome (2n = 36) has a relatively high heterozygosity of 0.61–0.84% among cultivars and wild progenitors, which makes it an excellent system for studying variation in heterozygosity [6, 20, 26]. In the present study, we performed large-scale resequencing of cassava genomes, population genomic analyses, and genome-wide association studies (GWAS) for key agronomic traits. We identified valuable loci with variation in heterozygosity associated with key agronomic traits and our results offered insights into variation in heterozygosity during cassava domestication.

## Results

### Genome variation map and phylogenetic relationship

**Fig. 1:**
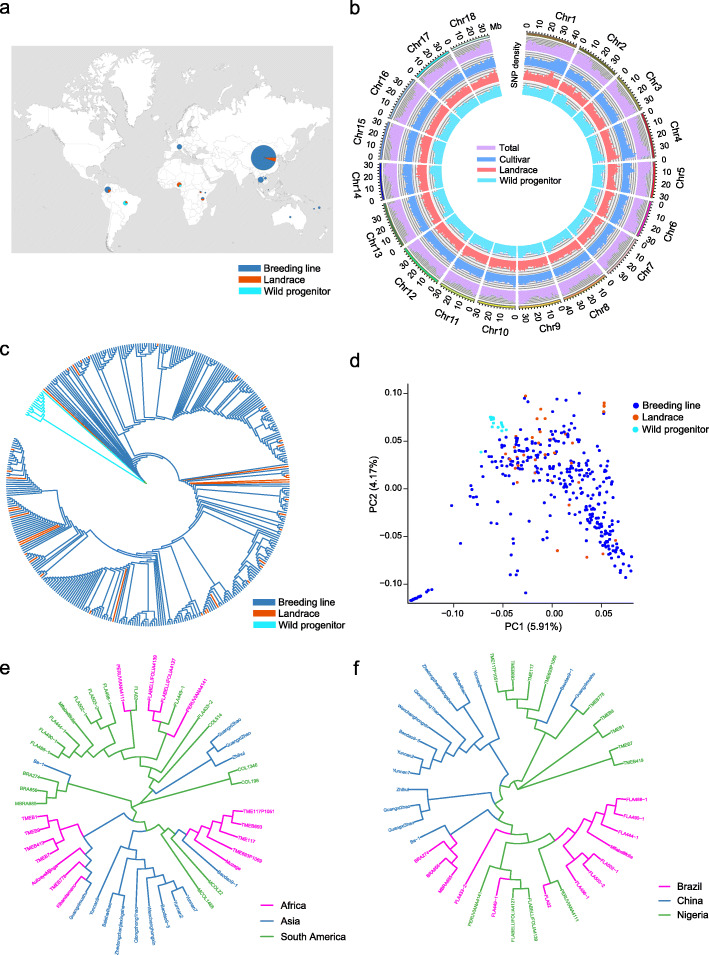
Genome-wide variation map and phylogenetic relationship of cassava. **a** Geographic distribution of resequenced cassava accessions. The area of the circle is proportional to the number of cassava accessions. **b** SNP density distributions in 10-kb non-overlapping sliding windows of 388 resequenced cassava accessions. **c** Phylogeny of 388 cassava accessions generated using the neighbor-joining tree method with genome-wide SNPs. **d** Principal component analysis of 388 cassava accessions using genome-wide SNPs. PC1, first principal component; PC2, second principal component. **e,f** Phylogenic relationships of wild progenitors and landraces from different continents (**e**) and countries (**f**) analyzed using the neighbor-joining tree method with genome-wide SNPs

A total of 388 cassava accessions, including 14 wild progenitors, 38 landraces, and 336 breeding lines were collected to construct a cassava variation map. These samples include 51 accessions that were previously resequenced [6, 24] and 337 accessions that were resequenced in the present study, representing accessions from the main cassava production areas of 15 countries worldwide (Fig. 1a). A total of 3.35 Tb data was generated with an average sequencing depth of 9.45× of the reference genome SC205 [26] (Additional file 1: Table S1). We identified a total of 1,344,463 high-quality single-nucleotide polymorphisms (SNPs) and 1,018,832 InDels (shorter than 50 bp) (Fig. 1b, Additional file 2: Table S2). We observed that the average accuracy of the SNP calling is 94.2% by PCR-based sequencing (Additional file 3: Table S3). Phylogenetic and principal component analyses (PCA) classified the 388 cassava accessions into two major groups (Fig. 1c,d). Group I includes 13 wild progenitors and group II contains all of the landraces and breeding lines and a single wild progenitor (FLA433-2) belonging to *M. esculenta* ssp. *flabellifolia* (Additional file 22: Fig. S1). Notably, the wild progenitor FLA433-2 from Brazil shows cassava-like storage roots and is genetically admixed with the cultivars [6]. Its close phylogenetic relationship with all of the cultivars supports the hypothesis of the origin of cassava in South America with *M. esculenta* ssp. *flabellifolia* representing the most likely progenitor species of cultivated cassava [6, 17, 24]. The LD extent in cassava was lower in the wild progenitors (7 kb; *r*^2^ = 0.2) than in the landraces (85 kb; *r*^2^ = 0.2) and breeding lines (177 kb; *r*^2^ = 0.2) (Additional file 22: Fig. S2).

We assessed genetic distances and phylogenetic relationship among populations of landraces and wild progenitors (Additional file 4: Table S4). On a continent scale, the values of *F*_ST_ and *p*-distance between cassava populations from South America and Africa (0.071; 0.189) and between populations from Africa and Asia (0.067; 1.017) were lower than those between populations from South America and Asia (0.094; 0.200). Cassava accessions from Africa clustered closely with the accessions from South America or Asia (Fig. 1e). On a country scale, the values of *F*_ST_ and *p*-distance between cassava populations from Brazil and Nigeria (0.120; 0.174) and between populations from Nigeria and China (0.088; 0.112) were lower than those between populations from Brazil and China (0.149; 0.189). Cassava accessions from Nigeria clustered closely with the accessions from Brazil or China (Fig. 1f). These results indicate a close phylogenetic relationship between cassava populations from South America and Africa as well as between populations from Africa and Asia. This is in accordance with archeological evidence showing that cassava was present in South America more than 4000 years ago, following which it was taken from Brazil to the Atlantic coast of Africa in the 1500s after Europeans arrived on the American continent, and then to Southeast Asia in the 1600s [27, 28].

### Genome-wide association study

Based on 1,313,775 high-quality SNPs from 337 cassava accessions resequenced in the present study, we performed a GWAS analysis for 33 morphological traits during 2013, 2016, and 2020 (Additional file 5: Table S5). Finally, we identified 52 marker-trait associations (MTAs) for 23 agronomically important traits (Additional file 6: Table S6, Additional file 22: Fig. S3). Of which, seven traits were detected MTAs in at least 2 years, and two MTAs for stem height and storage root (SR) number per plant were repeatedly observed during 2016 and 2020 (Additional file 7: Table S7, Additional file 22: Fig. S4). In addition, two MTAs for SR weight and SR number per plant overlapped with four known quantitative trait loci (QTLs) [29, 30] (Additional file 8: Table S8).

**Fig. 2:**
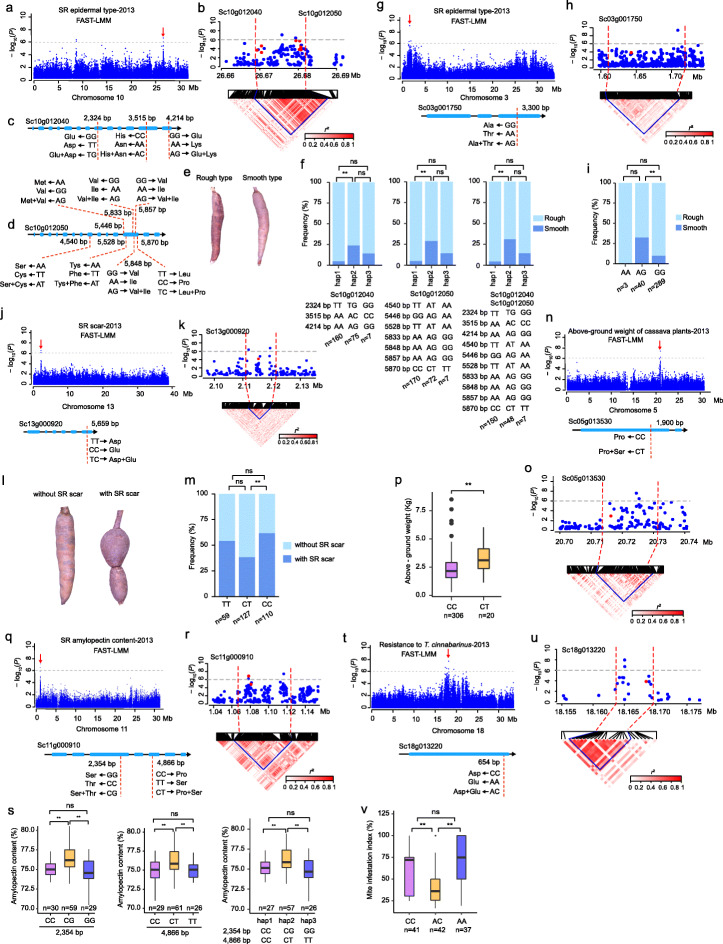
GWAS identification of candidate genes for variation in heterozygosity associated with key agronomic traits. **a–f** GWAS identification of *Sc10g012040* and *Sc10g012050* as candidate genes for SR epidermal type on chromosome 10. **a** Manhattan plots for SR epidermal type on chromosome 10 using FAST-LMM. Red arrow indicates the significant GWAS peak. **b** Local manhattan plot (top) and linkage disequilibrium heat map (bottom) surrounding the GWAS signal. Red dashed lines indicate the candidate region. Red dots indicate the core SNPs in the candidate gene. **c,d** *Sc10g012040* and *Sc10g012050* gene model, respectively. The dashed red lines represent the position of nonsynonymous SNPs. **e** Images of rough and smooth types of SR epidermis. **f** Comparison of SR epidermal types based on the haplotypes in *Sc10g012040* (left) and *Sc10g012050* (middle), as well as in both *Sc10g012040* and *Sc10g012050* (right). Hap 1 and hap 3 indicate the homozygous allele combinations, while hap 2 shows the heterozygous allele combinations. The symbol *n* represents the number of accessions with the same genotype. **g–i** GWAS identification of *Sc03g001750* as a candidate gene for SR epidermal type on chromosome 3. **j–m** GWAS identification of *Sc13g000920* as a candidate gene for SR scar on chromosome 13. **n–p** GWAS identification of *Sc05g013530* as a candidate gene for above-ground weight of cassava plants on chromosome 5. **q–s** GWAS identification of *Sc11g000910* as a candidate gene for SR amylopectin content on chromosome 11. **t–v** GWAS identification of *Sc18g013220* as a candidate gene for cassava resistance to *T*. *cinnabarinus* on chromosome 18. For box plots in (**p**), (**s**), and (**v**), the center line represents the median, box limits indicate the upper and lower quartiles, and whiskers denote the range of the data. The significance of difference was derived with two-tailed *t*-test (*P< 0.05, **P< 0.01). For histograms in (**f**), (**i**), and (**m**), the significance of difference was derived with the chi-square test (*P< 0.05, **P< 0.01)

Epidermal type (smooth or rough) and scar seriously affect the appearance quality of the SR. A significant signal on chromosome 10 associated with SR epidermal type was located at downstream of *Sc10g012040* and upstream of *Sc10g012050* (Fig. 2a,b), which encodes two auxin response factors that are crucial for various plant growth and development processes [31]. We found that nonsynonymous SNPs, three in *Sc10g012040* and seven in *Sc10g012050*, are associated with SR epidermal type (Fig. 2c,d)*.* Cassava accessions carrying the heterozygous alleles and heterozygous haplotype within *Sc10g012040* and *Sc10g012050* showed higher frequency of smooth epidermis of SR than those carrying the homozygous alleles and homozygous haplotypes (Fig. 2e,f and Additional file 22: Fig. S5, S6). In addition, *Sc03g001750* near another SR epidermal type signal on chromosome 3 encodes a mitochondrial carrier protein involved in plant development [32] (Fig. 2g,h). *Sc03g001750* contains a nonsynonymous SNP (3300 bp) that is associated with SR epidermal type. The accessions carrying the heterozygous AG allele had higher frequency of smooth epidermis of SR than did those with the homozygous GG allele (Fig. 2i). Moreover, a SR scar signal on chromosome 13 was located at downstream of *Sc13g000920* (Fig. 2j,k), which encodes an isopentenyl-diphosphate delta-isomerase that is essential for cytokinin biosynthesis [33]. A nonsynonymous SNP (5659 bp) in *Sc13g000920* is associated with SR scar. The accessions harboring the heterozygous CT allele showed lower frequency of SR with scar than those harboring the homozygous CC allele (Fig. 2l,m).

The weight of the above-ground part of cassava reflects the growth vigor of the plant and becomes an important factor in its use as feed. A significant signal associated with above-ground weight of cassava plants on chromosome 5 harbors *Sc05g013530* (Fig. 2n,o), which encodes a FAR1-related sequence protein that is known to regulate plant growth and development [34]. A nonsynonymous SNP (1900 bp) in *Sc05g013530* has two types of allelic variation (CC and CT). The accessions harboring the heterozygous CT allele showed significantly higher above-ground weight than did those harboring the homozygous CC allele (Fig. 2p).

SR amylopectin content determines the industrial value of cassava. *Sc11g000910* near a SR amylopectin content signal on chromosome 11 encodes a cytochrome P450 protein that is associated with starch accumulation during plant development [35] (Fig. 2q,r). We identified two nonsynonymous SNPs (2354 bp and 4866 bp) in *Sc11g000910*, which is associated with SR amylopectin content. Cassava accessions carrying the heterozygous alleles and heterozygous haplotype had higher SR amylopectin content than those carrying the homozygous alleles and homozygous haplotypes (Fig. 2s).

Storage roots with red endothelium may have high nutritional value and are popular among consumers. A strong peak on chromosome 2 associated with SR endothelial color harbors *Sc02g008280*, which encodes a glycosyltransferase that is required for anthocyanin biosynthesis [36] (Additional file 22: Fig. S7a,b). We found that a nonsynonymous SNP (522 bp) in *Sc02g008280* was associated with SR endothelial color*.* The accessions harboring the AT or TT allele had higher frequency of SR with red endothelium than those harboring the AA allele (Additional file 22: Fig. S7c,d,e).

Susceptibility to mites, especially for *Tetranychus cinnabarinus*, bring about up to 50~70% reduction in the production of cassava [37]. A peak associated with cassava resistance to *T. cinnabarinus* on chromosome 18 was located at the upstream of *Sc18g013220* (Fig. 2t,u), which encodes a heat-shock protein that is widely involved in response to biotic and abiotic stresses [38, 39]. A nonsynonymous SNP (654 bp) in *Sc18g013220* is associated with cassava resistance to *T. cinnabarinus*. The accessions harboring the heterozygous AC allele showed significantly lower mite infestation index than did those harboring the homozygous CC or AA allele (Fig. 2v).

We examined the expression levels of these candidate genes and found that all these genes expressed abundantly in different tissues or stages of SR development (Additional file 9: Table S9, Additional file 22: Fig. S8). Together with the above allelic variation analysis, these results suggest the potential roles of allelic variation in heterozygosity on key agronomic traits, of which cassava accessions carrying heterozygous alleles in each of these candidate gene had more desirable phenotype than did those carrying the corresponding homozygous alleles.

### Identification of highly heterozygous blocks

It is necessary to retain the heterozygous loci that control excellent traits during breeding. We identified 1395 heterozygous blocks (occupying 27.9 Mb) with high frequency (above the 95% threshold) in cultivars, defined as highly heterozygous blocks, involving 3718 genes (Additional file 10: Table S10, Additional file 22: Fig. S9a). Kyoto Encyclopedia of Genes and Genomes (KEGG) analysis suggested that “starch and sucrose metabolism” is the most significantly enriched group for these genes (Additional file 11: Table S11, Additional file 22: Fig. S9h). We characterized 79 highly heterozygous blocks that are associated with starch accumulation in the storage root (Additional file 12: Table S12). For example, *Sc02g005160* (encoding a glycogen phosphorylase) and *Sc02g005330* (encoding an alpha-amylase), which are involved in starch metabolism [40, 41], were located on the blocks w17969 and w17979, respectively. We further observed 20 highly heterozygous blocks that overlapped with 6 GWAS signals (Additional file 13: Table S13, Additional file 22: Fig. S9). Moreover, cassava accessions carrying the highly heterozygous block on chromosome 1 showed higher frequency of red endothelium of SR than those carrying the corresponding homozygous block (Additional file 22: Fig. S9i). These results further support the hypothesis that variation in heterozygosity is associated with key agronomic traits.

### Selection signatures of heterozygosity in cassava

**Fig. 3:**
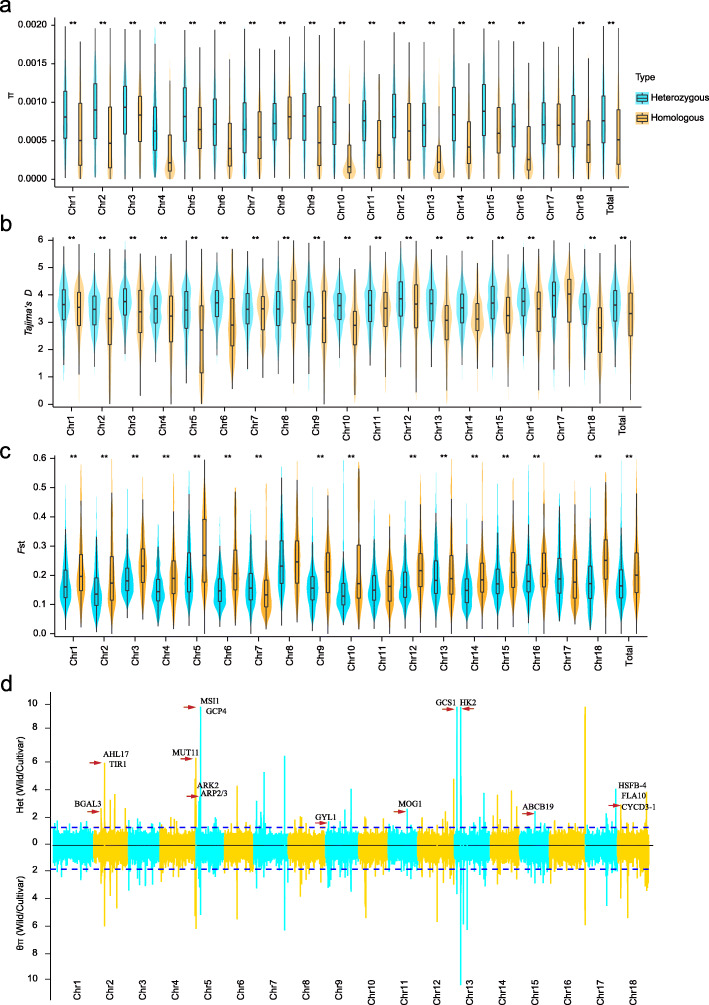
Selection signatures of heterozygosity in cassava. **a–c** Comparison of genomic diversity between heterozygous and homozygous regions using the values of *π* and Tajima’s *D* in 374 cultivars as well as *F*_ST_ value between 14 wild progenitors and 14 randomly selected cultivars. The significance of difference was derived with two-tailed *t*-test (*P < 0.05, **P < 0.01). **d** Selection signals for decrease in heterozygosity and nucleotide diversity from wild cassava progenitors to cultivars. The upper (1.27) and lower (1.86) horizontal blue lines indicate values above the 99% thresholds for selection sweeps in heterozygosity and nucleotide diversity, respectively. Red arrows indicate the positions of growth- and development-associated genes that are differentially expressed in storage roots between wild progenitors and cultivars within selective sweep regions with both decreases in heterozygosity and nucleotide diversity. Annotation of these genes is shown in Additional file 19: Table S19

To trace the possible evolutionary scenario of heterozygosity, we split the cassava genome into heterozygous and homozygous regions according to heterozygous SNPs of the reference gnome. We then compared the genomic diversity between heterozygous and homozygous regions in 374 cultivars. The heterozygous regions possessed significantly higher genetic diversity (*π* value) and positive Tajima’s *D* value than the homozygous regions in most (16 out of 18) chromosomes, suggesting that balancing selection has contributed to the maintenance of genomic heterozygosity. This finding is also supported by the lower *F*_ST_ value in heterozygous regions than in homozygous regions in most (14 out of 18) chromosomes between cultivars and wild progenitors (Fig. 3a–c, Additional file 14: Table S14).

Artificial selection is of great importance for domestication of crops for desirable phenotypic traits [42]. To identify potential signals of selection related to heterozygosity during cassava domestication, we scanned genomic regions that exhibit drastic decreases in heterozygosity above the 99% threshold by comparing wild progenitors and cultivars (including landraces and breeding lines) and identified 140 selective sweeps that cover 1.65% (11.7 Mb) of the assembled genome and harbor 941 protein-coding genes (Fig. 3d, Additional file 15: Table S15). We also identified 138 selective sweeps (above the 99% threshold) that show reduction in nucleotide diversity by comparing wild progenitors and cultivars, covering 11.6 Mb of the assembled genome and harboring 851 protein-coding genes (Fig. 3d, Additional file 16: Table S16). We found 81 selective sweeps with both decreases in heterozygosity and nucleotide diversity, covering 6.5 Mb of the assembled genome and harboring 548 protein-coding genes (Fig. 3d, Additional file 17: Table S17). These genes were enriched in multiple biological processes including growth, development, hormone metabolisms and responses, and immune-related processes (Additional file 18: Table S18).

### Selection for decreases in heterozygosity contributes to domestication of the large starchy storage root in cassava

**Fig. 4:**
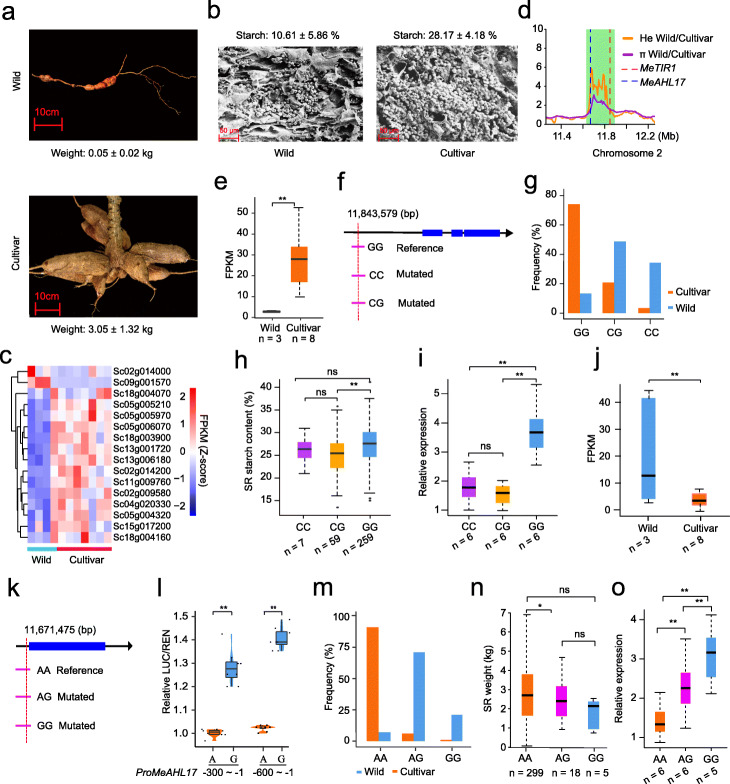
Selection for decrease in heterozygosity associated with domestication of the large starchy storage root (SR) of cassava. **a** Morphological differences in SR between wild and cultivated cassavas after 350 d of cultivation. **b** Differences in starch content of SR between wild progenitors and cultivars after 350 d cultivation. **c** Differences between wild progenitors and cultivars in the expression of genes identified in selection sweeps (above 99% threshold) and related to growth and development in SR at 350 days after planting. Annotation of these genes is shown in Additional file 19: Table S19. **d** Selection signal detected in *MeTIR1* and *MeAHL17*. The green rectangle indicates the selection signal region. The dashed red and blue lines represent the position (chr2:11,843,579) of a core SNP in the upstream of *MeTIR1* and the position (chr2: 11,671,475) of a core SNP in the upstream of *MeAHL17*, respectively. **e** Comparison of *MeTIR1* expression levels (FPKM) in SR between wild progenitors and cultivars at 350 days after planting. **f** *MeTIR1* gene model. The dashed red line represents the position of the core SNP in the upstream region of *MeTIR1*. **g** Frequency of the SNP variants in *MeTIR1* in wild and cultivated cassavas. **h** Box plots of SR starch content based on the SNPs of CC, CG, and GG. The symbol *n* represents the number of accessions with the same genotype. **i** Differences in expression of *MeTIR1* alleles in SR at 350 d after planting among CC, CG, and GG containing cultivars. **j** Comparison of *MeAHL17* expression levels in SR at 350 days after planting between wild progenitors and cultivars. **k** *MeAHL17* gene model. The dashed red line represents the position of the core SNP in the promoter region of *MeAHL17*. **l** Comparison of activities between A-containing and G-containing *MeAHL17* promoter regions (300 bp and 600 bp upstream of transcription start site) in dual luciferase assay. Each sample contains 8 biological replicates. **m** Frequency of the SNP variants in the *MeAHL17* gene in wild and cultivated cassavas. **n** Box plots of SR weight based on the SNPs of AA, AG, and GG. The symbol *n* represents the number of accessions with the same genotype. **o** Differences in expression of *MeAHL17* in SR at 350 days after planting among AA, AG, and GG containing cultivars. The data in **e** and **j** was detected by RNA-Seq and the data in **i** and **o** was examined by qRT-PCR. The symbol *n* represents the number of accessions. Each accession contains three biological replicates. The data for biological replicates were used to draw the box plots. For **e**, **h**, **i**, **j**, **l**, **n**, and **o**, the center line represents the median, box limits indicate the upper and lower quartiles, and whiskers denote the range of the data. The significance of difference in this figure was derived with two-tailed *t*-test (*P < 0.05, **P < 0.01)

Domestication has led to significant increases in yield and starch content of cassava storage roots. Storage root weight and starch content in cultivars are about 60 and 3 times higher than in wild progenitors, respectively (Fig. 4a, b). To understand the mechanism underlying this phenomenon, we identified growth- and development-associated genes that are differentially expressed in storage roots between wild progenitors and cultivars within selective sweep regions with both decreases in heterozygosity and nucleotide diversity, including *MeTIR1* (*Sc02g014200*) and *MeAHL17* (*Sc02g014000*) (Fig. 3d, Fig. 4c, and Additional file 19: Table S19).

*MeTIR1* is an ortholog of *TIR1* in *Arabidopsis*, which encodes a transport inhibitor response 1 protein and functions as the long-sought auxin receptor [43]. Auxin biosynthesis and *TIR1*-mediated auxin signaling are required for starch synthesis [44, 45]. We found a selection signal for decreased heterozygosity around *MeTIR1* on chromosome 2 (Fig. 4d). Expression of *MeTIR1* was significantly higher in cultivars compared to wild progenitors (Fig. 4c, e). We found that a SNP in the upstream region (− 5067 bp) of *MeTIR1* might be associated with *MeTIR1* expression and domestication (Fig. 4f). Compared with wild progenitors, cultivars showed a lower frequency of the CG and CC alleles, but a higher frequency of the homozygous GG allele (Fig. 4g). Moreover, starch content was significantly higher in accessions carrying the GG allele than in accessions carrying the CG allele (Fig. 4h). Accordingly, *MeTIR1* exhibited significantly higher expression in accessions carrying the GG allele than in accessions carrying the CG and CC alleles (Fig. 4i). We further validated the function of *MeTIR1* in cassava using transient overexpression and silencing technology and found that overexpression of *MeTIR1* in F1015 (GG allele), R72 (GG allele), 4363 (CG allele), and Baodao9-1 (CG allele) significantly increased starch content, whereas silencing of *MeTIR1* in F1015 (GG allele) and R72 (GG allele) led to decreased starch content in cassava leaves (Additional file 22: Fig. S10).

In addition, we found a strong selection signal for decreased heterozygosity around *MeAHL17* on chromosome 2 (Fig. 4d). *MeAHL17*, an ortholog of *AHL17* in *Arabidopsis*, encodes an AT-hook motif nuclear-localized transcription factor that is associated with inhibiting diverse developmental processes [46–48]. Expression of *MeAHL17* was significantly lower in cultivars than in wild progenitors (Fig. 4c, j). Further, we found that a SNP in the upstream region (-53 bp) of *MeAHL17* might be associated with *MeAHL17* expression and domestication (Fig. 4k)*.* The dual luciferase assay indicated that the promoter regions of *MeAHL17* carrying G had higher activity than those carrying A, suggesting that this allelic variation affects *MeAHL17* expression (Fig. 4l). Compared with wild progenitors, cultivars showed a lower frequency of AG and GG alleles, but a higher frequency of the homozygous AA allele (Fig. 4m). Storage root weight was significantly higher in accessions carrying the AA allele than in accessions carrying the AG allele (Fig. 4n), which is significantly associated with lower *MeAHL17* expression in accessions carrying AA than in those carrying AG (Fig. 4o).

### *MeAHL17* is positively associated with cassava bacterial blight (CBB) susceptibility

**Fig. 5:**
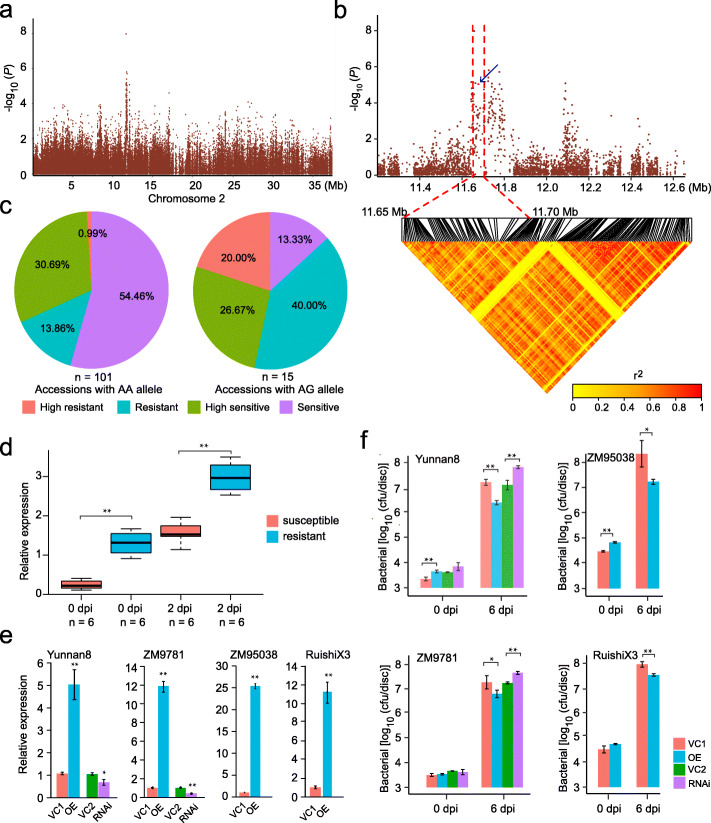
Identification of the candidate gene *MeAHL17* associated with cassava bacterial blight (CBB). **a** Manhattan plots for CBB resistance on chromosome 2 using FAST-LMM. **b** Local manhattan plot (top) and linkage disequilibrium heat map (bottom). Red dashed lines indicate the candidate region. Blue arrow indicates the SNP in the candidate gene. **c** Comparison of CBB resistance based on the SNPs of AA and AG. The symbol *n* represents the number of accessions with the same genotype. **d** Differences in expression of *MeAHL17* between CBB-susceptible accessions (carrying AA allele) and CBB-resistant accessions (carrying AG allele) at (0 days post inoculation (dpi)) and 2 dpi of *Xam* infection, as detected by qRT-PCR (**P < 0.01; two-tailed *t*-test). The symbol *n* represents the number of accessions. Each accession contains three biological replicates. The center line represents the median, box limits indicate the upper and lower quartiles, and whiskers denote the range of the data. **e** Expression of *MeAHL17* in cassava leaves transformed with pCAMBIA1304 (vector control, VC1), pCAMBIA1304::MeAHL17 (OE), pTRV (vector control, VC2), or pTRV::MeAHL17 (RNAi) in four cultivars, respectively, as detected by qRT-PCR (*P < 0.05, **P < 0.01; two-tailed *t*-test). Data are represented as mean ± s.d. (*n* = 3 biological replicates). **f** The bacterial number in cassava leaves transformed with pCAMBIA1304 (vector control, VC1), pCAMBIA1304::MeAHL17 (OE), pTRV (vector control, VC2), or pTRV::MeAHL17 (RNAi) in four cultivars at 0 and 6 days post inoculation (*P < 0.05, **P < 0.01; two-tailed *t*-test). Data are represented as mean ± s.d. (*n* = 4 biological replicates)

Cassava bacterial blight (CBB), caused by *Xanthomonas axonopodis* pv. *manihotis* (*Xam*), is one of the most severe threats to cassava production [49]. We found a strong association signal on chromosome 2 with a − log_10_*P* value of 7.97 (Fig. 5a). We focused on the locus mapped from 11.65 to 11.70 Mb with six candidate genes (*Sc02g013980* through *Sc02g014030*) (Fig. 5b), of which only *MeAHL17* showed significant induction after *Xam* inoculation (Additional file 20: Table S20). In chickpea, an *AHL* member was mapped to be associated with Ascochyta blight resistance [50]. We found that the same allelic SNP variation as shown in Fig. 4k in the promoter region of *MeAHL17* is associated with CBB resistance. The accessions carrying the AA allele of *MeAHL17* showed significantly higher frequency of susceptibility to CBB than those carrying the AG allele (Fig. 5c). Although the transcripts of *MeAHL17* in all 12 accessions were largely induced after *Xam* infection at 2 days post inoculation (dpi), the transcripts of *MeAHL17* in CBB-susceptible accessions (carrying the AA allele) were significantly lower than those in CBB-resistant accessions (carrying the AG allele) at both 0 dpi and 2 dpi (Fig. 5d). Moreover, overexpression of *MeAHL17* in Yunnan8 (with AG allele), ZM9781 (with AG allele), ZM95308 (with AA allele), and RuishiX3 (with AA allele) increased CBB resistance by leaf bacterial populations, whereas silencing of *MeAHL17* in Yunnan8 (with AG allele) and ZM9781 (with AG allele) increased CBB susceptibility (Fig. 5e, f and Additional file 22: Fig. S11). These results suggest that *MeAHL17* is positively associated with CBB resistance.

### Combinations of allelic variation in *MeTIR1* and *MeAHL17*

**Fig. 6:**
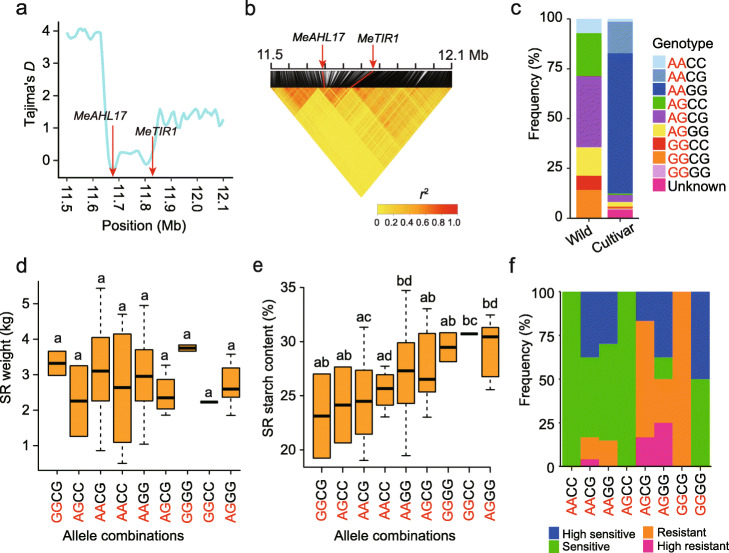
Effects of *MeAHL17* and *MeTIR1* allele combinations on trait variation. **a** Tajima’s *D* distribution surrounding *MeAHL17* and*MeTIR1* loci. **b** Linkage disequilibrium heat map surrounding *MeAHL17* and *MeTIR1* loci. Red lines indicate the candidate region. **c** Allele distribution of *MeAHL17* at position 11,671,475 (Red letters) and *MeTIR1* at position 11,843,579 (Black letters) on chromosome 2 in wild and cultivated cassavas. **d**, **e** Box plots of SR weight (**d**) and SR starch content (**e**) based on indicated allele combinations. The center line represents the median, box limits indicate the upper and lower quartiles, and whiskers denote the range of the data. Pairwise multiple comparisons (median test, adjusted *P* < 0.05) were used to identify significant differences shown with different letters and no significant differences shown with the same letter. **f** Comparison of frequency of CBB resistance based on the indicated allele combinations. The black and red letters in **d**, **e**, and **f** indicate the same as in **c**

*MeAHL17* and *MeTIR1* are located within the same selective sweep region on chromosome 2 (Figs. 4d and 6a). We performed a linkage disequilibrium analysis of *MeAHL17* and *MeTIR1* loci and found that they are tightly linked (Fig. 6b). The frequency of allelic combinations of AA (*MeAHL17*) and GG (*MeTIR1*) as well as AA (*MeAHL17*) and CG (*MeTIR1*) significantly increased during domestication (Fig. 6v). However, the starch content and the frequency of resistance to CBB were not the most desirable in cultivars carrying the two allelic combinations (Fig. 6d, e, f). We also found that cultivars carrying the combinations of AG (*MeAHL17*) and GG (*MeTIR1*) alleles showed high starch content and high frequency of resistance to CBB (Fig. 6e, f)*.*

## Discussion

Generation of heterozygous genomes by hybridization between or within species can help maintain plant diversity and serve as a potential source of new species [51]. Cassava is monoecious with unisexual flowers, which lead to out crossing that increase genomic heterozygosity. Extensive hybridization and clonal reproduction yield and maintain the high heterozygosity of cassava [6]. Balancing selection has contributed to the maintenance of cassava heterozygosity, which is in accordance with the role of balancing selection on retaining genetic diversity [52–54]. In contrast, comparison of wild accessions and cultivars revealed 81 selective sweeps with both decreases in heterozygosity and nucleotide diversity, suggesting that decrease in nucleotide diversity by artificial selection is accompanied with reduction in heterozygosity during cassava domestication. Modern cassava breeding has primarily focused on yield and starch content of storage roots [24]. Decreases in both heterozygosity and nucleotide diversity of *MeTIR1* and *MeAHL17* by artificial selection contributed to the domestication of the large starchy storage root. However, the selection of the homozygous genotype of *MeAHL17* led to the adverse effect of CBB susceptibility. The allele combinations of *MeTIR1* and *MeAHL17* resulting from variation in heterozygosity may confer high starch content and resistance to CBB, which will be beneficial for future cassava breeding.

Artificial selection has shaped crop genomes to meet human demands and has also resulted in the near fixation of a large proportion of the genome [2, 55]. There is an urgent need to utilize genetic diversity in germplasm resources for crop improvement [56]. Effective screening of non-selected regions could provide favorable alleles to use for retaining genetic diversity and future breeding. Several promising candidate genes that were found to be highly associated with key traits of cassava from GWAS analysis are in non-selected regions (Fig. 2). Cassava accessions carrying the heterozygous alleles in each candidate gene showed more desirable phenotype than did those carrying the corresponding homozygous alleles. Additionally, we characterized 79 highly heterozygous blocks associated with starch accumulation as well as a highly heterozygous block associated with red endothelium of storage root. Retaining of these heterozygous alleles and blocks during future breeding may be beneficial for excellent agronomic traits and genetic diversity of cassava. This deepens our understanding of variation in heterozygosity affecting agronomic traits and provides potential targets for selection during cassava breeding.

The availability of genomic resources is not in itself sufficient to increase crop productivity [56]. It is important for crop improvement to systematically evaluate germplasm resources and access allelic variations affecting agronomic traits. With the release of the cassava genome, the research on population genetics of cassava reveals several fundamental issues in cassava biology, especially the nature of its extensive interspecific hybridization and fixation of deleterious mutations during clonal propagation [6, 24]. Currently, the use of large-scale resequencing data to precisely identify valuable loci associated with key agronomic traits of cassava remains poorly understood. Resequencing 388 cassava accessions yielded 1,344,463 high-quality SNPs. These comprehensive data and the high-density genetic markers offer adequate coverage to investigate association between markers and causal loci controlling agronomic traits, as previously reported in rice [3], cotton [5], tomato [2], and soybean, etc [12]. We detected 52 loci associated with 23 agronomic traits of cassava by GWAS analysis with multiple statistical models. Of which, the allelic variation in heterozygosity of nine candidate genes associated with key agronomic traits were identified and the orthologs of several candidate genes, such as *Sc10g012040*, *Sc10g012050*, *Sc13g000920*, and *Sc02g014000*, have been functionally characterized in other species. In addition, it is possible to identify association between allelic variation of homozygous genotypes and agronomic traits from significant GWAS signals. The data obtained from resequencing and GWAS will facilitate future functional genomic studies and designing breeding strategies in cassava.

Taken together, this study identified valuable loci with variation in heterozygosity associated with key agronomic traits and offered insights into variation in heterozygosity during cassava domestication, which provides a large amount of new genomic resources for utilization of heterozygosity to accelerate cassava improvement. Further work will be required to validate candidate genes underlying the key traits because of the limited phenotyping replicates for GWAS analysis. In addition, more wild progenitors should be collected and resequenced to further verify the accuracy of our domestication analysis due to the limited number of wild progenitors used in this study.

## Conclusions

This study provided a variation map of 388 cassava accessions, identified 52 loci for 23 agronomic traits, and revealed allelic variation in heterozygosity associated with key agronomic traits and cassava domestication. We found that artificial selection of homozygous alleles in *MeTIR1* and *MeAHL17* contributes to the domestication of the large starchy storage root. Notably, selection of homozygous alleles in *MeAHL17* is associated with increased both storage root weight and CBB susceptibility. Our findings will contribute to elucidating the genetic basis of the variation in heterozygosity associated with key agronomic traits and cassava domestication as well as allow for the strategic exploitation for cassava improvement. This should facilitate breeding programs, not only in cassava but also in other highly heterozygous crops.

## Methods

### Sampling and whole-genome resequencing

A total of 337 cassava accessions, including 20 landraces and 317 breeding lines, were collected from the Chinese Cassava Germplasm Bank (Danzhou, China) for whole-genome resequencing. The plants grew in the planting base at the Tropical Crops Genetic Resources Institute of Chinese Academy of Tropical Agricultural Sciences. Fresh young leaves were frozen in liquid nitrogen for genomic DNA extraction using a DNeasy Plant Mini Kit (Qiagen, Beijing, China). Paired-end libraries with insert size of 500 bp were constructed with 5 μg of genomic DNA for each sample. The 150-bp paired-end reads for each sample were sequenced using the Illumina X-Ten platform. The raw data of genome resequencing was deposited in the Sequence Read Archive under a NCBI BioProject accession PRJNA578024 [57]. The other 38 cassava accessions previously resequenced by Bredeson et al. [6] and 13 accessions previously resequenced by Ramu et al. [24] were downloaded from NCBI-SRA (https://www.ncbi.nlm.nih.gov/) and Cassava Base (ftp://ftp.cassavabase.org/HapMapII/), respectively.

### Reads alignment, variation calling, and SNP validation

The sequencing reads for each sample were mapped to the cassava SC205 reference genome in the BWA mem v0.7.17 [58] program with the default parameters. The mapping results were processed by sorting and duplicate marking using Samtools v1.9 [59] and Picard v1.94 (https://broadinstitute.github.io/picard/). After removing the low mapping quality (MQ < 20) reads, both single-end and paired-end mapped reads were used to detect SNPs in the GATK toolkitv3.5 [60] process. Mapped reads resulting from PCR duplicates were removed. The HaplotypeCaller module was used to establish a raw population genotype file containing the SNPs and indels that was further filtered using the parameters: “QUAL < 2.0 || QD < 2.0 || MQ < 40.0 || FS > 60.0 || MQRankSum < -12.5 || ReadPosRankSum < -8.0 -clusterSize 2 -clusterWindowSize 5” and “QD < 2.0 || FS > 200.0 || ReadPosRankSum < -20.0.” The annotation of the identified SNPs and indels were performed using SnpEff v3.6c tool software [61]. A total of 26 SNPs were randomly selected for PCR-based sequencing from 33 accessions. All PCR products were aligned against the target region of SC205 genome using Sequencher 5.4, and the genotypes were manually checked for each accession.

### Phylogenetic analysis

After filtering the low-quality SNPs using metrics of “minor allele frequency (MAF) > 0.05 and integrity > 0.8” from the entire SNP dataset, we obtained a subset of 1,344,463 high confidence SNPs for phylogenetic analysis. To investigate the phylogenetic relationships of the 388 cassava accessions, an unrooted phylogenetic tree was constructed using the neighbor-joining method with the Kimura 2-parameter model and 1000 bootstrap replicates in MEGA5.10 [62]. The consensus trees were exhibited with the online Interactive tree of life (iTOL) v3 (https://itol.embl.de) [63].

### Principal component analysis and linkage disequilibrium analysis

Principal component analysis (PCA) was carried out using the smartPCA program from the EIGENSOFT package v.6.0.1 (https://github.com/DReichLab/EIG) with 1,344,463 high confidence SNPs. The first two principal components were used to separate the cultivar and wild progenitor samples. Linkage disequilibrium between pairs of SNPs with MAF > 0.05 and integrity > 0.8 was assessed as the correlation coefficient (*r*^2^) using PLINKv1.07 with the parameters (--maf 0.05 --r2 --ld-window 999999 --ld-window-kb 1000 --ld-window-r20) [64]. LD decay was estimated based on *r*^2^ between two SNPs and averaged in 1-kb windows with a maximum distance of 1 Mb.

### *F*-statistic and *p*-distance calculation

The fixation statistic *F*_ST_ and *p*-distance were used to estimate the degree of pairwise genomic differentiation between pairs of populations, based on the variance in allele frequencies. The *F*_ST_ values between different geographical groups were evaluated using PopGenome package v2.7.1 (https://popgenome.weebly.com/). The *p*-distance values between different groups were estimated using MEGA X with the *p*-distance model [65].

### Phenotyping

A total of 337 accessions were planted in Danzhou, Hainan Province (109.5 E, 19.5 N). The cassava plants were planted in early March 2013/2016 and harvested in late February 2014/2017, as well as planted in early May 2020 and harvested in early May 2021. Each accession was grown in one row that consisted of eight plants with 0.8 m distance between each plant. Phenotyping of whole plant-, stem-, storage root-, postharvest-, and resistance-related traits was performed according to the Chinese description specification for cassava germplasm resources (NY1943-2010) in 2013, 2016 and 2020. The phenotypic data of plant height, biomass, stem diameter, stem height, above-ground weight, SR weight, SR length, and SR number for each accession were defined as the average of the eight plants. The SR starch content, SR starch viscosity, SR starch gelatinization temperature, and SR amylopectin content were examined according to previous methods [66]. The data for each accession were defined as the mean of three biological replicates. Resistance to *T. cinnabarinus* and *Xam* of cassava accessions was evaluated following the Chinese technical specification for evaluating cassava resistance to pests (NY/T 2445-2013) and bacterial blight (NY/T 3005-2016). The mean value from three biological replicates was employed to determine the resistance levels.

### Genome-wide association study

In total, 1,313,775 high-quality SNPs with a minor allele frequency (MAF) > 0.05 and integrity > 0.8 in the 337 cassava cultivars were used for GWAS of 33 traits. The GWAS analyses were performed using different statistical models of generalized linear model (GLM), mixed linear model (MLM), compressed mixed linear model (CMLM), efficient mixed model association expedited (EMMAX), and Factored Spectrally Transformed Linear Mixed Models (FAST-LMM). The Q matrix was generated by ADMIXTURE v1.3.0 [67] as fixed effects. In the MLM and CMLM model, the kinship (K) matrix was constructed using SPAGeDi v1.3a [68]. The *P* value threshold of 0.000001 was set to control the genome-wide type I error rate [69]. MTAs supported at least by two statistical models are defined as valid MTAs. Genes located in 100-kb windows which centered on the MTAs were considered as potential candidate genes for further analysis. The potential candidate genes that have annotation and transcripts were selected for further analyzing whether they are in the LD block region and have allelic variation associated with phenotypic differences.

### RNA sequencing and expression quantification

Three biological replicates were included in each RNA-Seq experiment. The root, stem, leaf samples, and the storage root at seven development stages from 100 to 340 DAP (days after planting) were collected from SC205 for RNA-Seq experiments. We also sampled the storage roots from three wild progenitors and eight cultivars for transcriptome sequencing. The RNAs were isolated using the RNeasy Plus Mini kit DP441 (Qiagen, Beijing, China), and cDNA libraries were prepared using an NEBNext Ultra RNA Library Prep Kit. The quantified libraries were then prepared for sequencing using the Illumina HiSeq X-Ten platform. Adapter sequences and low-quality reads (more than 20% low-quality bases) were removed. The clean reads were mapped to the SC205 reference genome by HISAT2v2.0.4 [70]. The gene expression levels were calculated by StringTie v1.3.4d [71] using the default parameters. The gene expression level was normalized by the fragments per kilobase of transcript per million mapped reads (FPKM). The expressed genes were defined as FPKM values greater than 0.1. Differentially expressed genes (DEGs) were identified using the DEseq2 package [72]. Significant differences in expression levels were tested using a two-tailed *t*-test. The DEGs were defined as a fold change (FC) larger than 2 or smaller than 0.5 and a false discovery rate (FDR) < 0.01. The raw data of transcriptomic sequencing was deposited in the Sequence Read Archive under a NCBI BioProject accession PRJNA578024 [57].

### Identification of heterozygous blocks with high frequency in cultivars

The heterozygosity of each 20 kb non-overlapping window was calculated as “heterozygosity SNPs / window length” for each cultivar accession. The average heterozygosity of the windows was used as a cutoff value to distinguish heterozygous or homologous windows. The window that has more heterozygous sites than the average heterozygous SNPs (10.83 heterozygous SNPs per window) was considered as a heterozygous block in corresponding accession, while the contrary was regarded as a homologous block. We calculated the population frequency of the heterozygous blocks in 374 cultivars to identify the highly heterozygous blocks. The sliding windows within the empirical top 5% of heterozygous frequency in the population were selected as highly heterozygous blocks. For each highly heterozygous block region, we separated the 374 cultivars into heterozygous and homologous groups according their heterozygosity information. We then detected trait segregation using trait values between heterozygous and homologous groups in highly heterozygous blocks using the Wilcoxon rank-sum test method. The genes in the highly heterozygous blocks associated with excellent traits were manually investigated according the functional description of their orthologs in the *Arabidopsis* database.

### Genetic diversity analysis between heterozygous and homozygous regions

We remapped Illumina short reads of SC205 to its own reference genome and called SNPs using BWA mem v0.7.17 [58] and GATK toolkitv3.5 [60]. The rate of heterozygous SNPs for each window was calculated by sliding windows as formula: heterozygous SNPs / window length × 100% (window = 25 kb and step = 5 kb). The window that has the rate of heterozygous SNPs less than 0.002 is considered as homozygous region; otherwise, it is considered as heterozygous region. The adjacent homozygous or heterozygous windows were merged into one large region, respectively. Only the regions whose length are more than 50 kb were used for genetic diversity analysis. We compared the genomic diversity between heterozygous and homozygous regions using the values of *π* and Tajima’s *D* in 374 cultivars as well as *F*_ST_ value between 14 wild progenitors and 14 randomly selected cultivars at the genome and chromosome levels. The *π*, Tajima’s *D*, and *F*_ST_ values in each chromosome were calculated using PopGenome package v2.7.1 [73] (https://popgenome.weebly.com/) for 50-kb sliding windows with a 10-kb step size. The two-tailed *t*-test was used to examine the significant differences of genetic diversity between heterozygous and homozygous regions.

### Genome-wide selective sweep analysis

All of the SNPs with a minor allele frequency (MAF) > 0.05 and integrity > 0.8 were selected for selective sweep analysis. Expected heterozygosity (He) and nucleotide diversity (*π*) analyses were applied to estimate the degree of variability based on the variance in allele frequencies of wild progenitors and cultivars (including landraces and breeding lines). The expected heterozygosity (He) of each polymorphism site was calculated as 1 − *Σ P*_*i*_^2^, where *P*_*i*_ represents the frequency of the *i* allele. To detect the selective signals, the average He of each window was calculated for 50-kb sliding windows with a 10-kb step size along each chromosome. Additionally, *π* was calculated using PopGenome packagev2.7.1 [73] (https://popgenome.weebly.com/) for 50-kb sliding windows with a 10-kb step size along each chromosome. Sliding windows within the empirical top 1% of He (wild/cultivar) or *π* (wild/cultivar) values for each comparison were identified as the candidate selective sweep regions, respectively. Adjacent confident windows were merged into a single selective sweep region.

### Functional characterization of *MeTIR1* and *MeAHL17* in cassava

To explore the effect of the allele variation (at − 53 bp upstream of *MeAHL17*) on the activity of *MeAHL17* promoter, we performed a dual luciferase assay in cassava protoplasts. The promoter sequences (from 0 to − 300 bp and from 0 to − 600 bp) of *MeAHL17* carrying G or A were cloned and inserted into the pGreenII0800-LUC vector. The recombinant constructs were transformed into cassava protoplasts using previous described method [74]. We examined the relative luciferase activity using a Dual Luciferase Reporter Gene Assay Kit (RG027, Beyotime, Shanghai, China). To detect the expression profiles of *MeAHL17* in response to *Xam*, cassava leaves were collected at different time points. Total RNA was isolated and reverse transcribed using a cDNA Synthesis Kit (K1622, Thermo Scientific, Waltham, MA, USA). Then, the relative expression level of *MeAHL17* was qualified by quantitative real-time PCR (qRT-PCR) using the 2^–ΔΔCt^ method with *MeEF1a* as an internal reference. To overexpress *MeTIR1* or *MeAHL17*, the coding sequences of *MeTIR1* and *MeAHL17* were amplified from SC205 and inserted into the pCAMBIA1304 vector, respectively. An *Agrobacterium tumefaciens* strain (GV3101) that harbors the recombinant constructs or pCAMBIA1304 was syringe infiltrated into cassava leaves as previously described [75]. To silence *MeTIR1* or *MeAHL17*, *MeTIR1-* and *MeAHL17*-specific regions were amplified and cloned into the pTRV2 vector. An *Agrobacterium tumefaciens* strain (GV3101) that harbors the recombinant construct or pTRV2, together with pTRV1, were syringe infiltrated into cassava leaves as previously described [751]. After 2 days of cultivation for pCAMBIA1304::MeTIR1 and pCAMBIA1304 transformed plants and 2 weeks of cultivation for pTRV::MeTIR1 and pTRV transformed plants, we collected the cassava leaves to examine gene expression levels by qRT-PCR and starch content using a Starch Assay Kit (A148-1-1, Jiancheng, Nanjing, China). After 2 days of cultivation for pCAMBIA1304::MeAHL17 and pCAMBIA1304 transformed plants and 2 weeks of cultivation for pTRV::MeAHL17 and pTRV transformed plants, *Xam* was inoculated into the cassava leaves for 6 days, following which we examined the bacterial number and gene expression levels. The primer pairs used for qRT-PCR analysis are shown in Additional file 21: Table S21.

## Supplementary Information

**Additional file 1: Table S1.** All cassava accessions resequenced.

**Additional file 2: Table S2.** Genome-wide SNP and InDel distributions.

**Additional file 3: Table S3.** SNP accuracy verified using PCR-based sequencing.

**Additional file 4: Table S4.** Cassava accessions of wild progenitors and landraces resequenced.

**Additional file 5: Table S5.** Agronomical traits investigated in this study.

**Additional file 6: Table S6.** All significant SNP signals for the investigated traits (-log10P > 6).

**Additional file 7: Table S7.** MTAs for 7 traits identified in at least two years of phenotyping data.

**Additional file 8: Table S8.** Overlaps of known QTLs with GWAS signals for two yield associated traits.

**Additional file 9: Table S9.** Expression of candidate genes from GWAS analysis in different tissues and stages of storage root development.

**Additional file 10: Table S10.** Genomic regions and genes that showed heterozygosity with high frequency (Top 0.95) in 374 cultivars.

**Additional file 11: Table S11.** KEGG enrichment of the genes in the genomic regions that showed heterozygosity with high frequency (Top 0.95) in 374 cultivars.

**Additional file 12: Table S12.** Comparation of starch content between accessions carrying heterozygous blocks with high frequency and accessions carrying homozygous blocks in cultivars.

**Additional file 13: Table S13.** Overlapping of GWAS peaks and highly heterozygous blocks.

**Additional file 14: Table S14.** Comparison of genomic diversity between heterozygous and homozygous regions in population.

**Additional file 15: Table S15.** Genomic regions and genes that exhibit a decrease in heterozygosity during domestication from wild progenitors to cultivars (Top 0.99).

**Additional file 16: Table S16.** Genomic regions and genes that exhibit a decrease in nucleotide diversity during domestication from wild progenitors to cultivars (Top 0.99).

**Additional file 17: Table S17.** Genomic regions and genes that exhibit a decrease in both heterozygosity and nucleotide diversity during domestication from wild progenitors to cultivars (Top 0.99).

**Additional file 18: Table S18.** GO enrichment of the genes that exhibit a decrease in both heterozygosity and nucleotide diversity during domestication from wild progenitors to cultivars (Top 0.99).

**Additional file 19: Table S19.** Expression of the genes related to growth and development within selection sweeps with both decreases in heterozygosity and nucleotide (above 99% threshold) during cassava domestication.

**Additional file 20: Table S20.** Expression levels of the six genes after *Xam* inoculation within the locus mapped from 11.65 to 11.70 Mb.

**Additional file 21: Table S21.** Primer pairs used for qRT-PCR.

**Additional file 22: Fig. S1-S11. Fig. S1.** Phylogeny of 388 cassava accessions generated using the neighbor-joining tree method with genome-wide SNPs. **Fig S2.** Linkage disequilibrium (LD) decay for different groups. **Fig. S3.** Manhattan plots for GWAS analysis of cassava agronomic traits. **Fig. S4.** Manhattan plots of two repeatedly observed MTAs for stem height and storage root number per plant. **Fig S5.** Comparison of SR epidermal types based on the non-synonymous SNPs in *Sc10g012040*. **Fig. S6.** Comparison of SR epidermal types based on the non-synonymous SNPs in *Sc10g012050*. **Fig. S7.** GWAS identification of *Sc02g008280* as a candidate gene for SR endothelial color on chromosome 2. **Fig. S8.** Expression of candidate genes from GWAS analysis in different tissues and stages of storage root development.**Fig. S9.** Identification and screening of heterozygous blocks with high frequency in cultivars. **Fig. S10.** Transient overexpression and silencing of *MeTIR1* affect starch content in leaves of four cassava cultivars (F1015, R72, 4363 and Baodao9-1).**Fig. S11.** Photos of cassava leaves transformed with pCAMBIA1304 (vector control, VC1), pCAMBIA1304::MeAHL17 (OE), pTRV (vector control, VC2) or pTRV::MeAHL17 (RNAi) in four cultivars at 0 and 6 days post inoculation.

**Additional file 23.** Review history.

## Data Availability

The raw sequencing data reported in this study has been deposited in the Sequence Read Archive (SRA) under a NCBI BioProject accession (PRJNA578024) [57]. The resequencing and RNA-Seq data have been deposited under NCBI BioSample accessions (SRR10389908-SRR10390244 and SRR10480846-SRR10480905). The cassava reference genome used in this study has been deposited in the NCBI-SRA [26]. The published resequencing data used in this study is available from NCBI-SRA [6] and CassavaBase [24]. The known QTLs used in this study is available from NCBI-SRA [29, 30].
