# Supplementary material for: Resequencing of 388 cassava accessions identifies valuable loci and selection for variation in heterozygosity
Source: Genome Biol. 2021 Nov 16;22:316. doi: 10.1186/s13059-021-02524-7 (PMC8594203; doi:10.1186/s13059-021-02524-7)
Supplement: Supplementary file 22 — Additional file 22: Fig. S1-S11. Fig. S1. Phylogeny of 388 cassava accessions generated using the neighbor-joining tree method with genome-wide SNPs. Fig S2. Linkage disequilibrium (LD) decay for different groups. Fig. S3. Manhattan plots for GWAS analysis of cassava agronomic traits. Fig. S4. Manhattan plots of two repeatedly observed MTAs for stem height and storage root number per plant. Fig S5. Comparison of SR epidermal types based on the non-synonymous SNPs in Sc10g012040. Fig. S6. Comparison of SR epidermal types based on the non-synonymous SNPs in Sc10g012050. Fig. S7. GWAS identification of Sc02g008280 as a candidate gene for SR endothelial color on chromosome 2. Fig. S8. Expression of candidate genes from GWAS analysis in different tissues and stages of storage root development.Fig. S9. Identification and screening of heterozygous blocks with high frequency in cultivars. Fig. S10. Transient overexpression and silencing of MeTIR1 affect starch content in leaves of four cassava cultivars (F1015, R72, 4363 and Baodao9-1).Fig. S11. Photos of cassava leaves transformed with pCAMBIA1304 (vector control, VC1), pCAMBIA1304::MeAHL17 (OE), pTRV (vector control, VC2) or pTRV::MeAHL17 (RNAi) in four cultivars at 0 and 6 days post inoculation. [file 13059_2021_2524_MOESM22_ESM.docx]

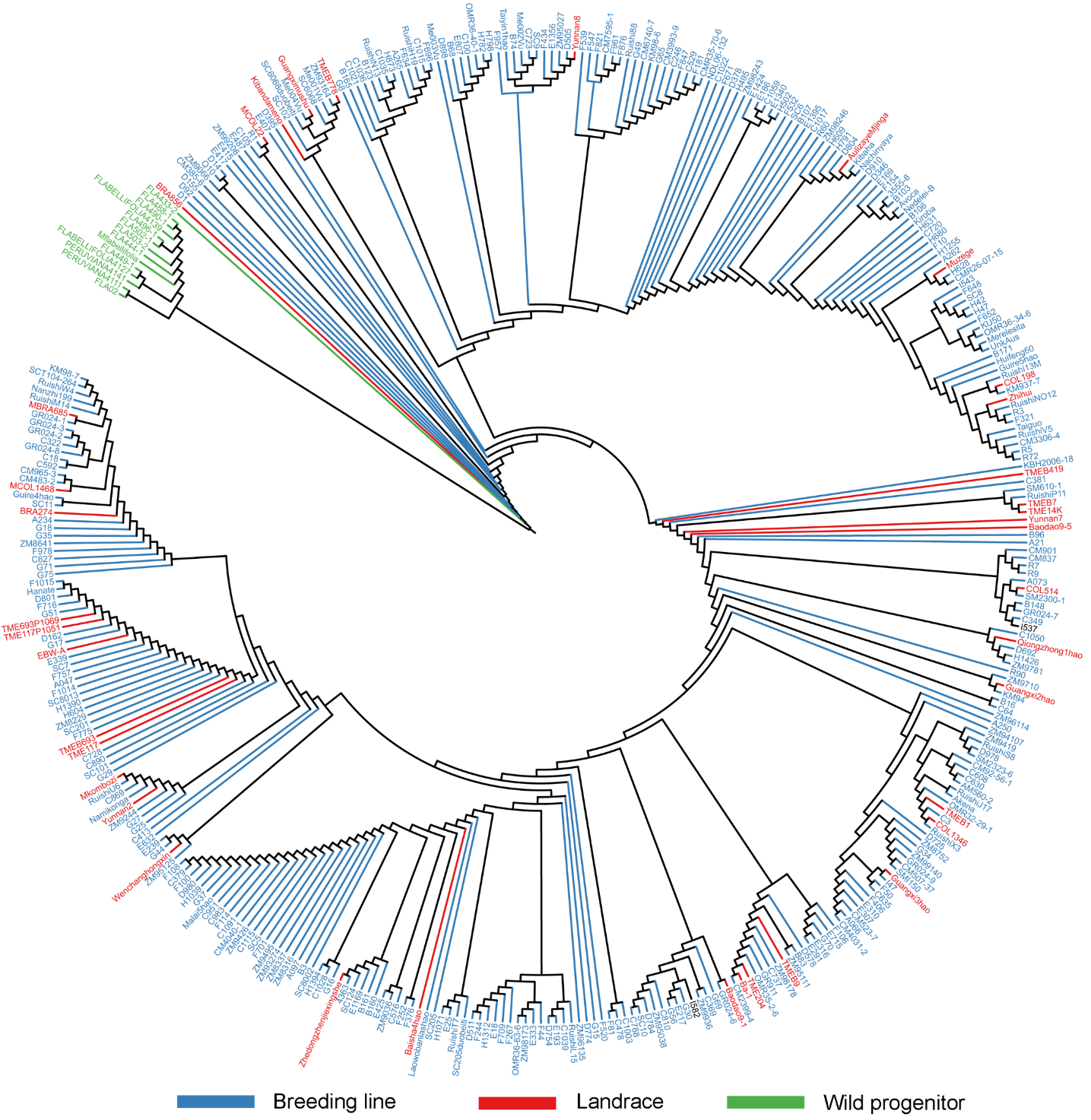


**Fig S1. Phylogeny of 388 cassava accessions generated using the neighbor-joining tree method with genome-wide SNPs.**

**
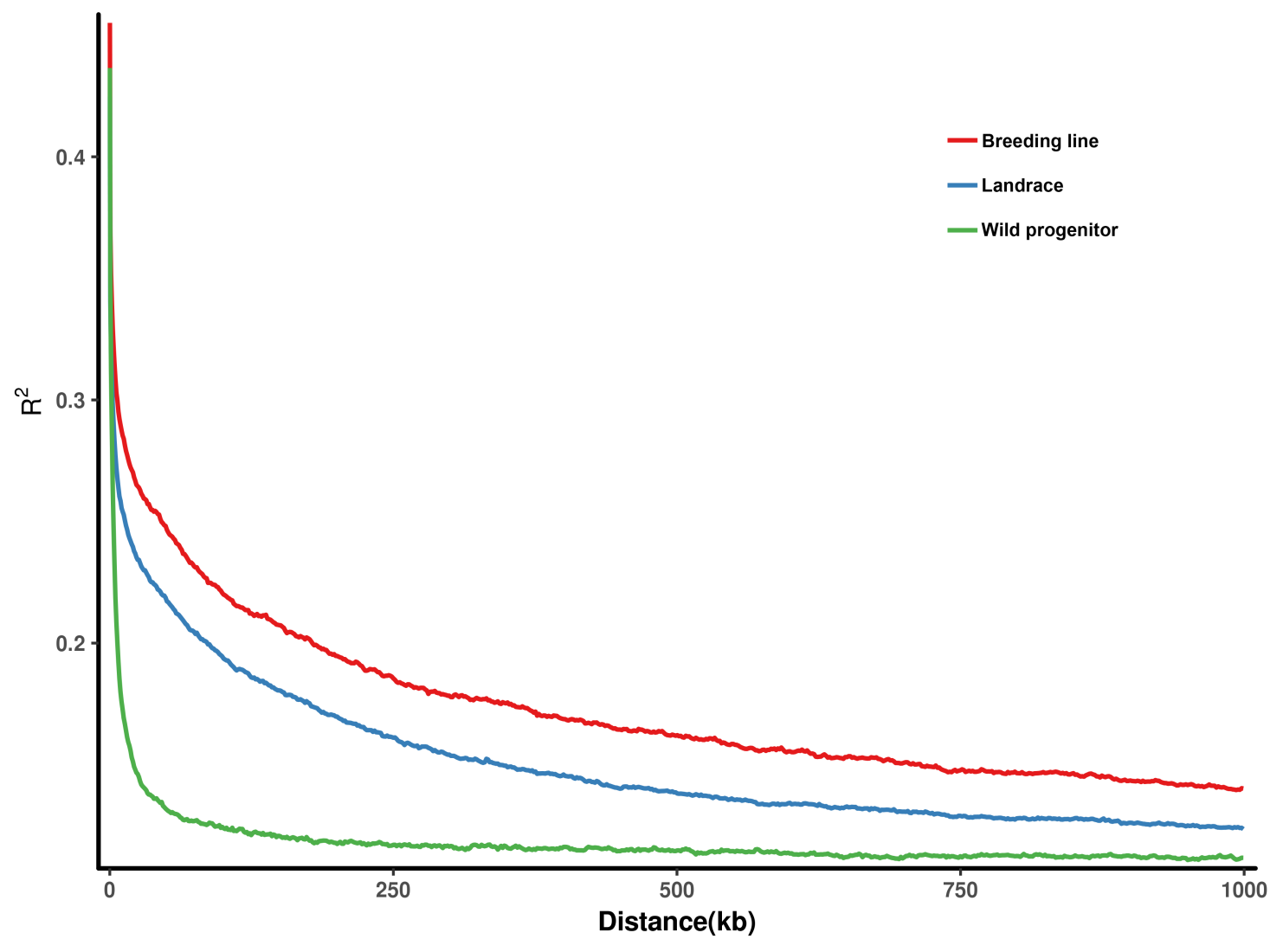
**

**Fig S2. Linkage disequilibrium (LD) decay for different groups.** Fourteen wild progenitors, 14 randomly selected landraces, and 14 randomly selected breeding lines were used to calculate LD.


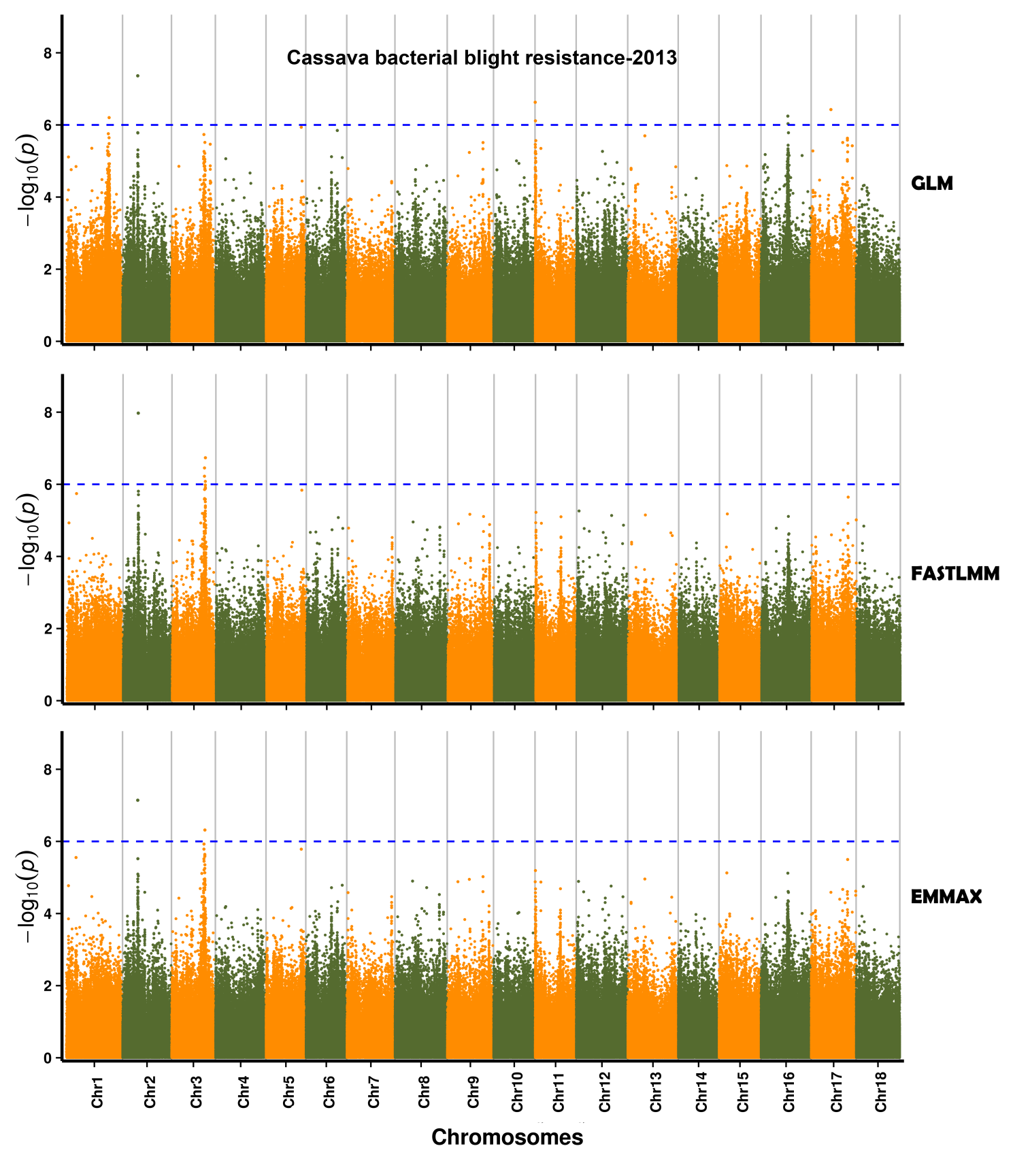


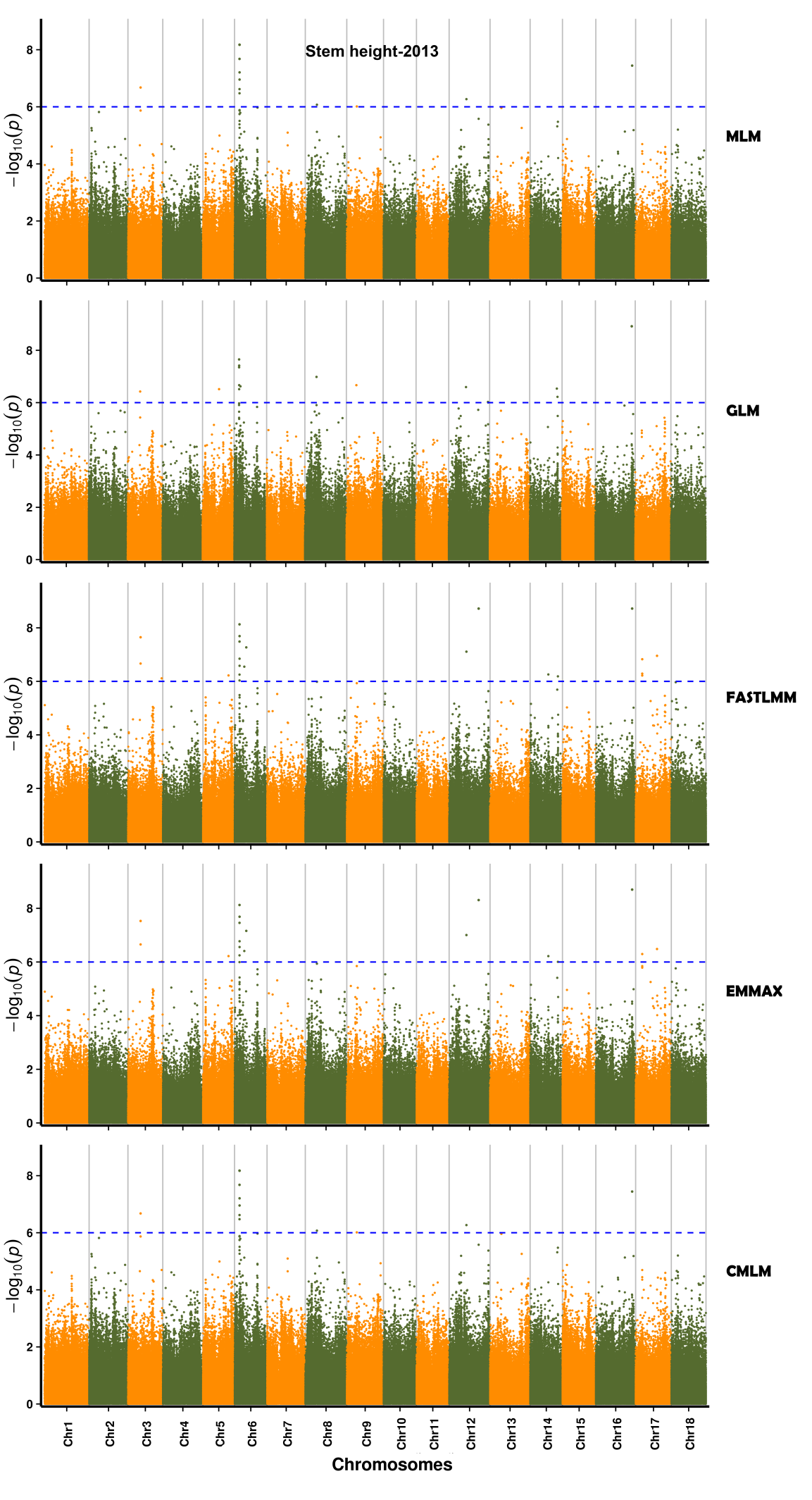


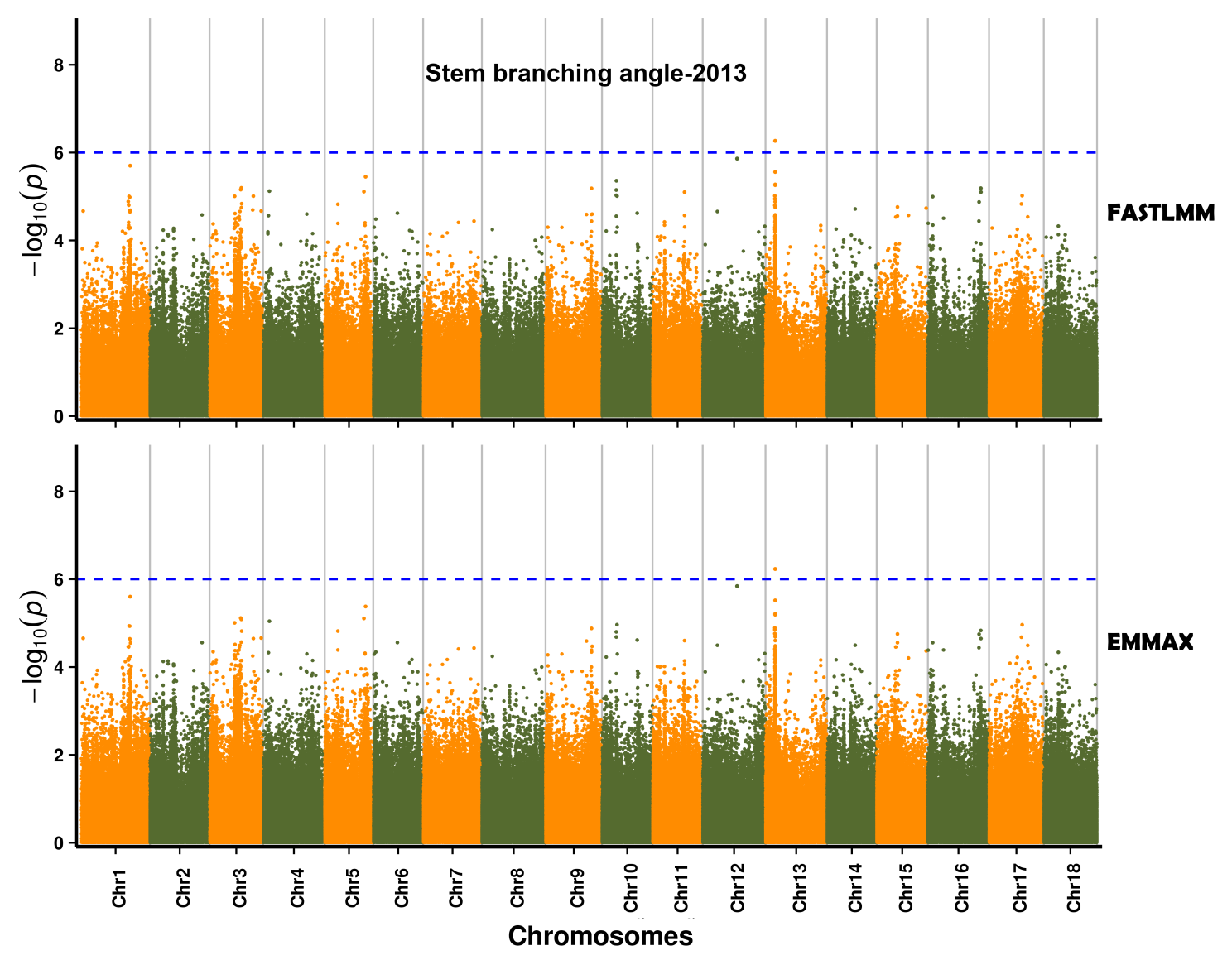


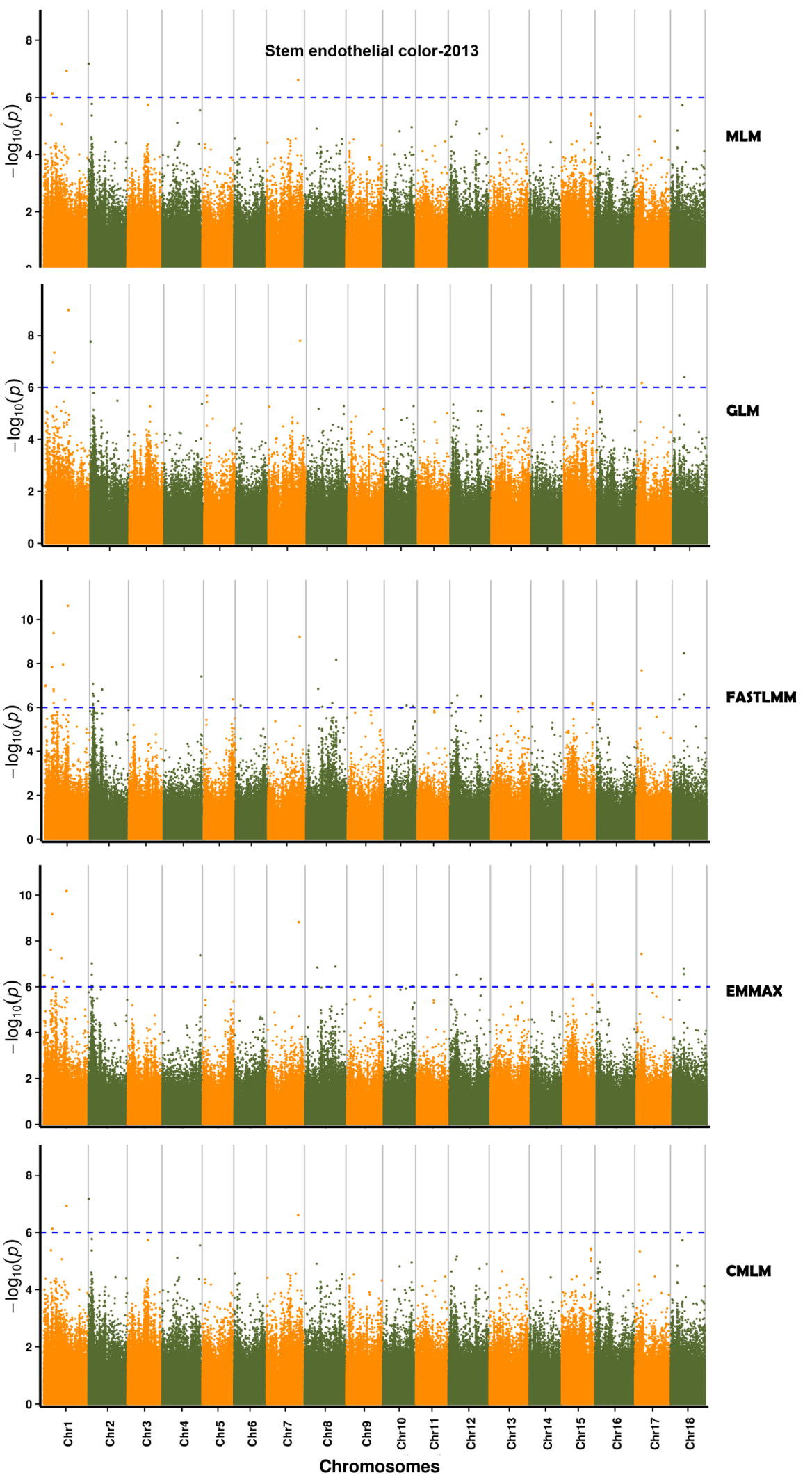


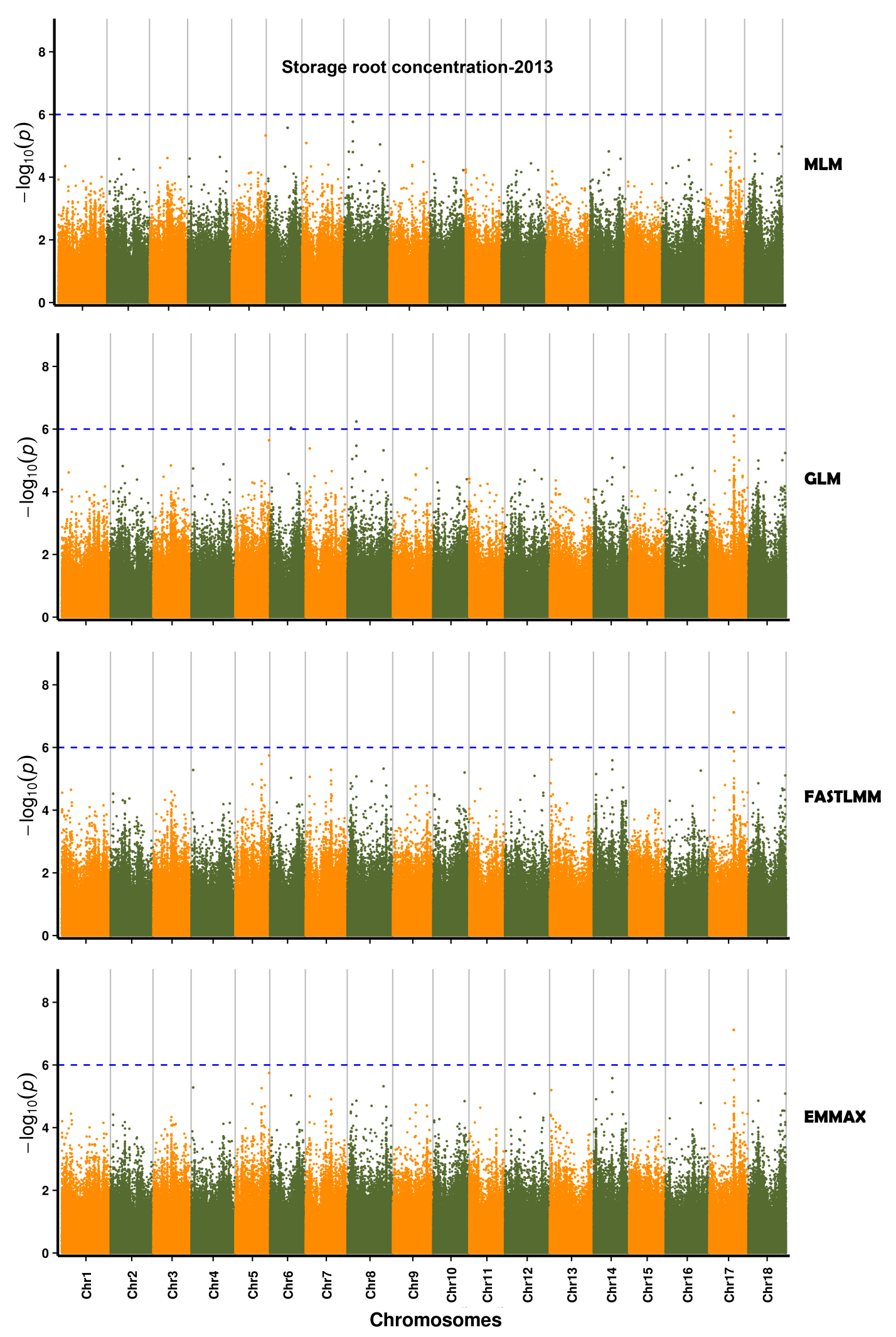


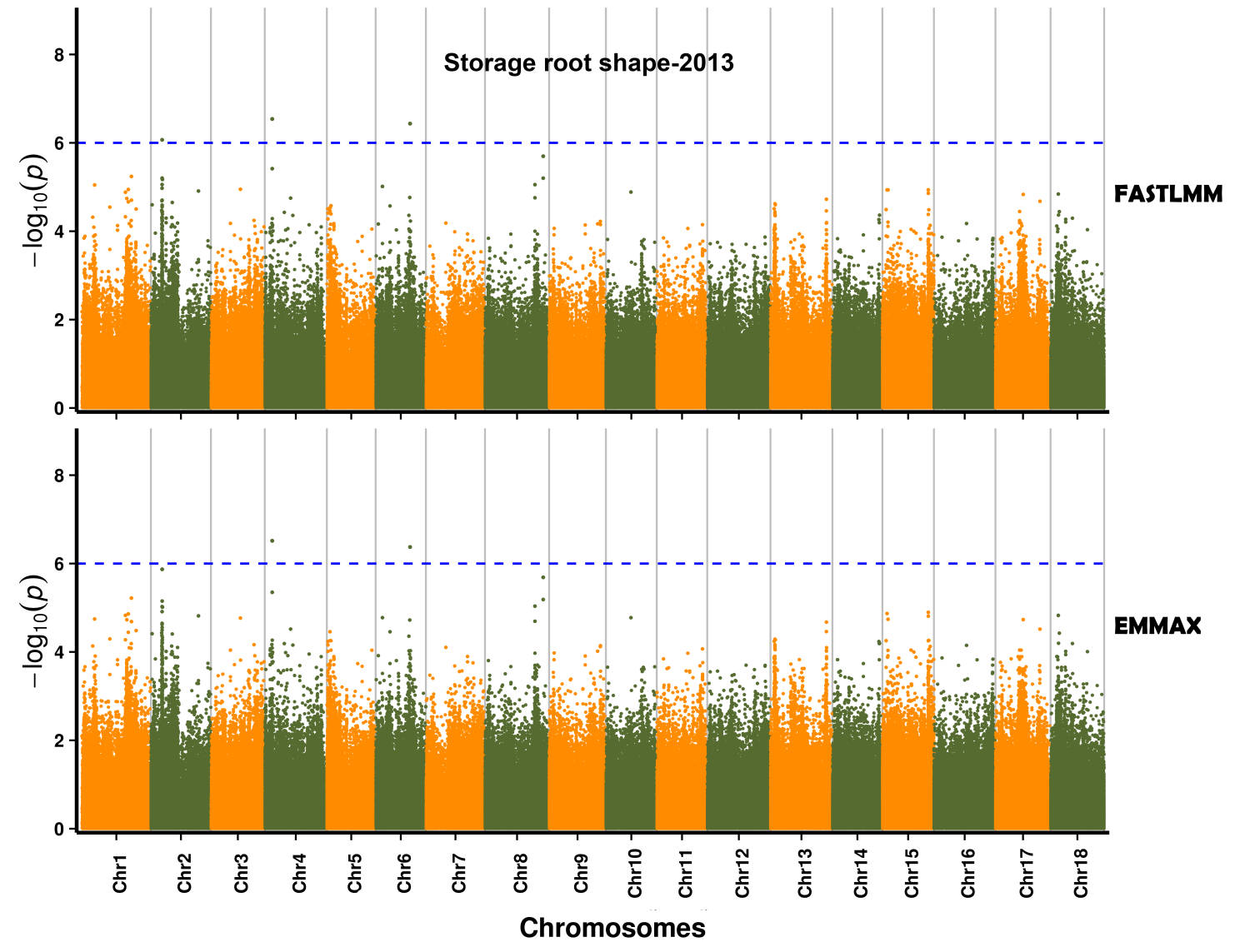


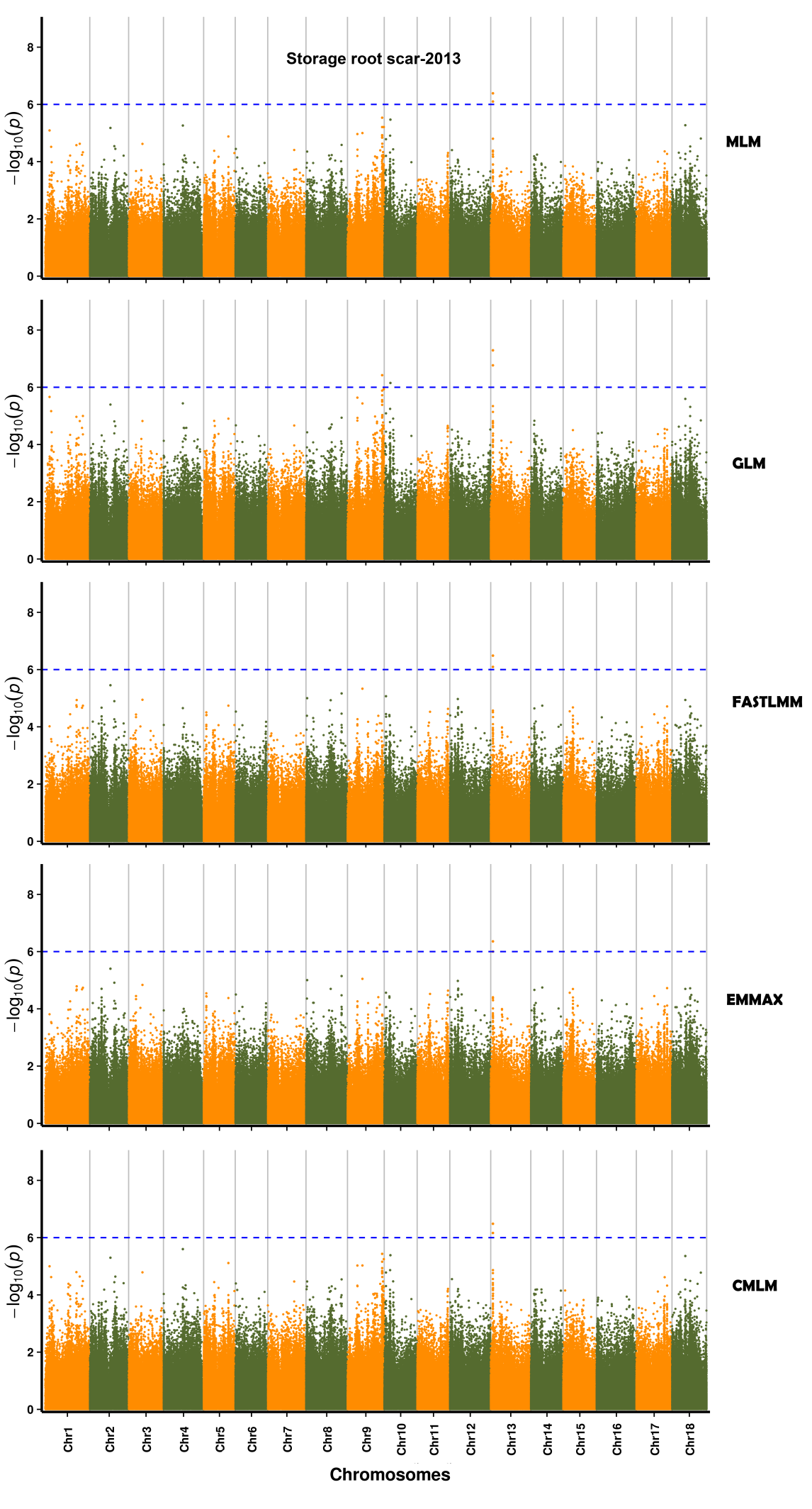


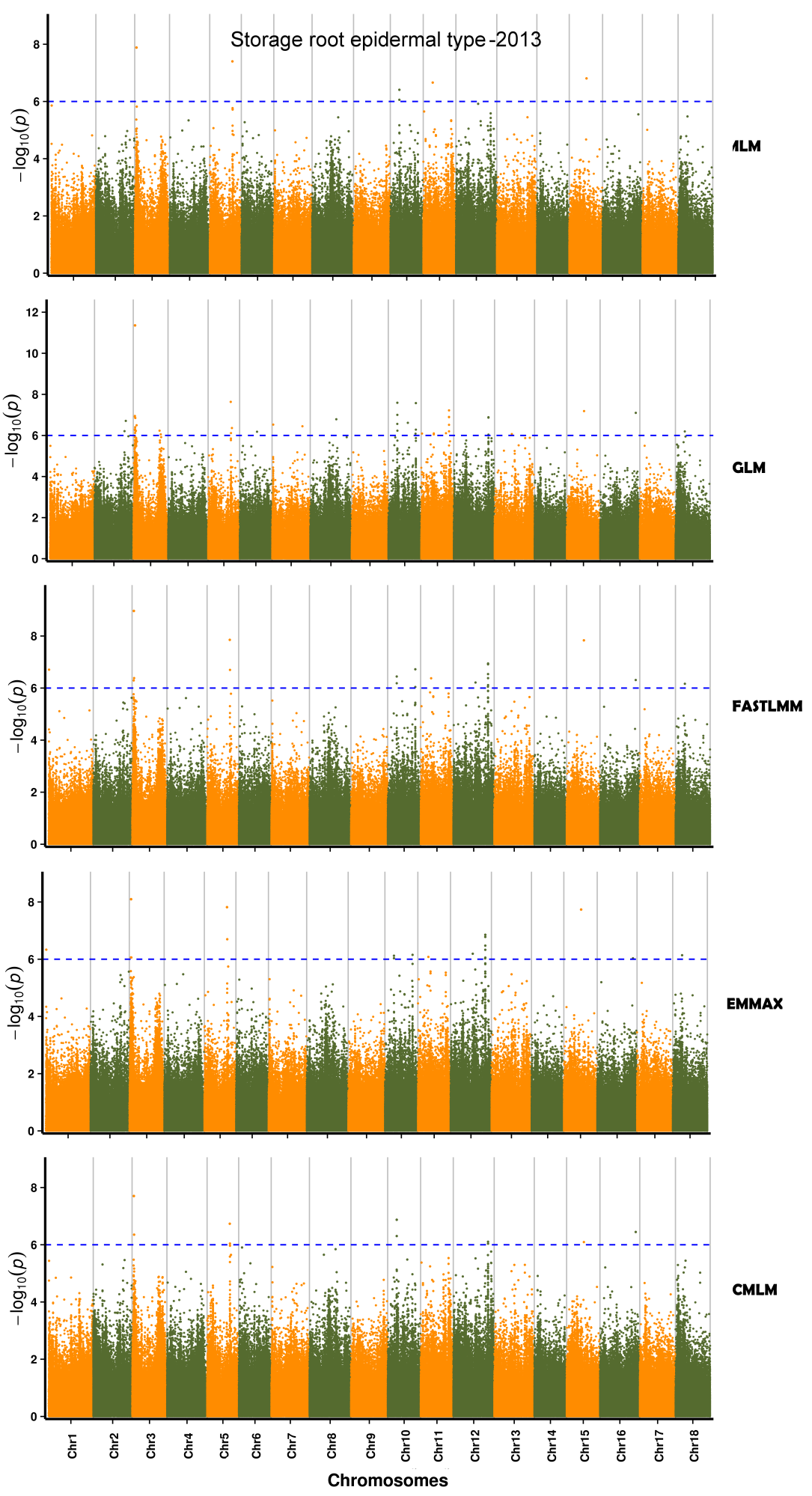


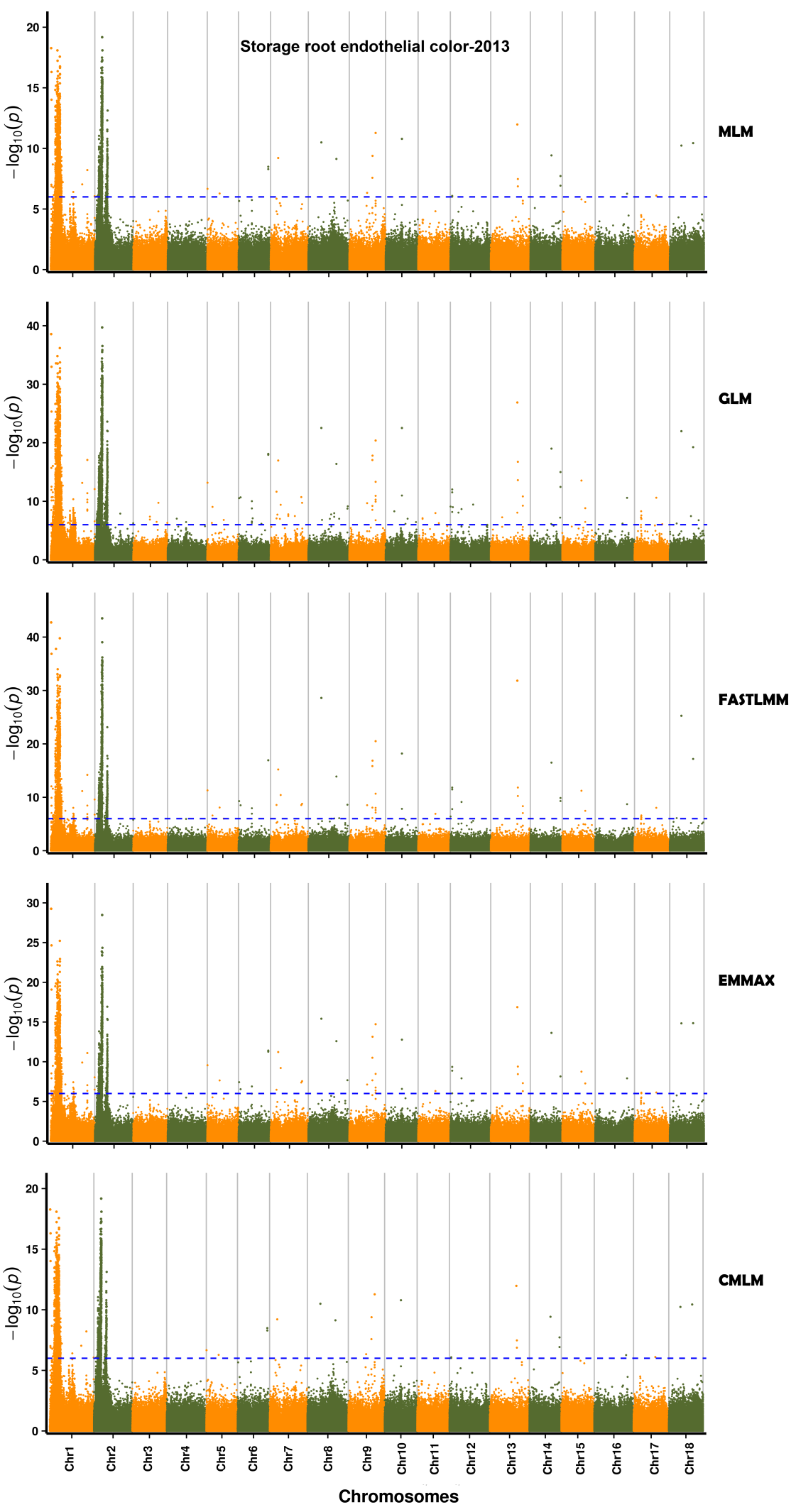


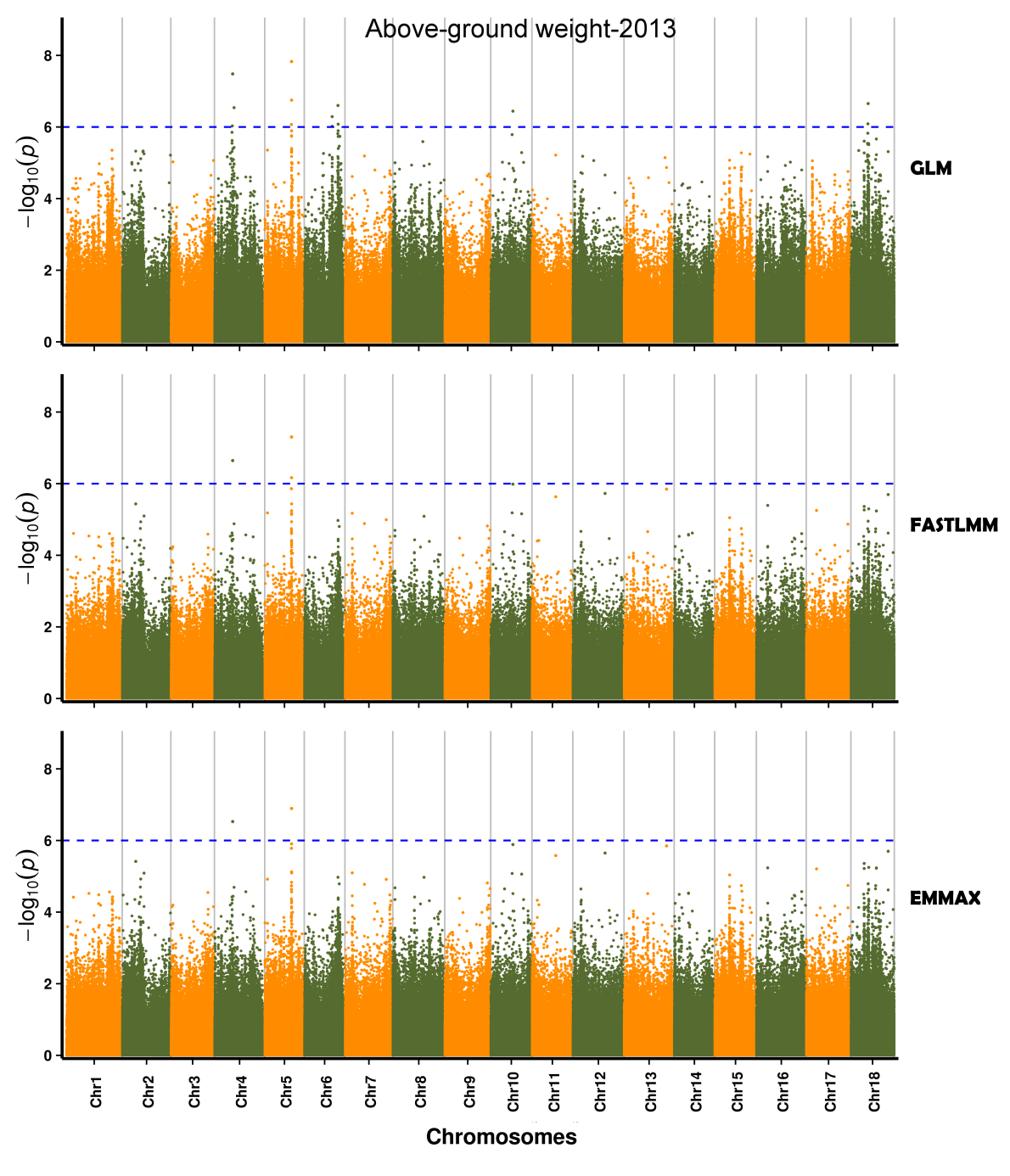


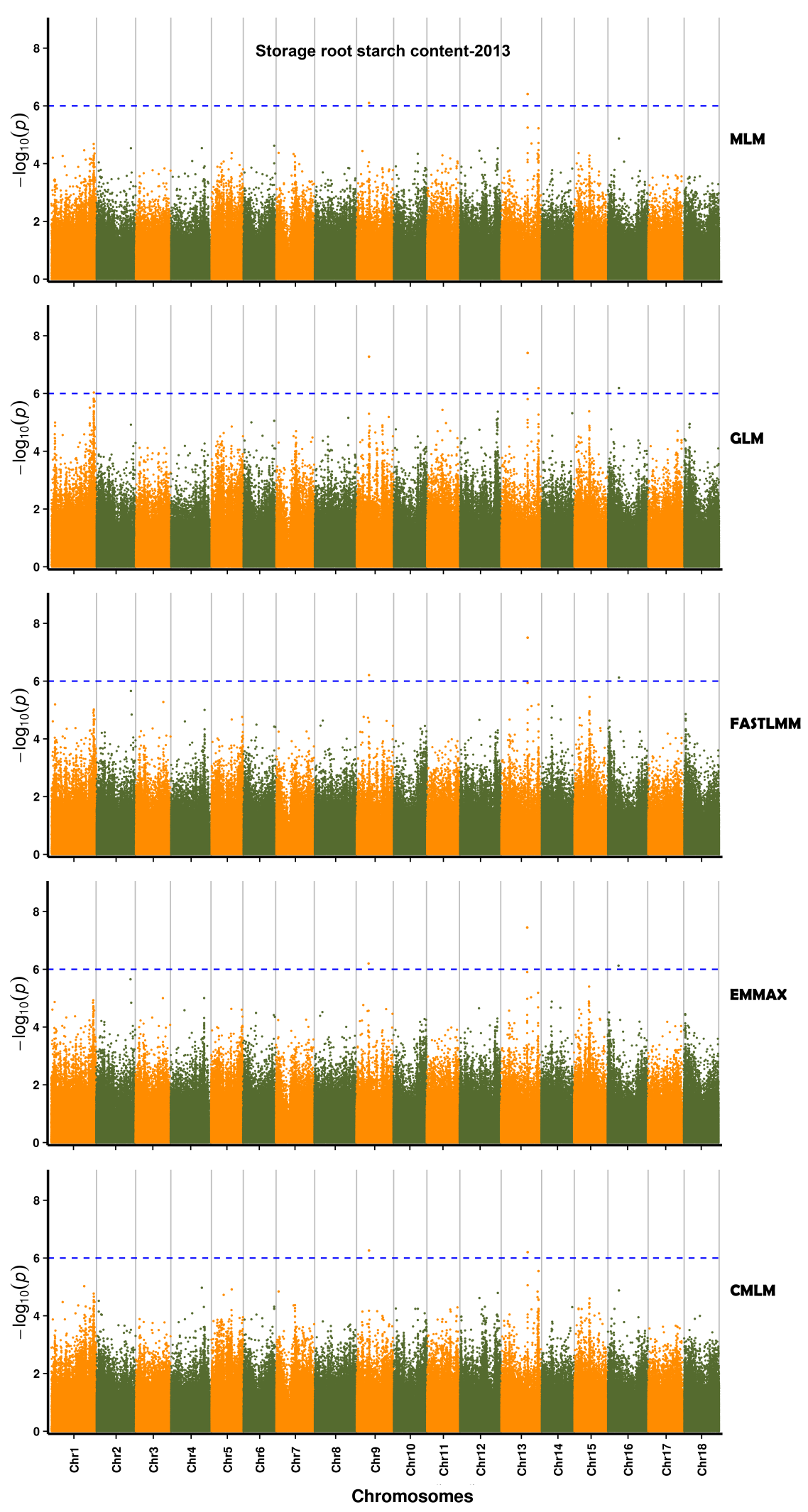


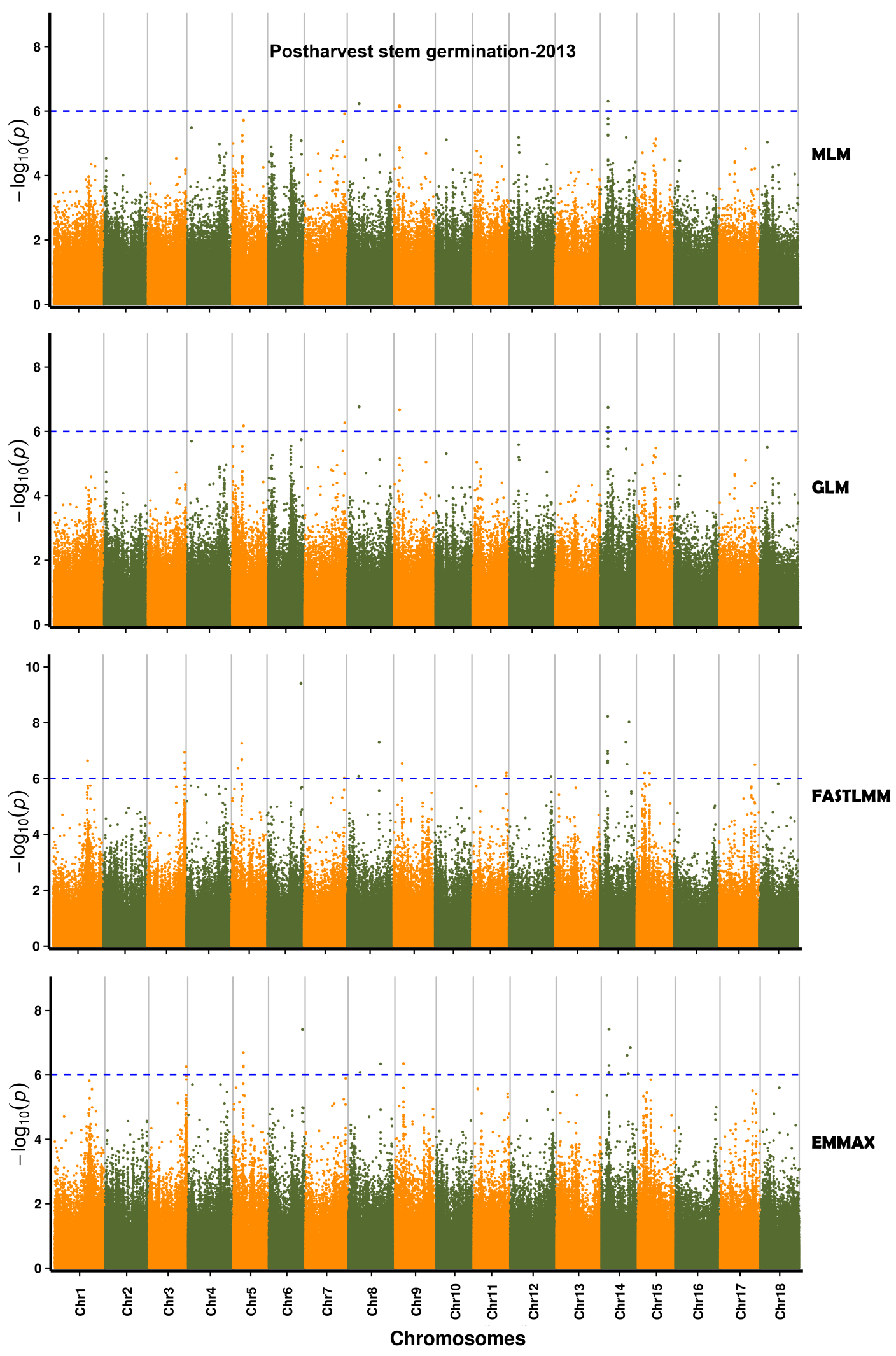


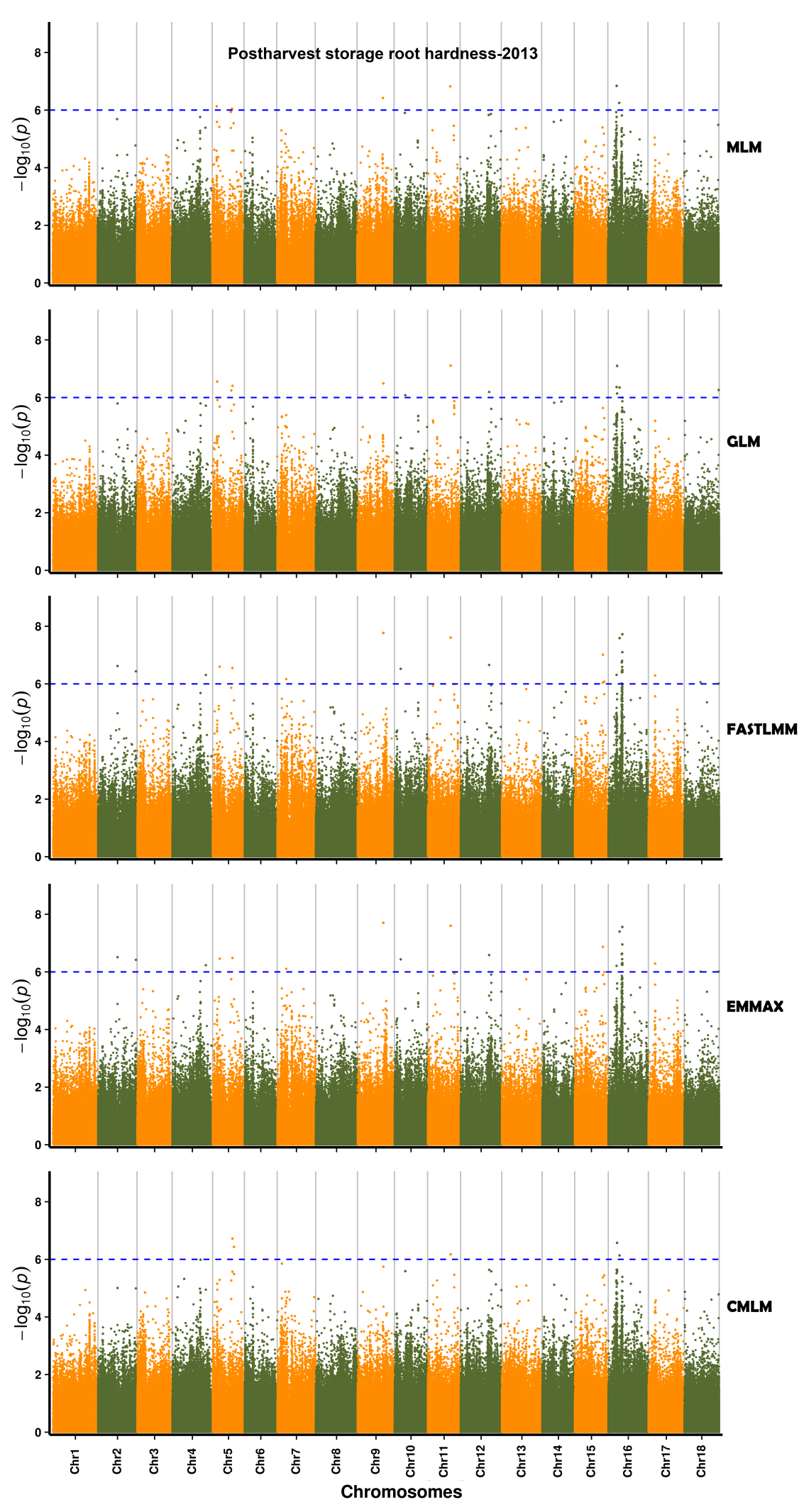


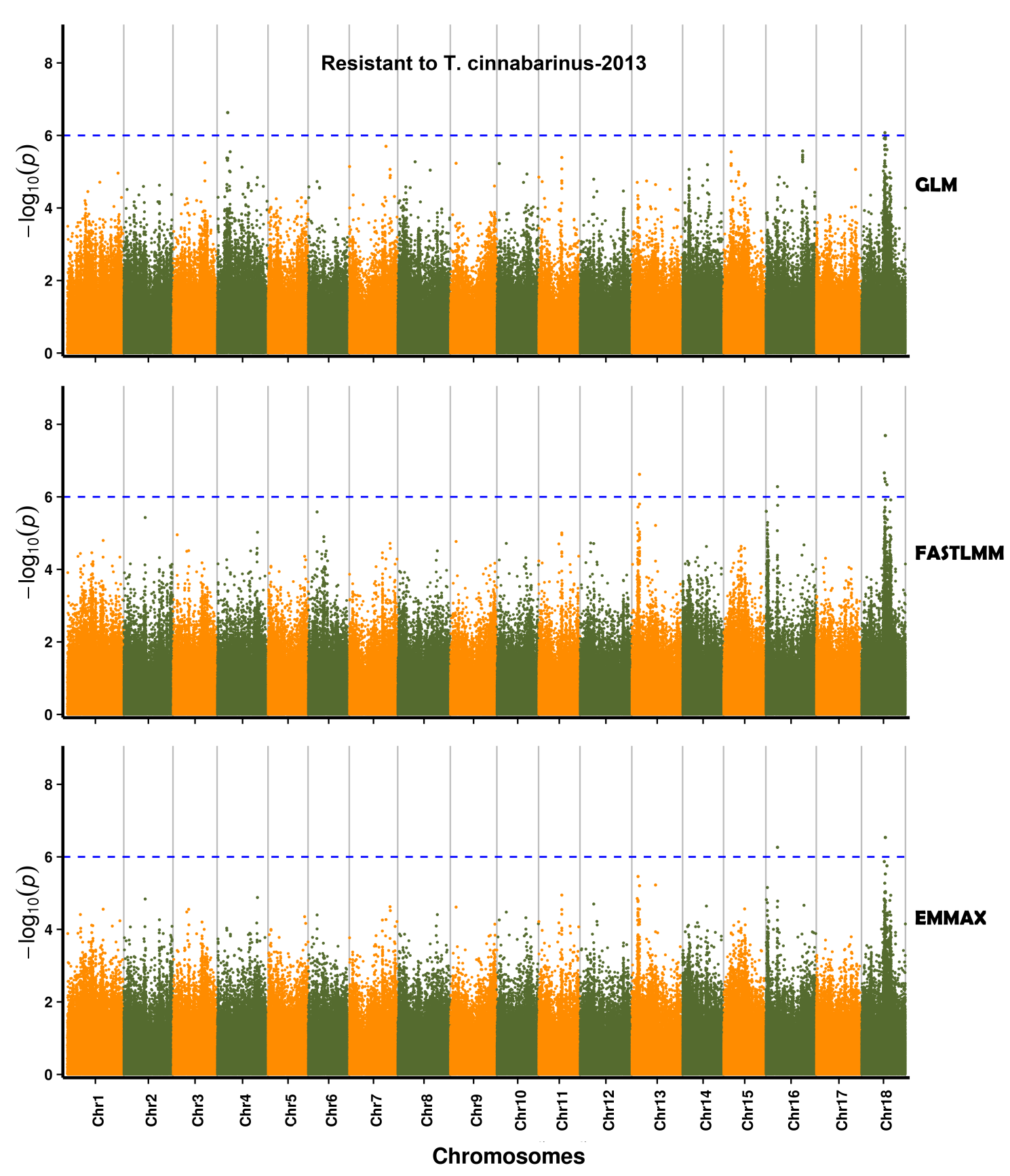


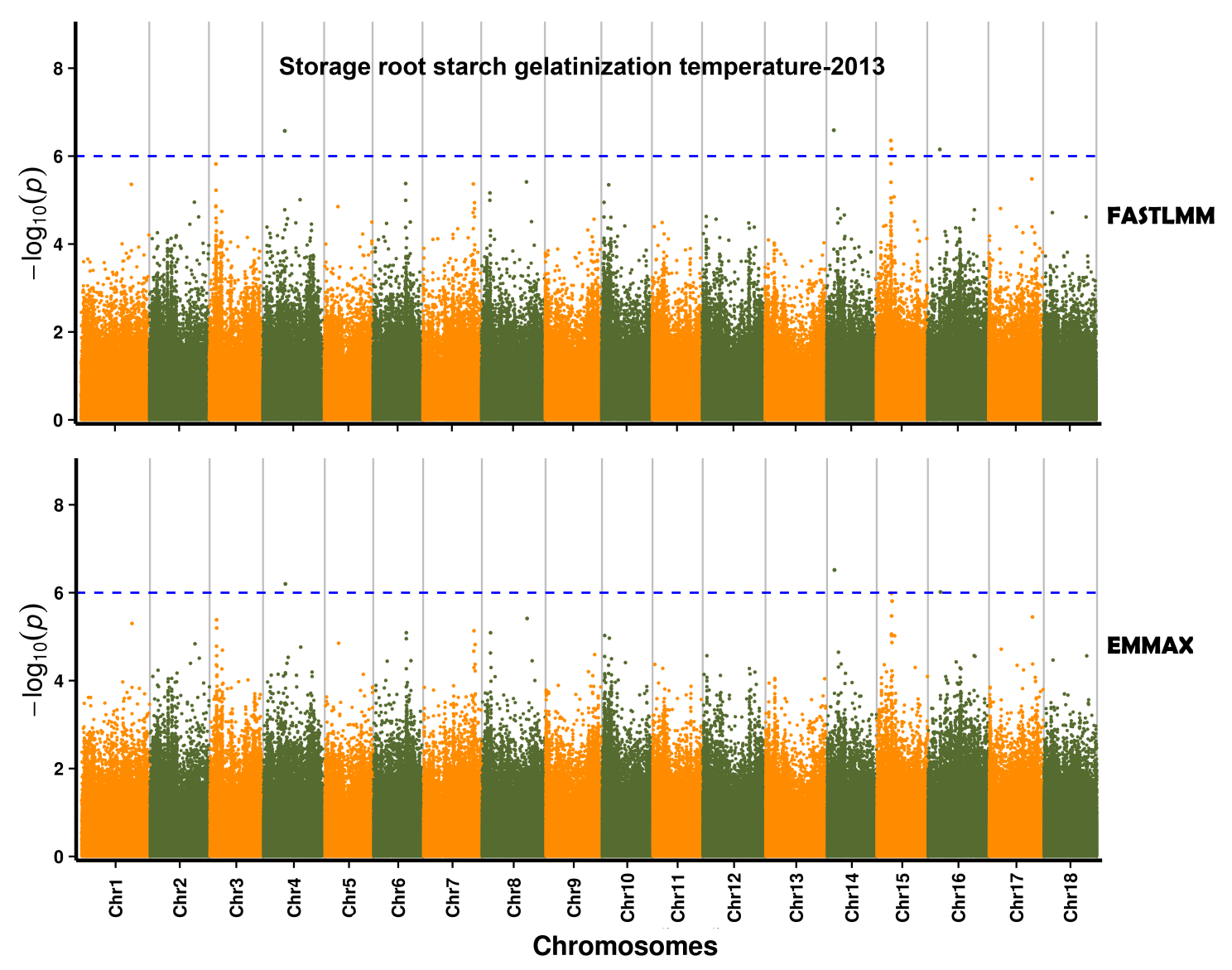


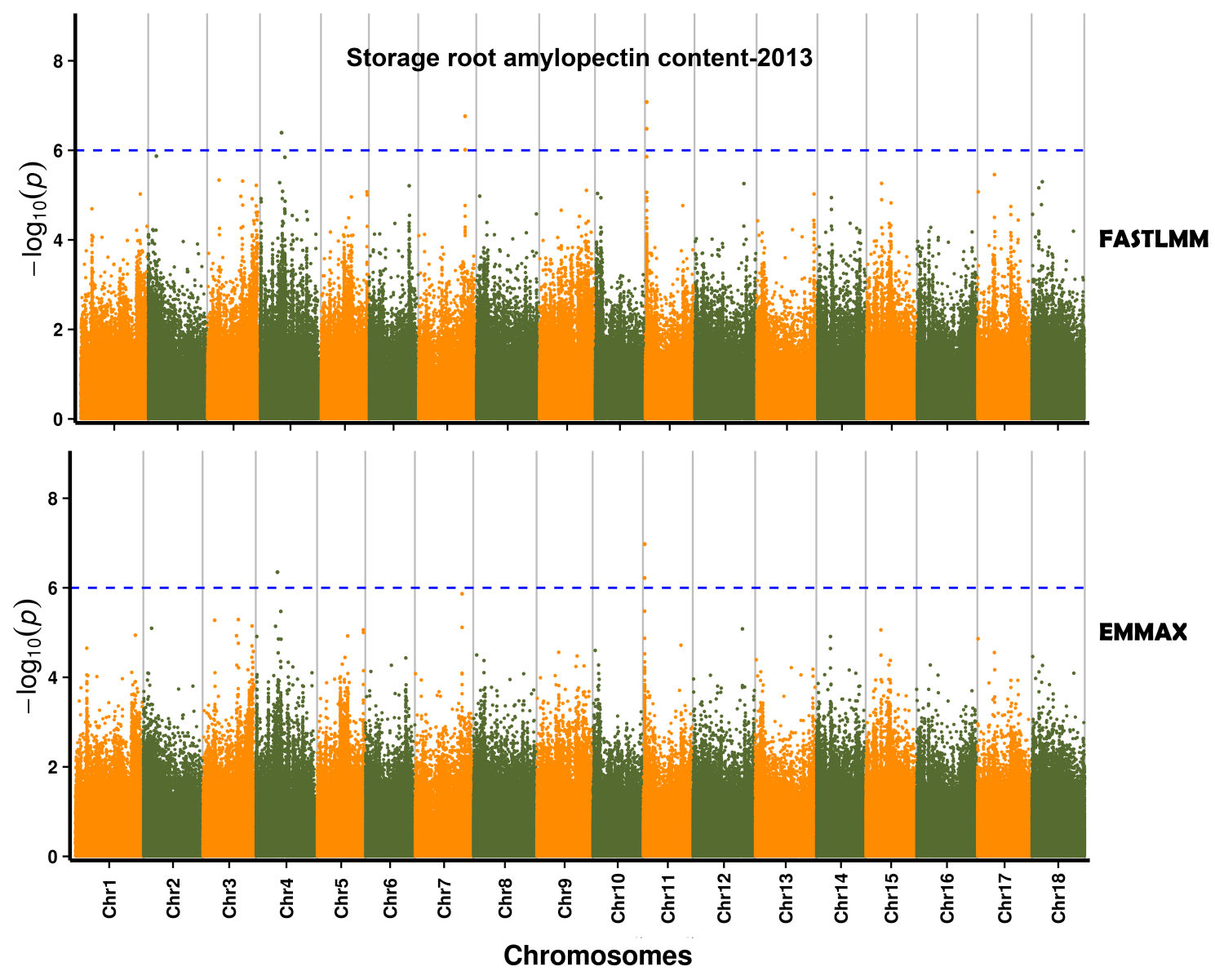


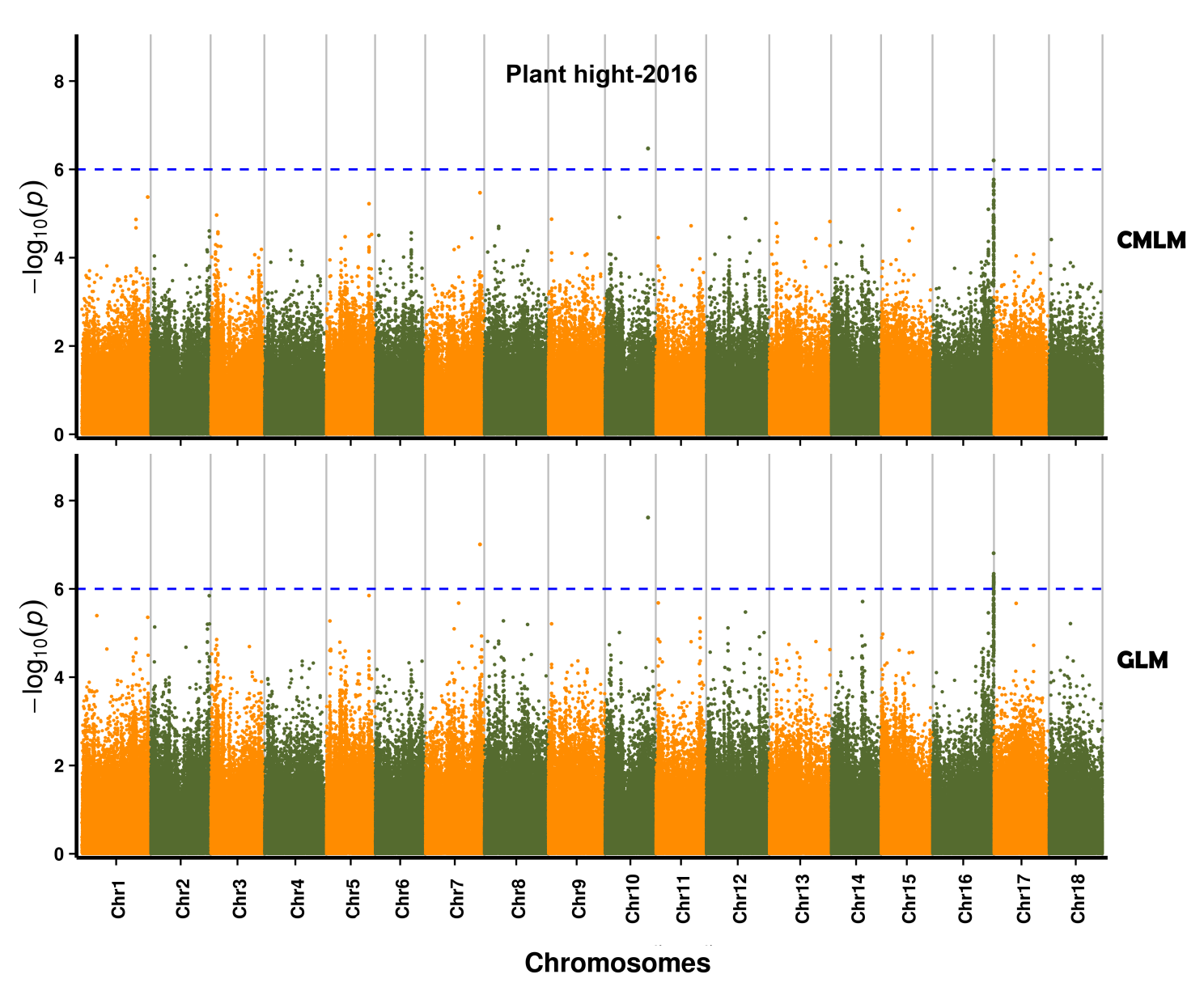


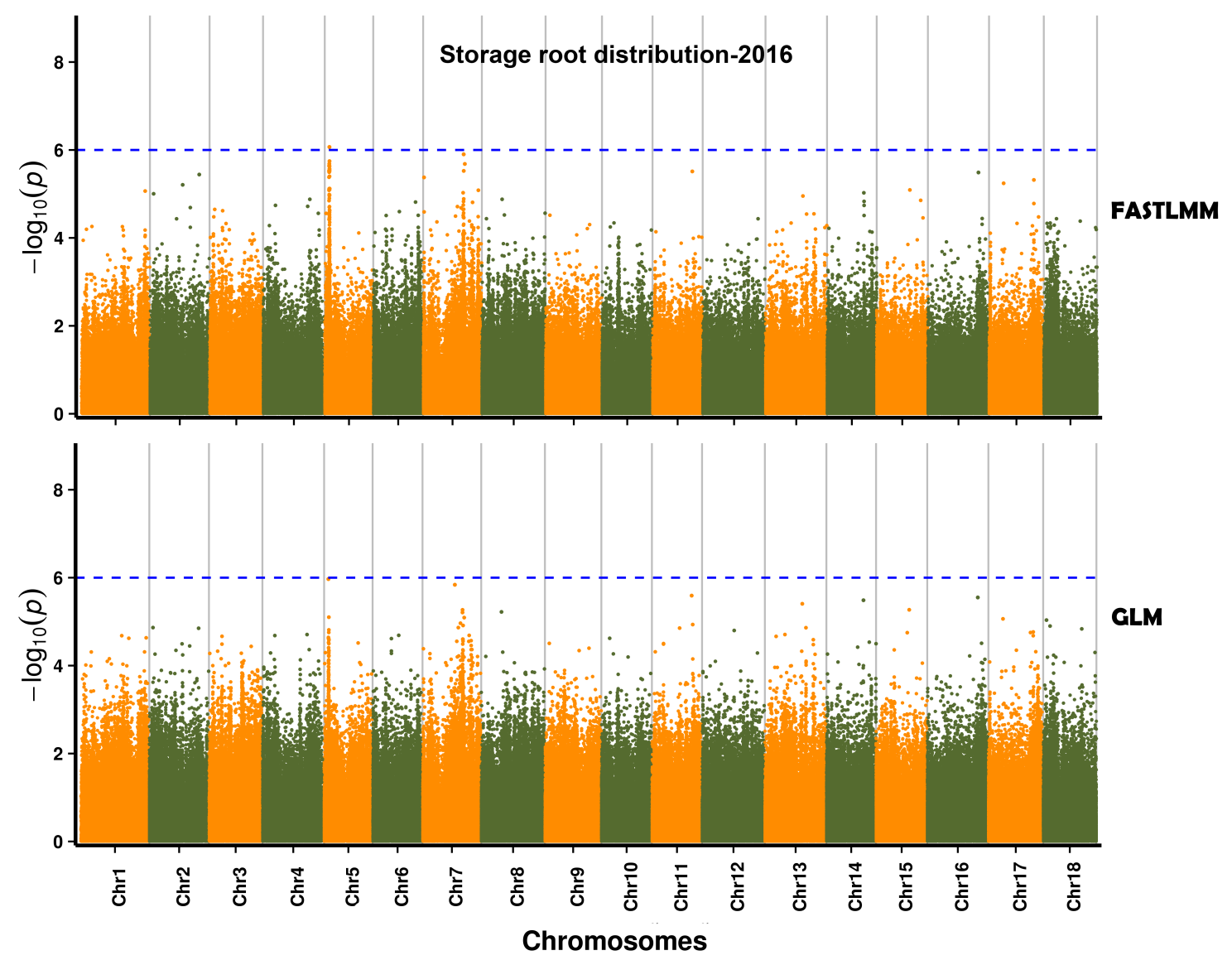


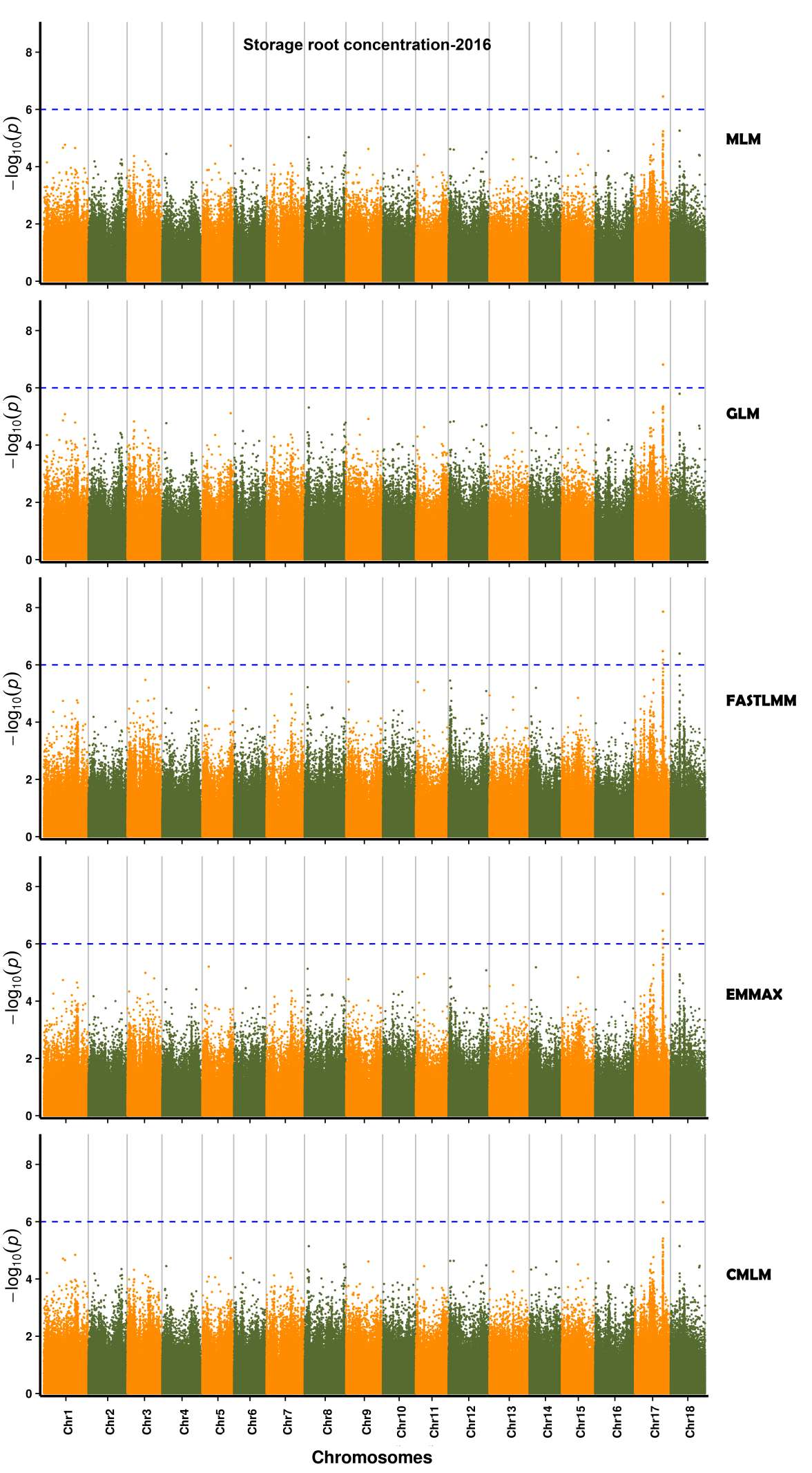


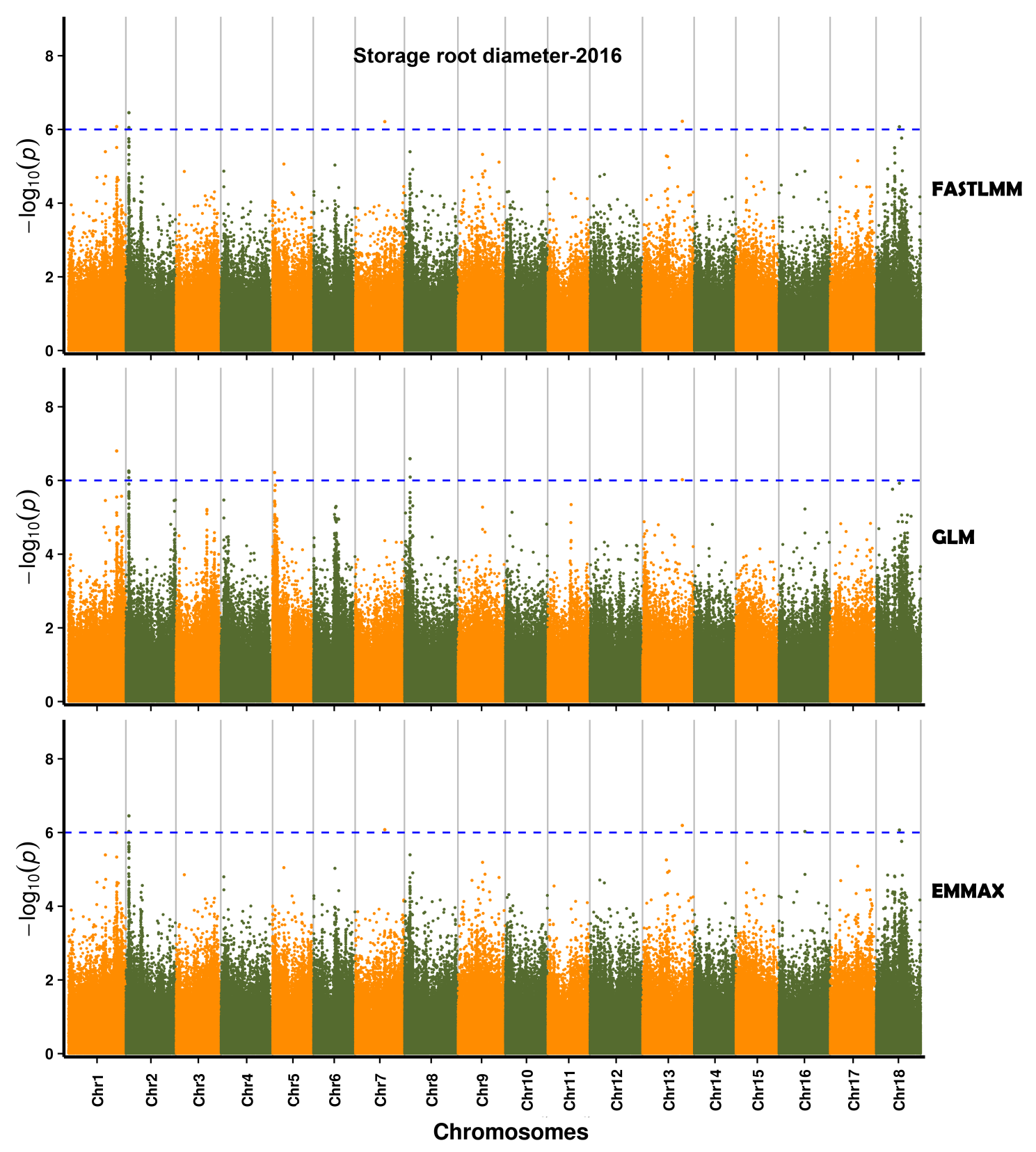


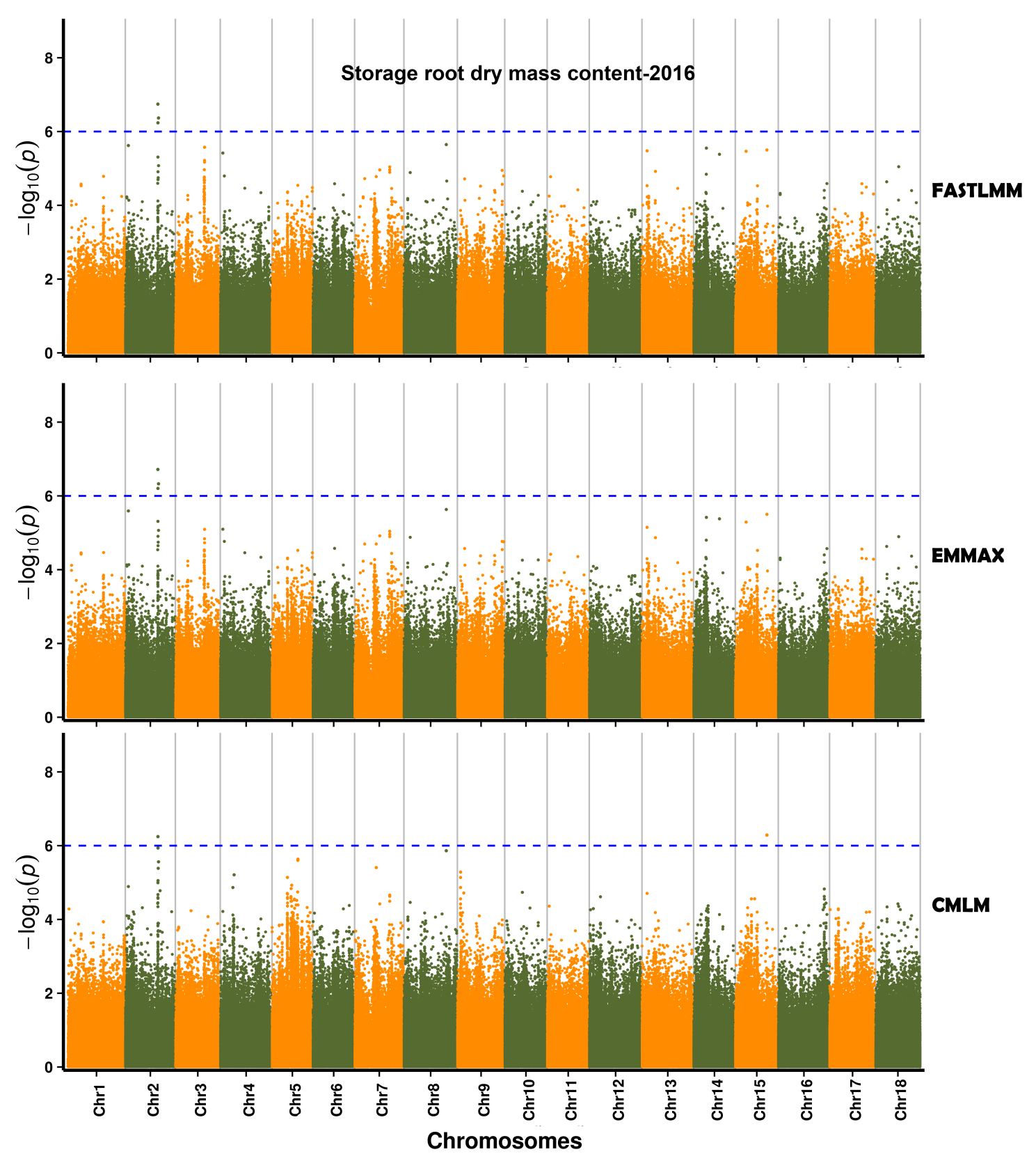


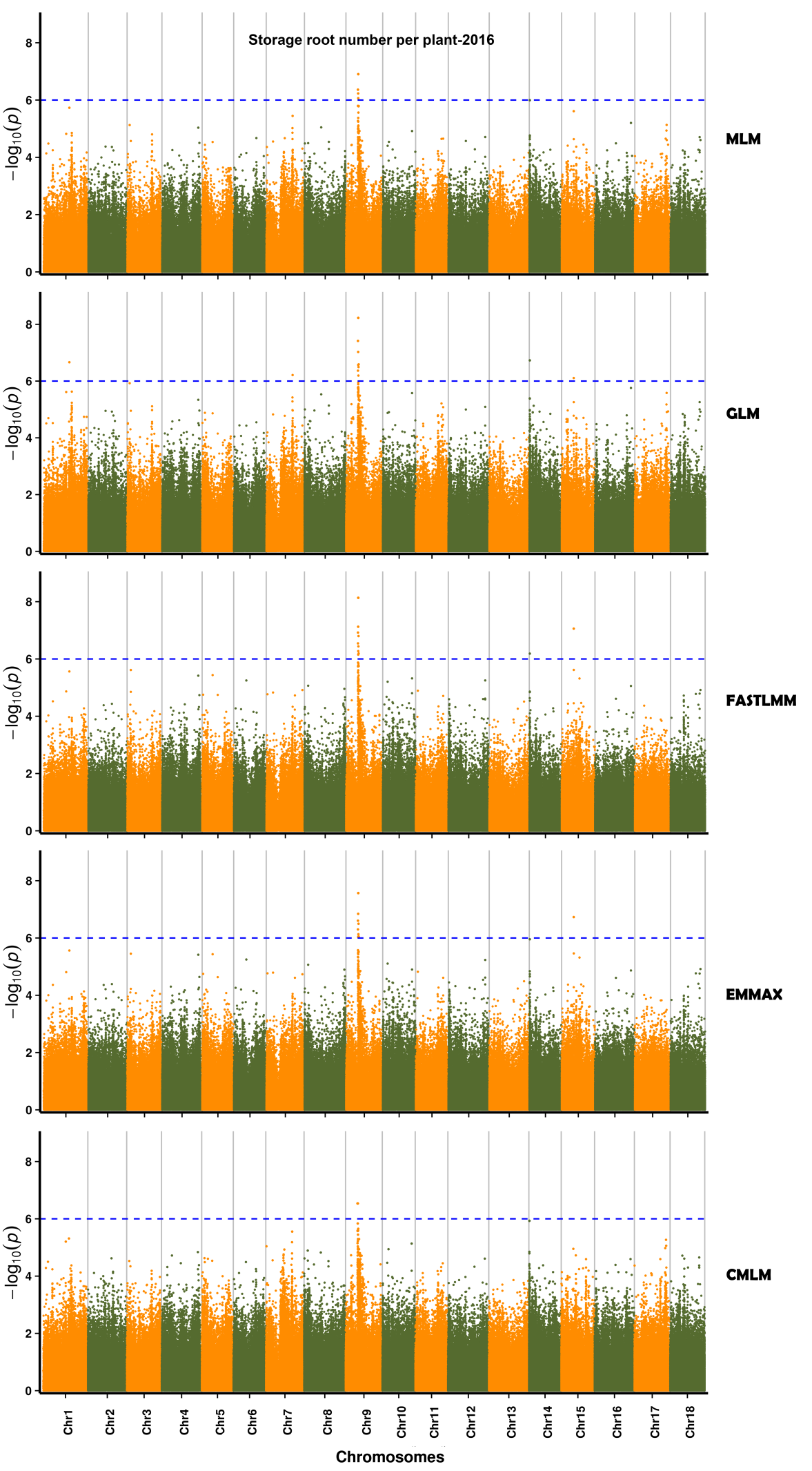


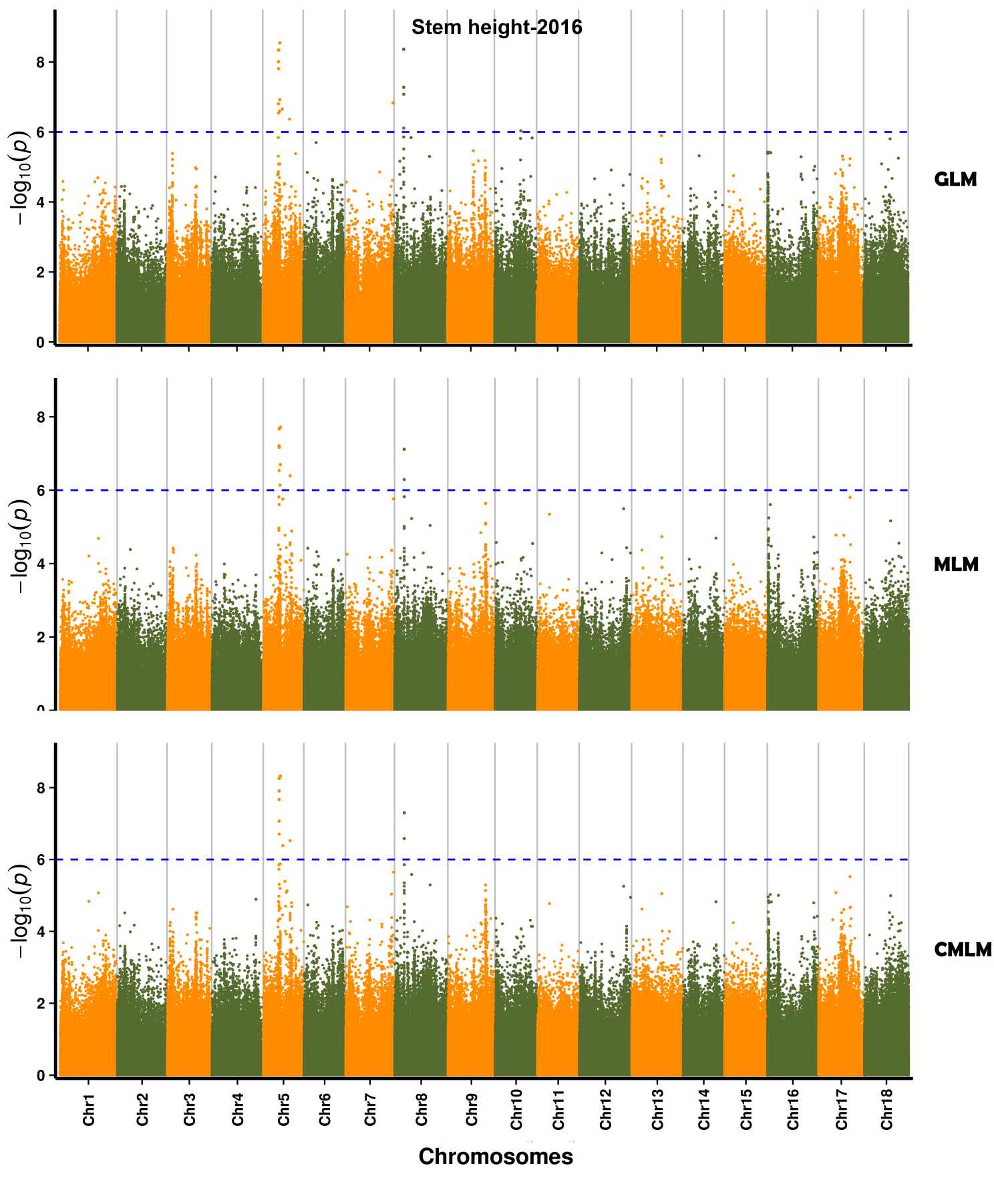


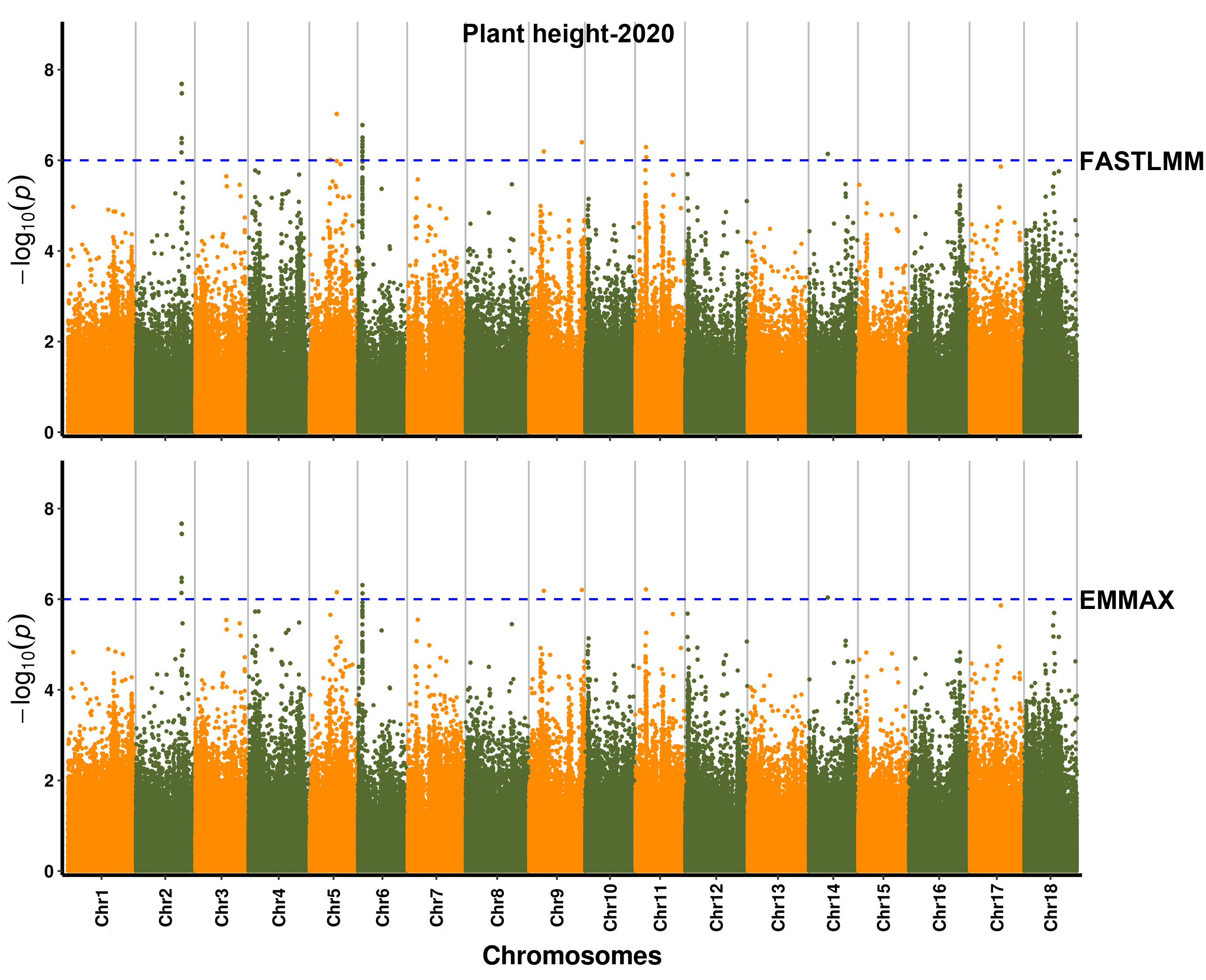


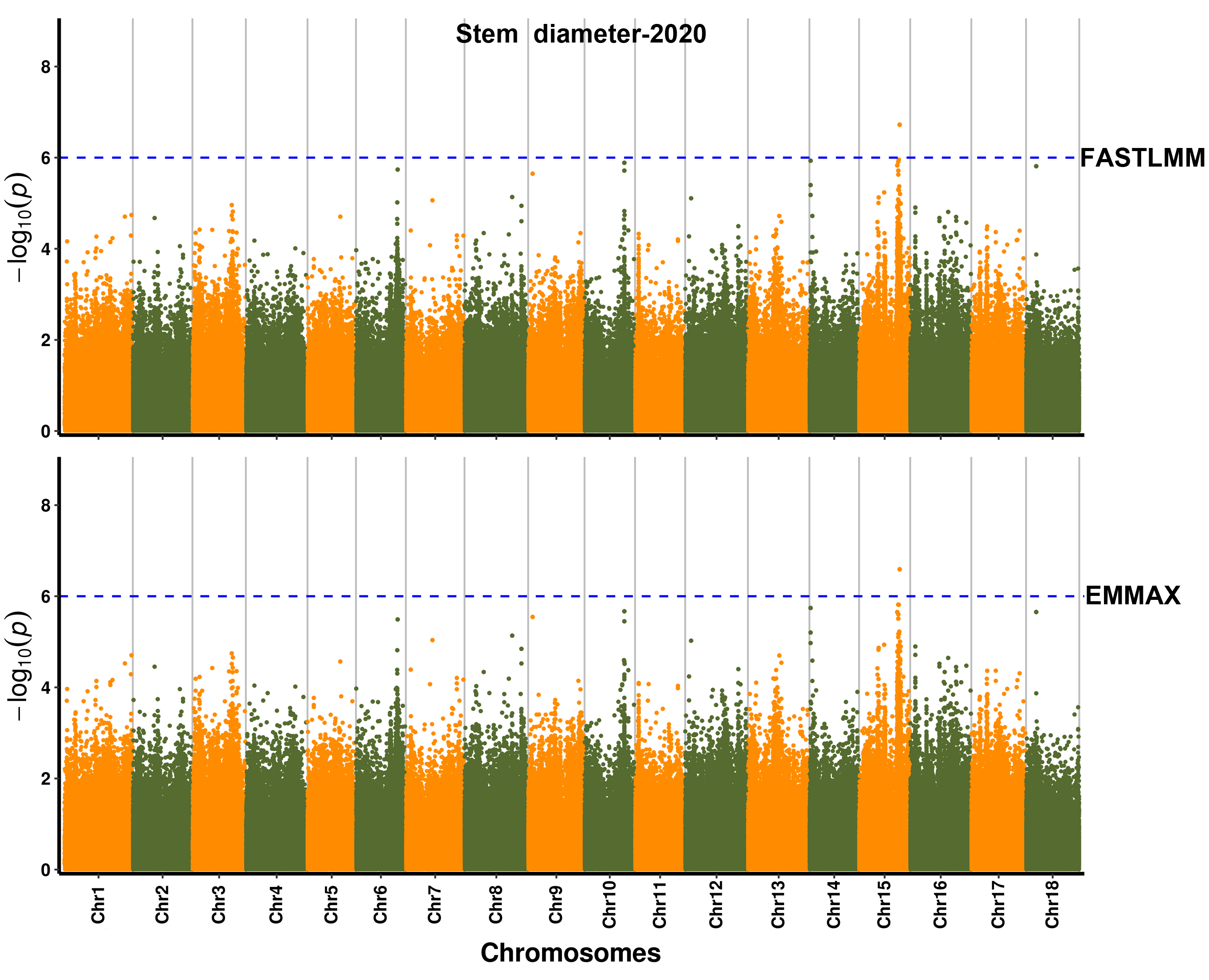


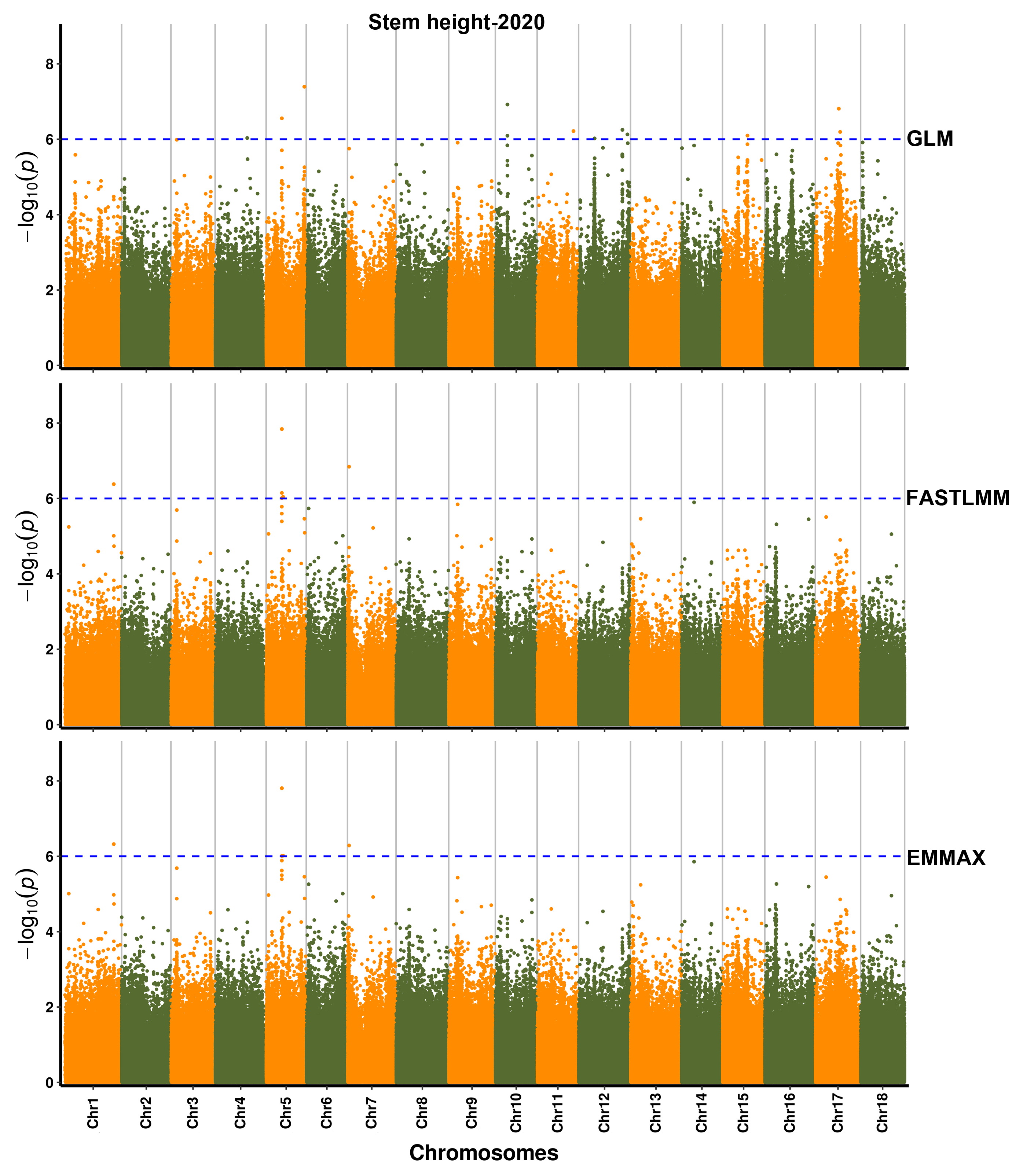


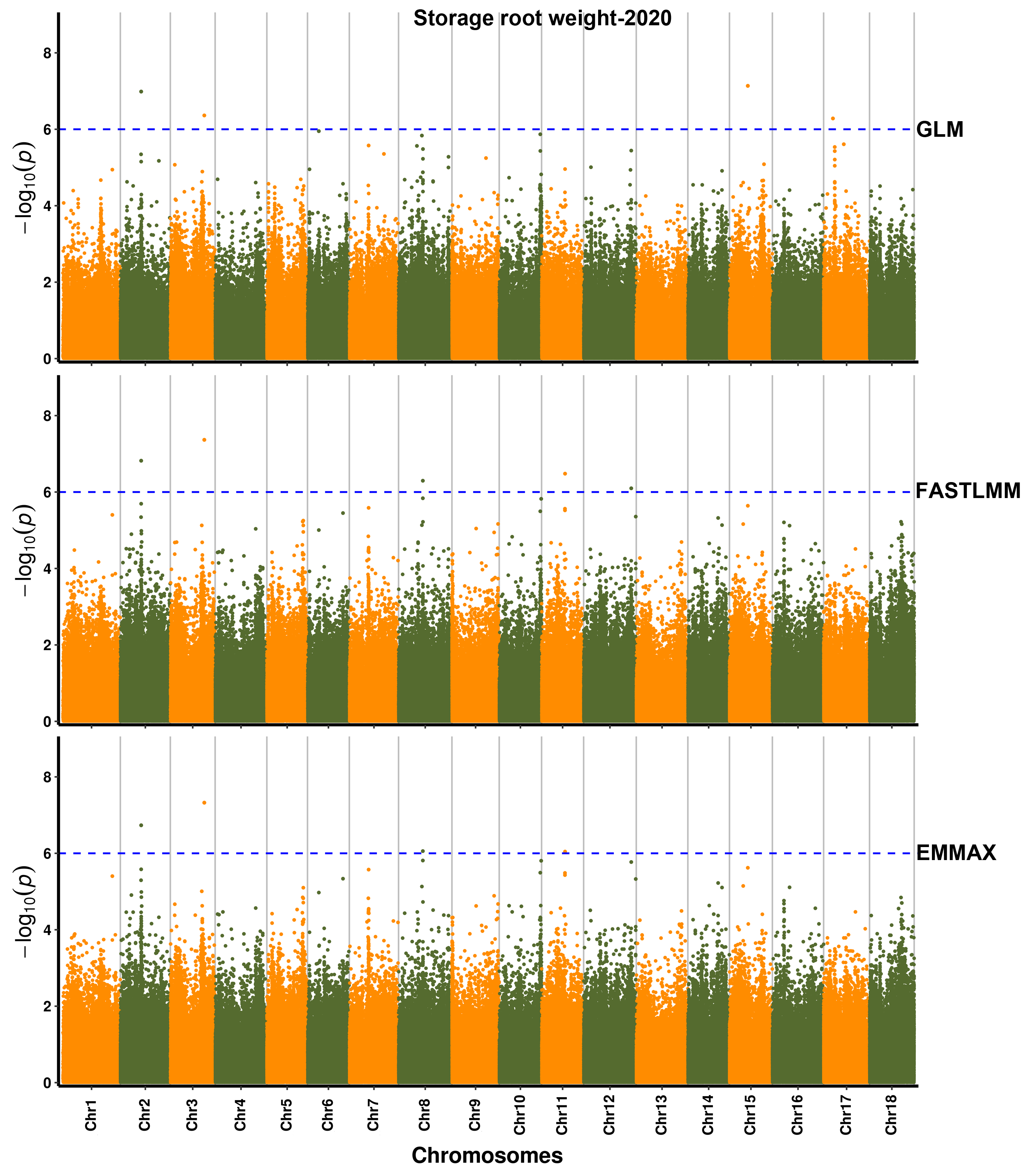


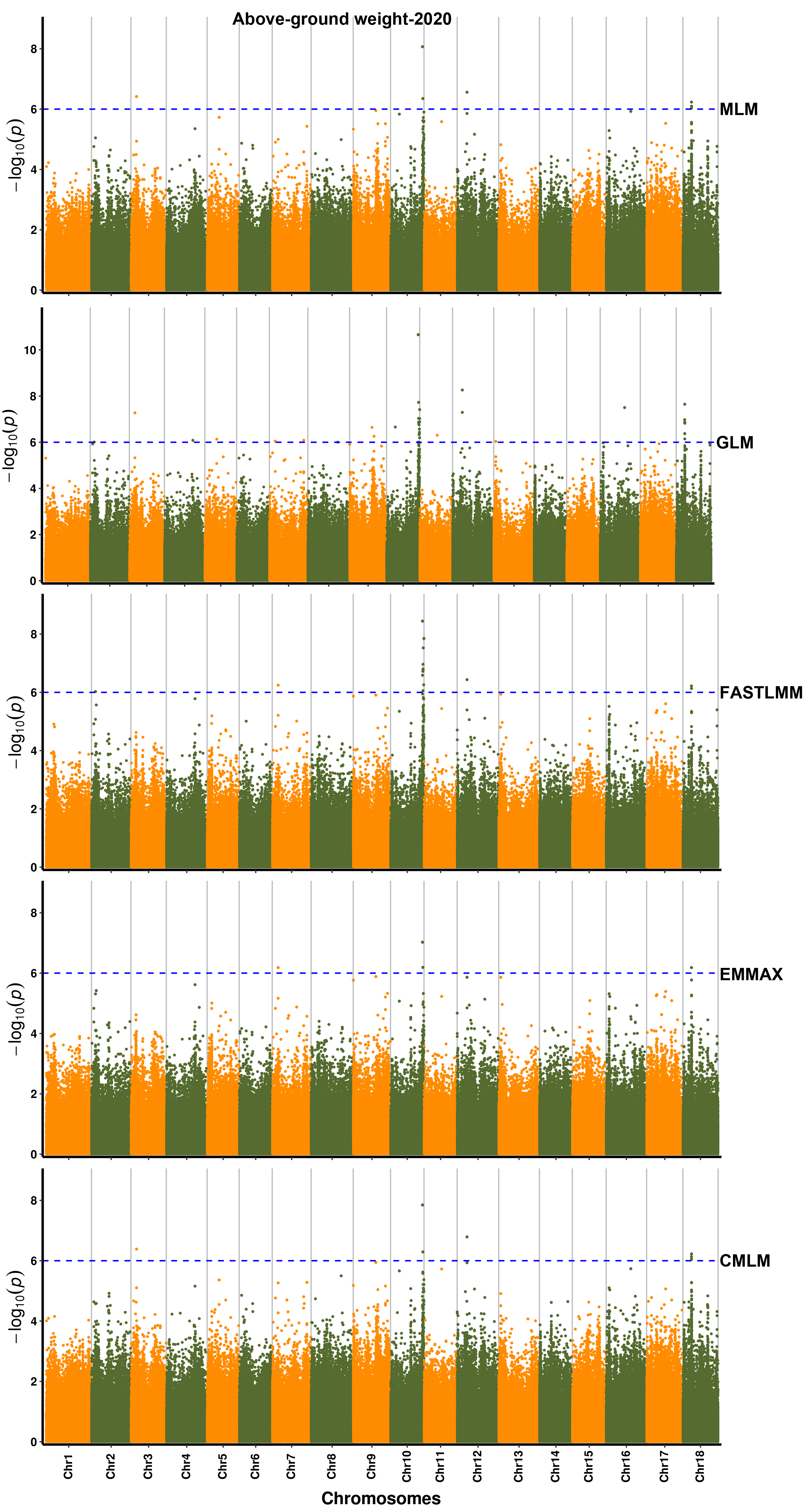


**
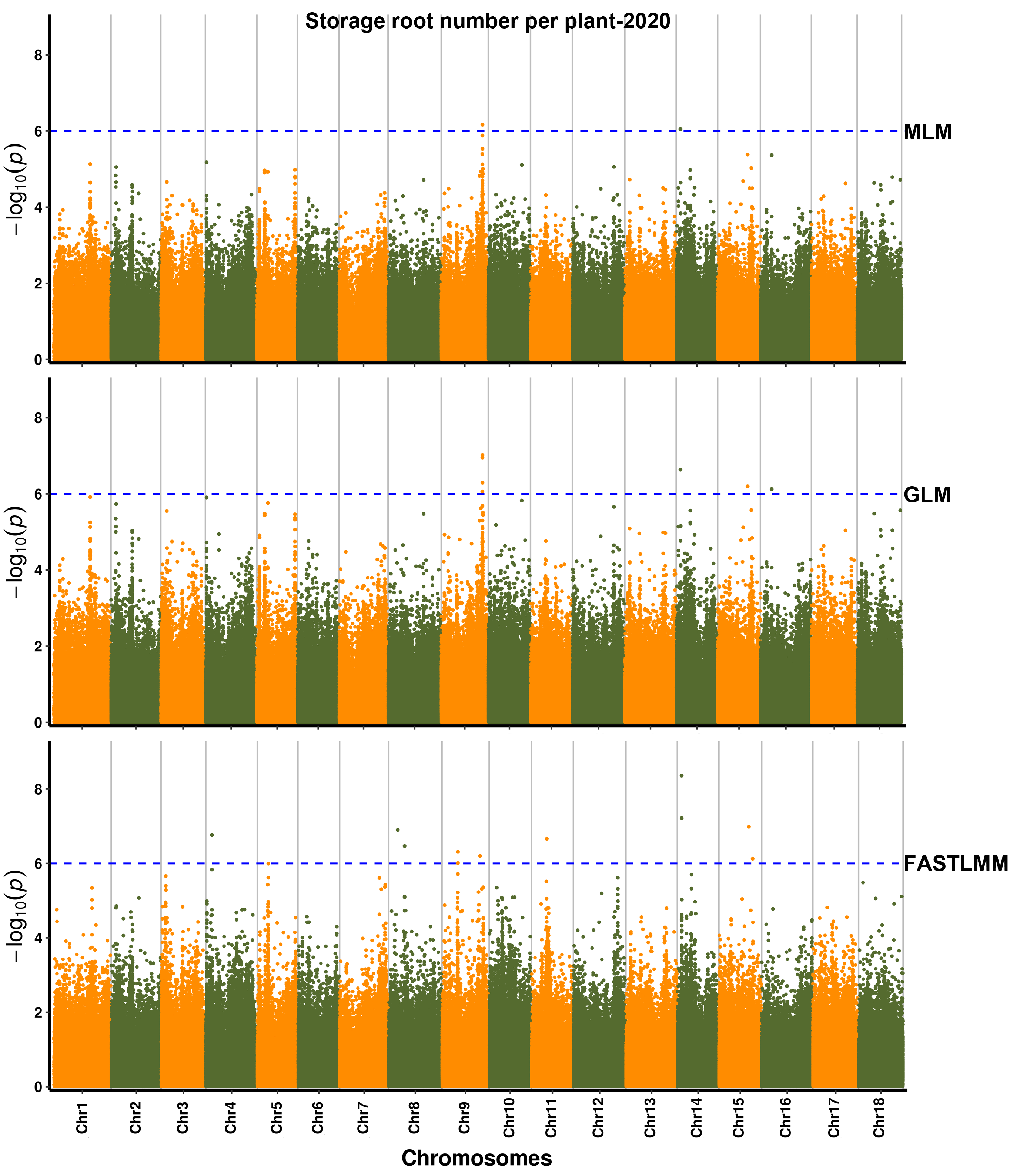
**

**
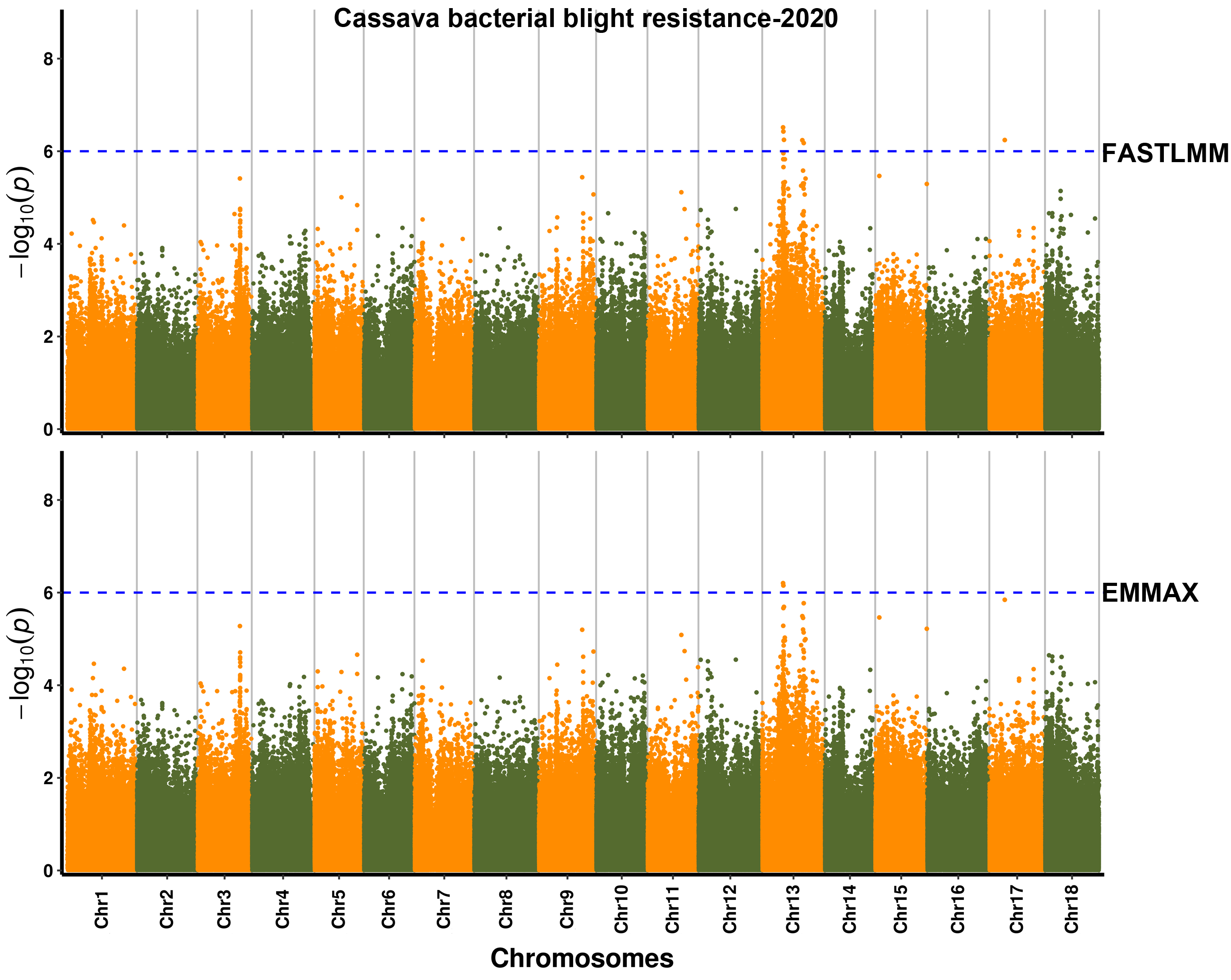
**

**
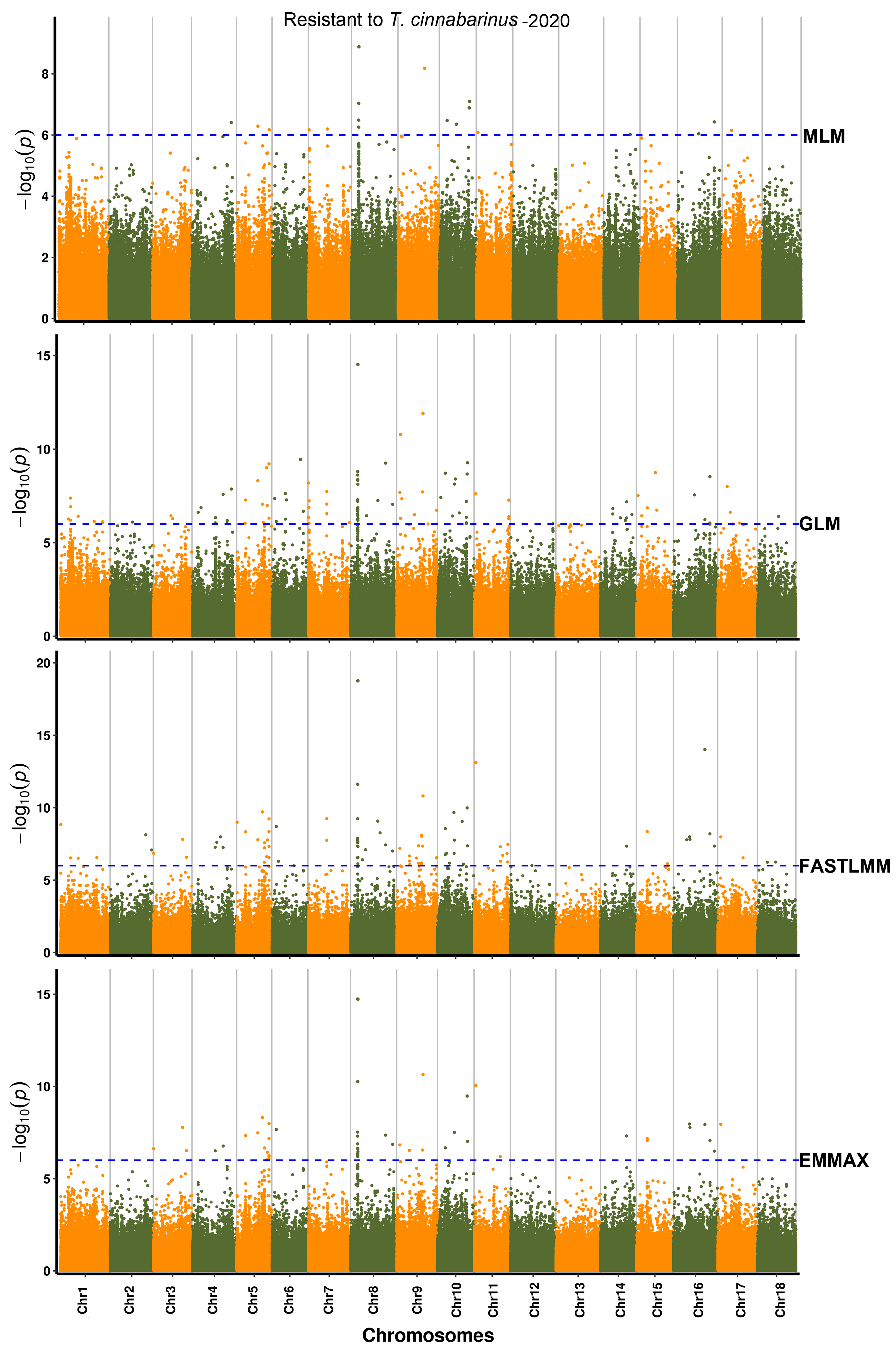
**

**Fig S3. Manhattan plots (–log_10_P > 6) for GWAS analysis of cassava agronomic traits.**

**
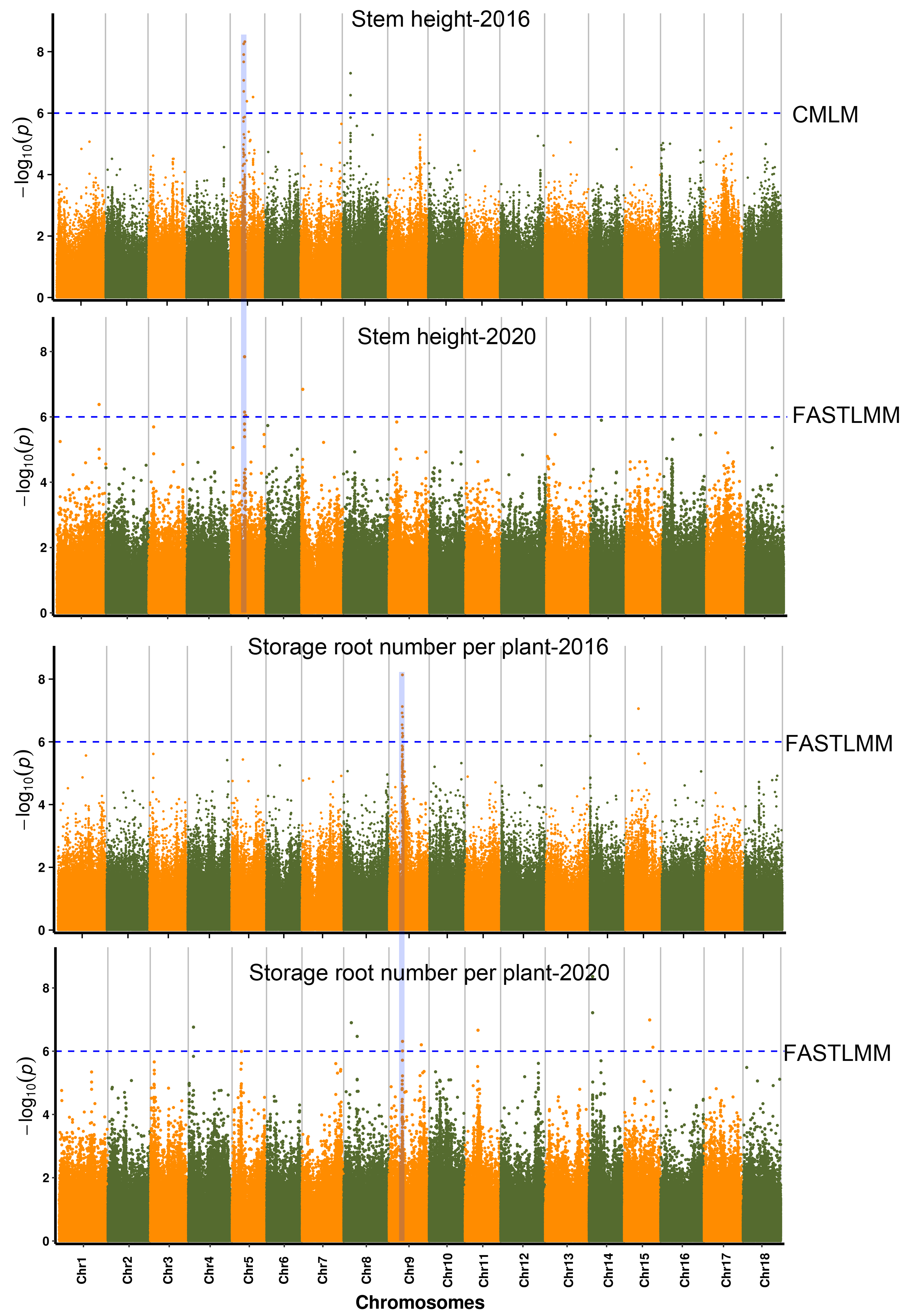
**

**Fig S4. Manhattan plots (–log_10_P > 6) of two repeatedly observed MTAs for stem height and storage root number per plant.** Purple lines indicate the position of the repeatedly observed MTAs.


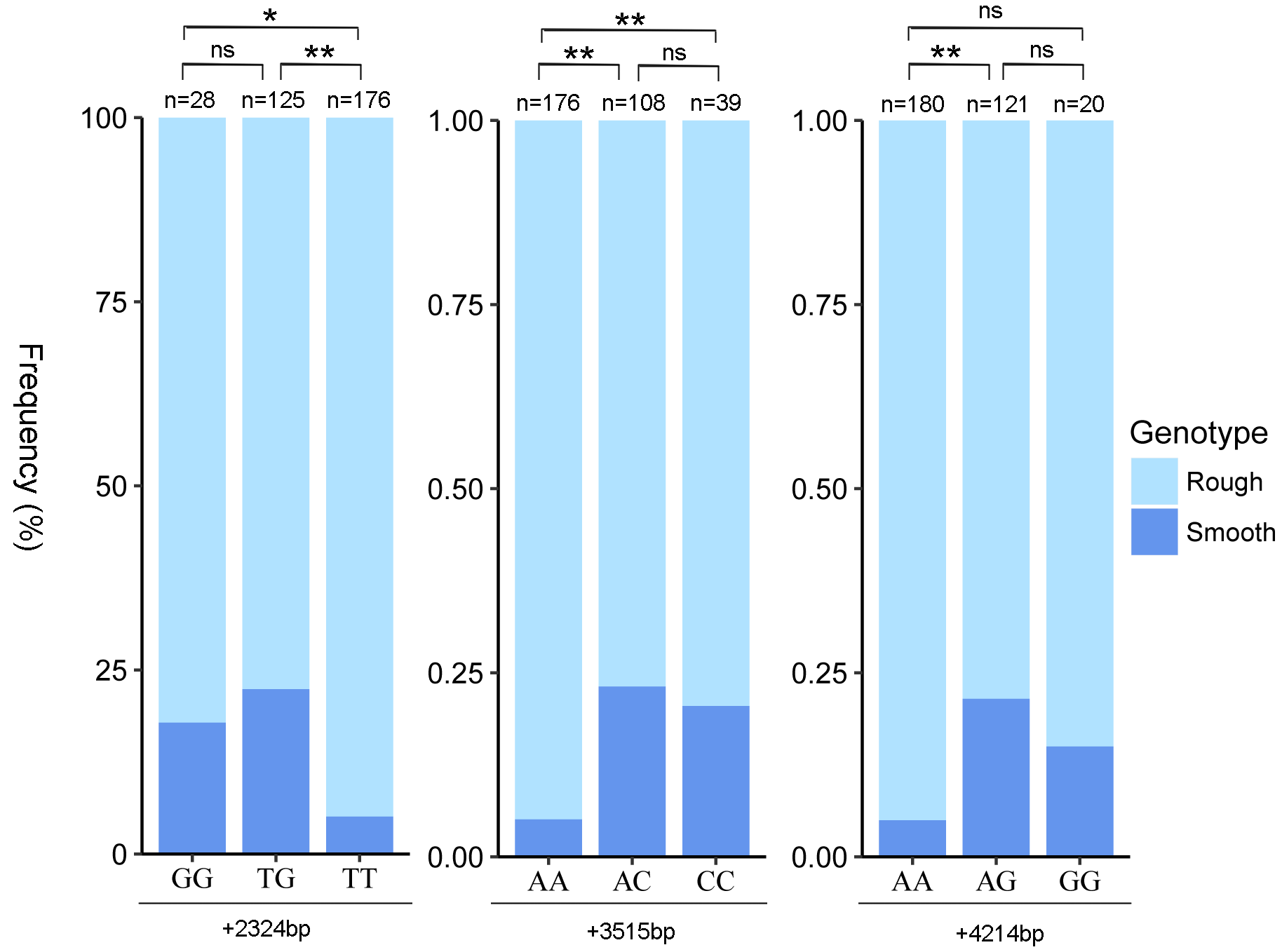


**Fig S5. Comparison of SR epidermal types based on the non-synonymous SNPs in *Sc10g012040*.** The significance of difference was derived with chi-square test (**P*< 0.05, ***P*< 0.01). The symbol *n* represents the number of accessions with the same genotype.


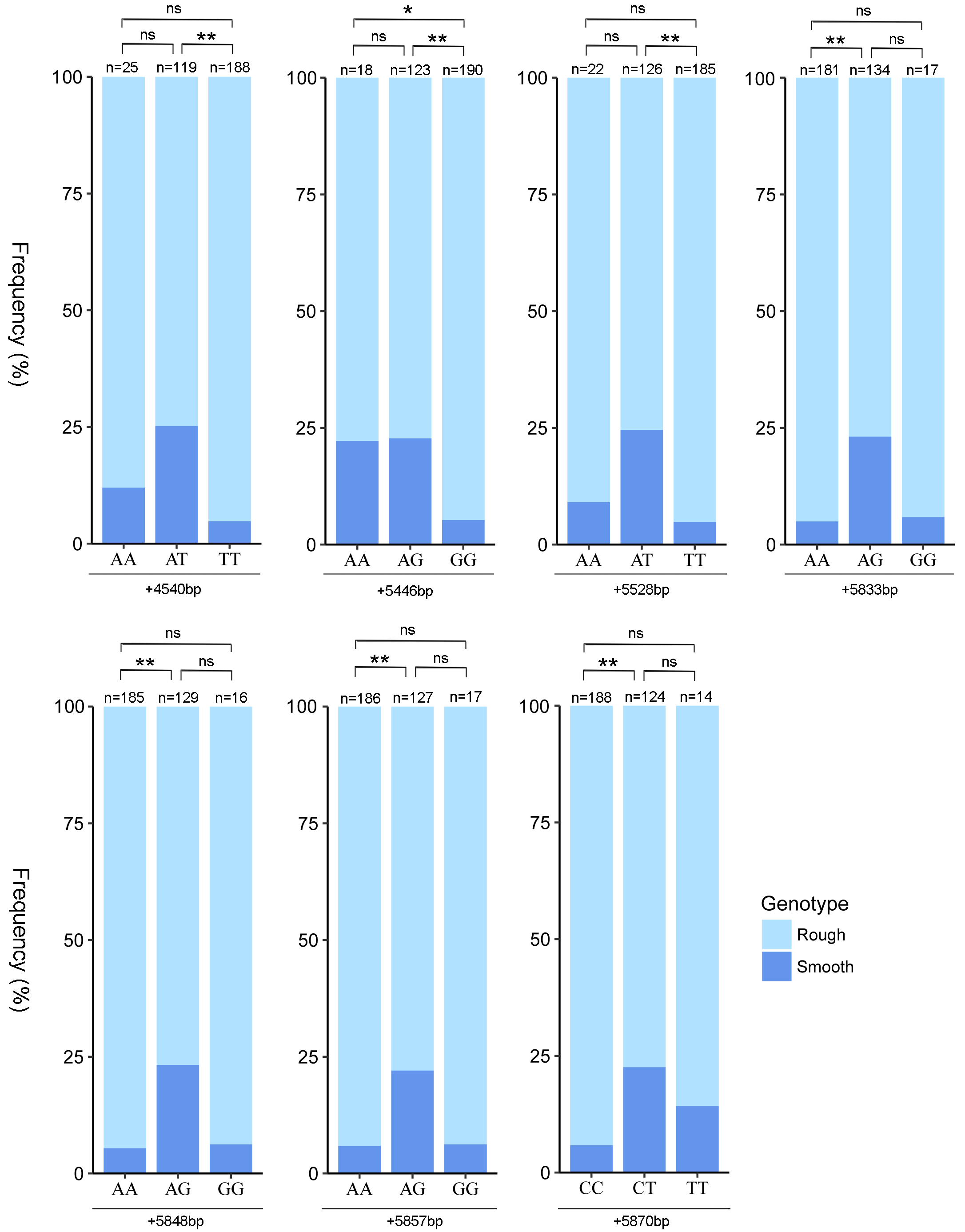


**Fig S6. Comparison of SR epidermal types based on the non-synonymous SNPs in *Sc10g012050*.** The significance of difference was derived with chi-square test (**P*< 0.05, ***P*< 0.01). The symbol *n* represents the number of accessions with the same genotype.


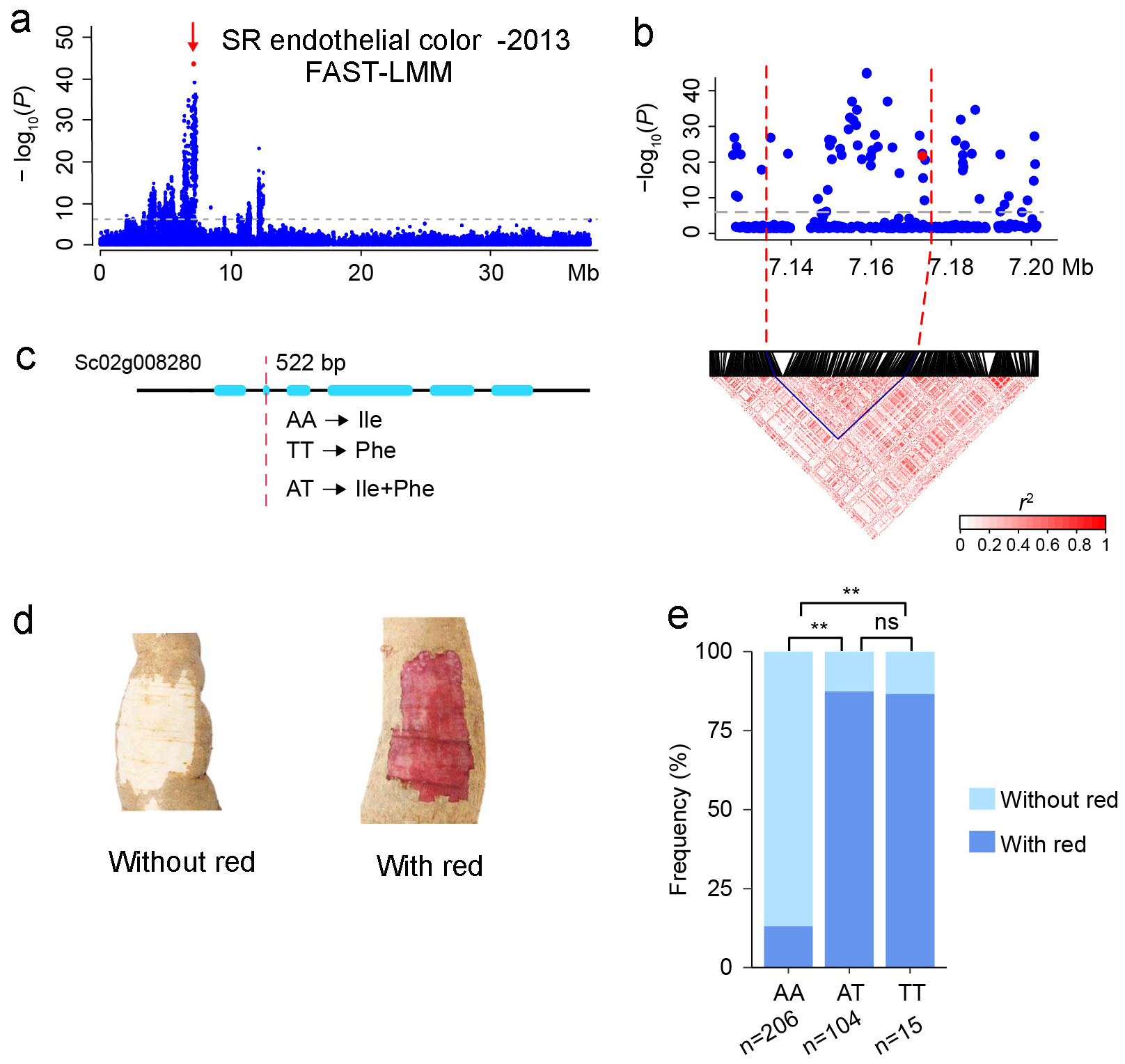


**Fig S7. GWAS identification of *Sc02g008280* as a candidate gene for SR endothelial color on chromosome 2.** (**a**) Manhattan plots for SR endothelial color on chromosome 2 using FAST-LMM. Red arrow indicates the significant GWAS peak. (**b**) Local manhattan plot (top) and linkage disequilibrium heat map (bottom). Red dashed lines indicate the candidate region. Red dot indicates the core SNP in the candidate gene. (**c**) *Sc02g008280* gene model. The dashed red lines represent the position of the non-synonymous SNP. (**d**) Images of SR endothelial color types. (**e**) Comparison of SR endothelial color types based on the core SNP. The significance of difference was derived with chi-square test (**P*< 0.05, ***P*< 0.01). The symbol *n* represents the number of accessions with the same genotype.

**
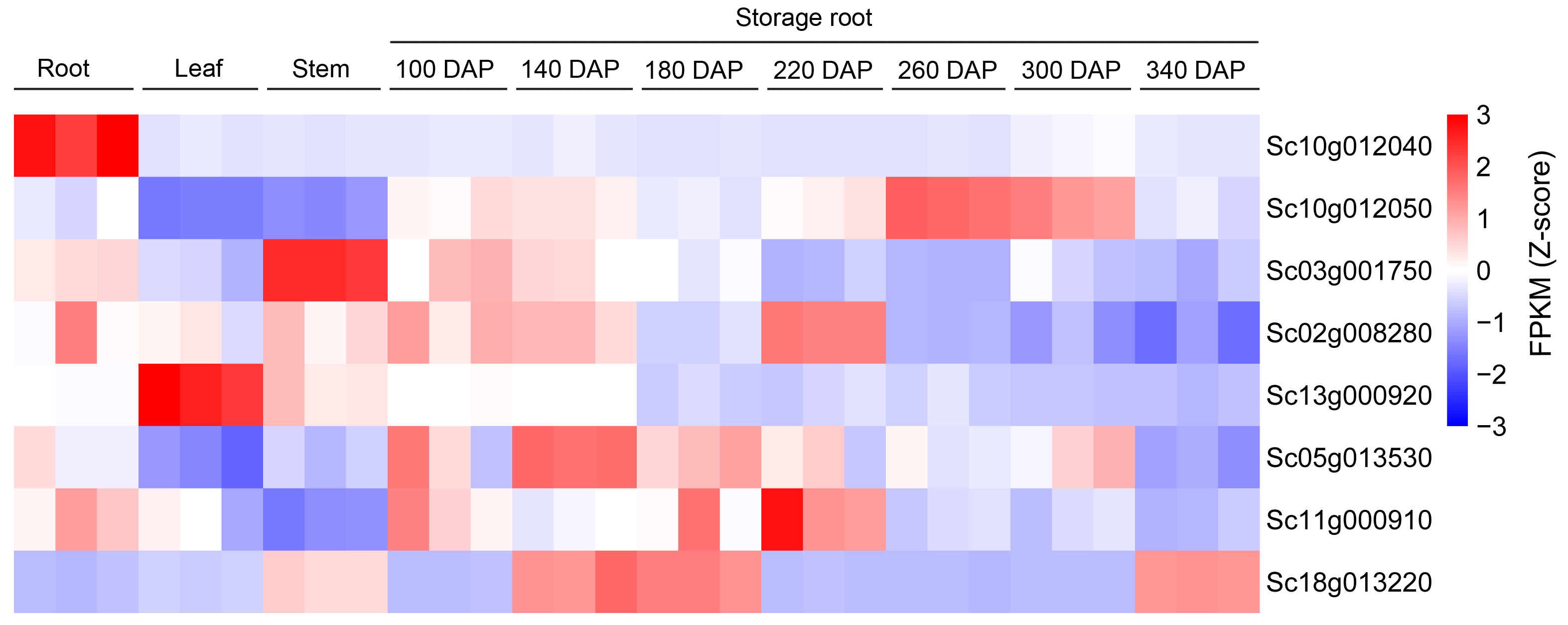
**

**Fig S8. Expression of candidate genes from GWAS analysis in different tissues and stages of storage root development.** The heatmap was constructed using Z-score value. Each sample contains three replicates. The root, leaf, and stem samples were collected from 100 days after planting (DAP). Storage roots samples include seven development stages collected from 100 to 340 days after planting (DAP) with intervals of 40 days.


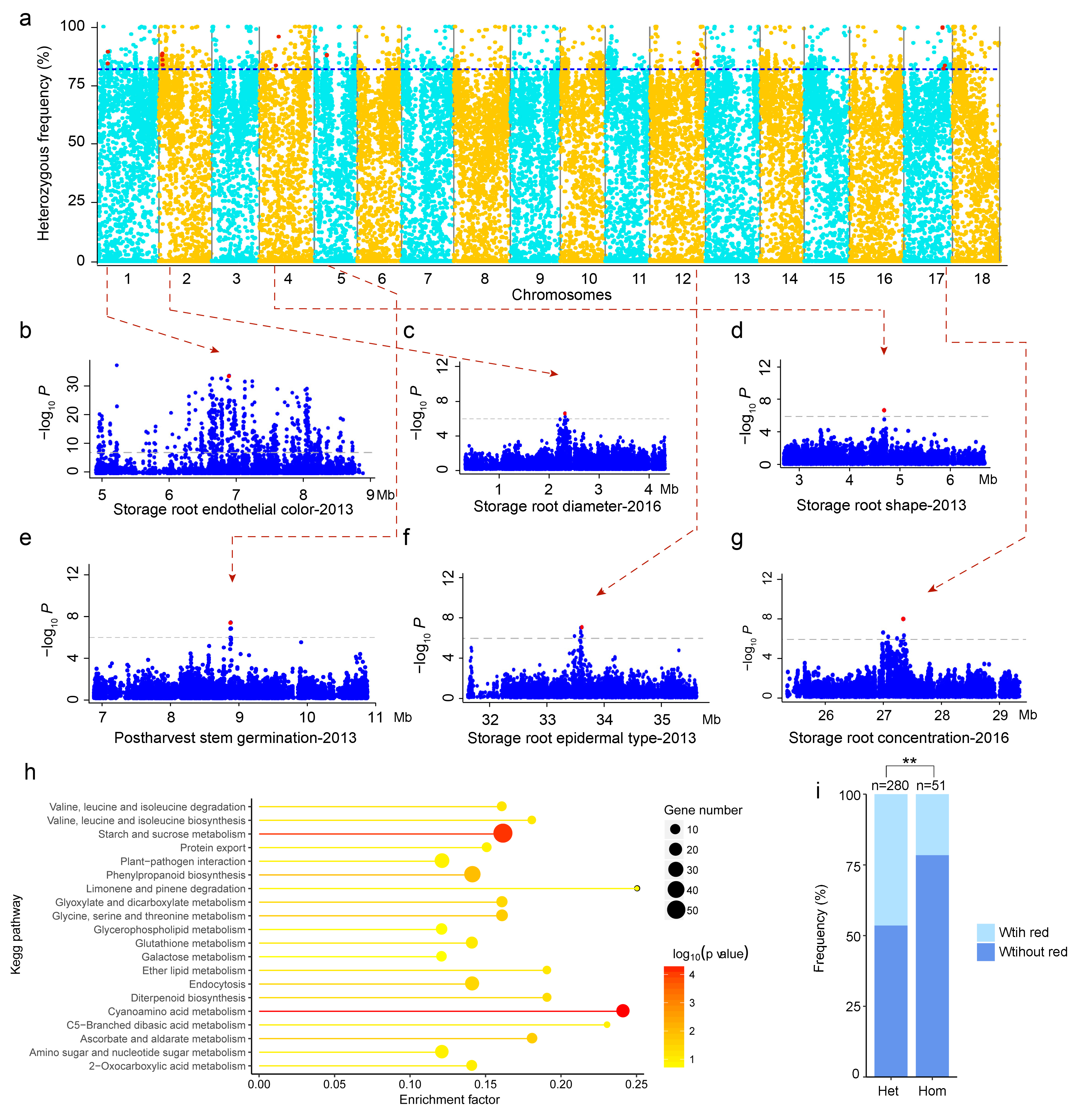


**Fig S9. Identification and screening of heterozygous blocks with high frequency in cultivars.** (**a**) Genome-wide distribution of heterozygous blocks from 374 cassava cultivars. The plot was drawn in 20-kb non-overlapping windows. The window that has more heterozygous sites than the average heterozygous SNPs (10.83 heterozygous SNPs per window) was considered as a heterozygous block in corresponding accession, while the contrary was regarded as a homozygous block. We calculated the population frequency of the heterozygous blocks in 374 cultivars to identify the highly heterozygous blocks. The sliding windows within the empirical top 5% of heterozygous frequency (blue dashed line) in the population were selected as highly heterozygous blocks. (**b-g**) Significant GWAS signals overlapped with highly heterozygous blocks. The red dots indicate the position of highly heterozygous blocks and GWAS signals. ±100 kb regions of GWAS signals were used to overlap with highly heterozygous blocks. (**h**) KEGG pathway enrichment of the genes within highly heterozygous blocks from panel (a). ‘Starch and sucrose metabolism’ is the most significantly enriched pathway among genes within heterozygous blocks with high frequency. (**i**) Comparison of SR endothelial color types based on the highly heterozygous block (Het) and homozygous block (Hom) at the block region of 6920000-6940000 on chromosome 1. There are 272 heterozygous haplotypes from 280 cassava accessions with the highly heterozygous block and trait values. The significance of difference was derived with chi-square test (**P*< 0.05, ***P*< 0.01). The symbol n represents the number of accessions with the highly heterozygous block or homozygous block.


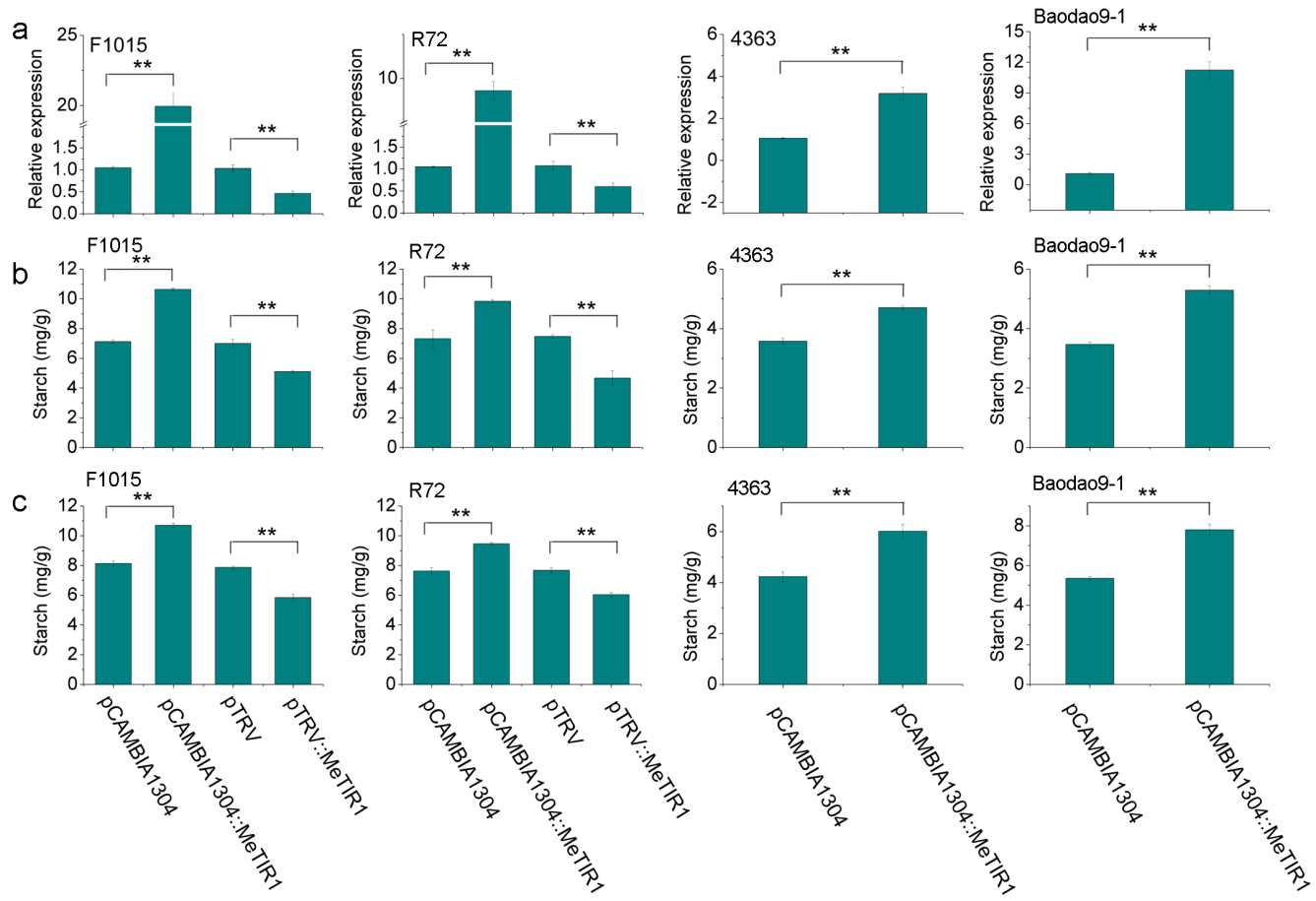


**Fig S10. Transient overexpression and silencing of *MeTIR1* affect starch content in leaves of four cassava cultivars (F1015, R72, 4363 and Baodao9-1).** (**a**) Expression of *MeTIR1* in leaves of transgenic cassava using qRT-PCR. *MeTIR1* showed higher expression in cassava leaves transformed with pCAMBIA1304::MeTIR1 than in cassava leaves transformed with pCAMBIA1304 (vector control) in four cultivars, suggesting effective overexpression of *MeTIR1* (***P*< 0.01, two-tailed *t*-test). *MeTIR1* showed lower expression in cassava leaves transformed with pTRV::MeTIR1 than in cassava leaves transformed with pTRV (vector control) in cultivars F1015 and R72, suggesting effective silencing of *MeTIR1* (***P*< 0.01, two-tailed *t*-test). Data are represented as mean ±s.d. (n = 3 biological replicates). (**b,c**) Quantification of starch content in leaves of transgenic cassava at 8:30 am (**b**) and at 8:30 pm (**c**), respectively. Cassava seedlings were cultured under a growth chamber with 14 h light (from 8:00 am to 10:00 pm)/10 h dark (from 10:00 pm to 8:00 am) cycle at 27 °C. Overexpression of *MeTIR1* in F1015 (GG allele), R72 (GG allele), 4363 (CG allele) and Baodao9-1 (CG allele) increased starch content, whereas silencing of *MeTIR1* in F1015 (GG allele) and R72 (GG allele) led to decreased starch content (***P*< 0.01, two-tailed *t*-test). Data are represented as mean ±s.d. (n = 4 biological replicates).


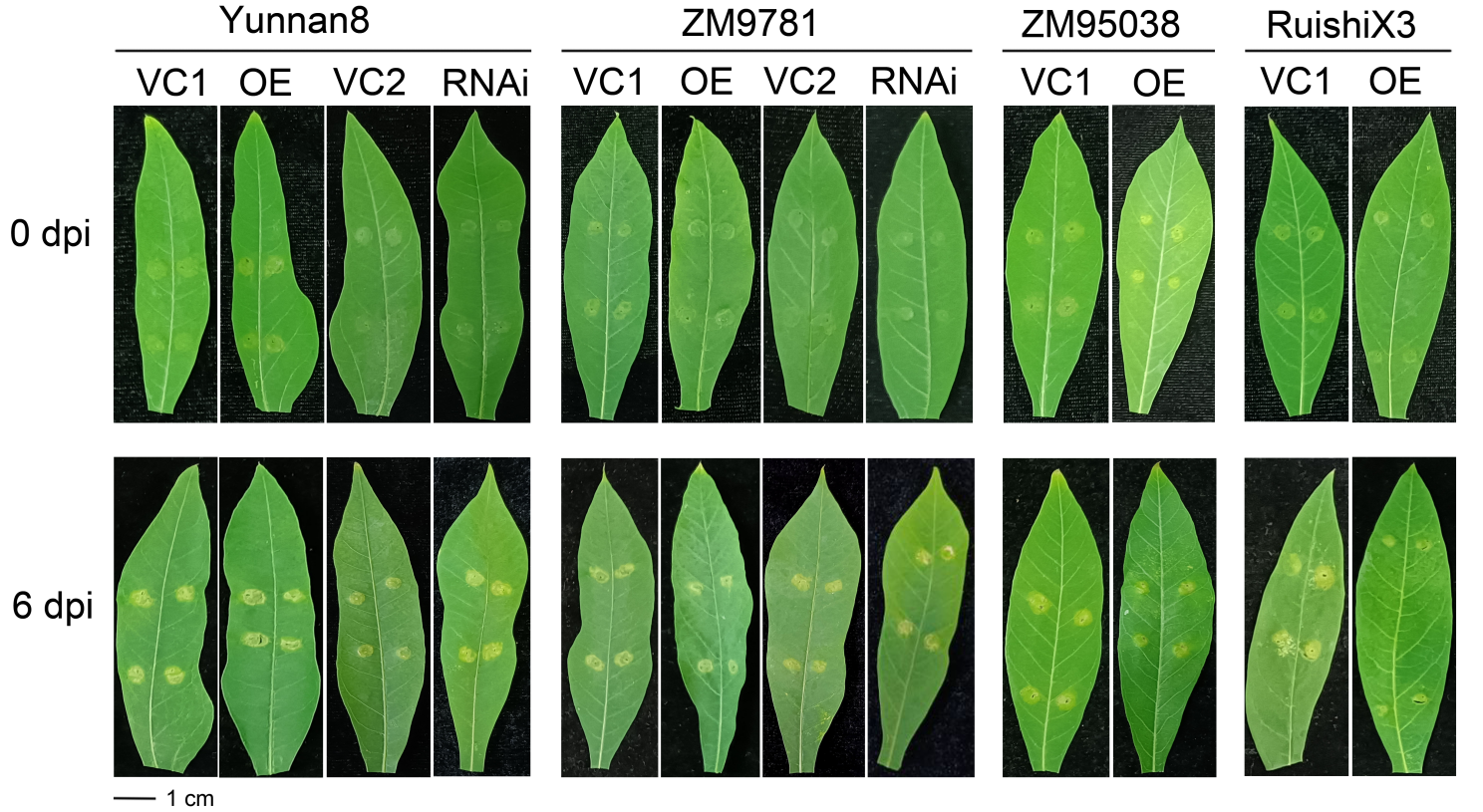


**Fig S11. Photos of cassava leaves transformed with pCAMBIA1304 (vector control, VC1), pCAMBIA1304::MeAHL17 (OE), pTRV (vector control, VC2) or pTRV::MeAHL17 (RNAi) in four cultivars at 0 and 6 days post inoculation.** The lesion areas of *MeAHL17*-overexpressed plants were smaller than those of VC1-transformed plants, whereas the lesion areas of *MeAHL17*-slienced plants were larger than those of VC2-transformed plants.
