# Supplementary material for: Resequencing of 388 cassava accessions identifies valuable loci and selection for variation in heterozygosity
Source: Genome Biol. 2021 Nov 16;22:316. doi: 10.1186/s13059-021-02524-7 (PMC8594203; doi:10.1186/s13059-021-02524-7)
Supplement: Supplementary file 23 — Additional file 23. Review history. [file 13059_2021_2524_MOESM23_ESM.docx]

Review History

**First round of review**

**Reviewer 1**

**Are you able to assess all statistics in the manuscript, including the appropriateness of statistical tests used?**

**Yes, and I have assessed the statistics in my report.**

**Comments to author:**

The manuscript of Hu et al. describes the analysis of "Resequencing of 388 cassava accessions identifies valuable loci and selection for variation in heterozygosity". The authors figure out the genomic variation map, agro trait loci by GWAS, heterozygous blocks during domestication and one case of allelic variation in MeTIR1 and MeAHL17. Some of the conclusions are lack of evidence to support. There are several issues with the manuscript that need to be addressed before the manuscript is published.

Major issues:

1, In the analyzed accessions, only 5 and 10 Wild Progenitor accessions from Africa and South America, respectively, how can you explain the domestication and dispersal route? In addition, the Breeding lines from Africa is also not enough for supporting the dispersal route that South America to Africa and finally to Asia. This is the serious problem for this analysis.

2, There is lack of evidence to support the dispersal route from Africa to Asia.

3, The authors should mark which 337 cassava accessions for GWAS, because 388 cassava accessions were used for the phylogenetic and principal component analyses.

4, It is very strange that why the authors could not divide few subgroups from the cultivars for analysis about divergence? Please explain it.

5, The GWAS results only based one location and only one year 2013, few phenotype traits measured in 2016. The phenotype data is lack of three replications, this is not acceptable for only published paper about GWAS, and it is the biggest problems for this article. It is also very strange that the authors used the 7 years ago's phenotype data for GWAS, why not use the year of 2017-2020?

6, There some very strong peak signaling, such as storage root endothelial color-2013 on Chr. 1 and Chr.2, storage root number per plant-2016 on Chr.9, etc. the authors should discuss these loci in the main text.

7, Fig 4d and 4j is completely same, please explain it.

8, Could you explain why the MeTIR1 gene is not in the Storage root starch content by GWAS?

9, In all the suppl tables, the format should be same, such as gene name should be italic.

**Reviewer 2**

**Are you able to assess all statistics in the manuscript, including the appropriateness of statistical tests used?**

Yes, and I have assessed the statistics in my report.

**Comments to author:**

The authors have re-sequenced 388 cassava accessions to identified 42 associated loci for 21 traits. The authors also connected the relationship between variation in heterozygosity and domesticated traits such as storage root and biotic stress. As results, that detected 81 selective sweeps with decreased heterozygosity and found some candidate genes for starch content like MeTIR1. The author have completed many significant efforts to compile and generate the presented work, however there are still some concerns, aspects of the writing for clarification and improved readability, as well as parts of the results that need to be validated to strengthen the results. Thus, the content of the current paper is not suitable for publication.

Major Concerns:

The broad scope and substantial discoveries are not reflected in the abstract, and it is not very clear that the variation in heterozygosity underlying associated 21 agronomic traits. How many variations in heterozygosity were significantly associated with key traits? For 548 genes in 81 selective sweeps, how about their function and related pathways? The process and result in base substitution is really necessary to confirm the hypothesis "allelic variation in heterozygosity is associated with key agronomic traits", thus, it is suggested to mention overexpressing and silenced cultivars. No description for the combinations of allelic variation in MeTIR1 and MeAHL17. The abstract needs re-written.

What is the value of high heterozygosity in cassava genome, especially for wild and cultivated accessions? More detail data should be provided to confirm the relationship between variation in heterozygosity and altered traits.

1. The author focused on variation in heterozygosity. Thus, they should detect the accuracy of heterozygous loci after SNP calling, excluding the interference of bioinformatics analysis. As it is well known, during analyzing the homozygous allelic variations in rice, maize and cotton, we try to eliminate the interference of heterozygous sites.

2. The identification of candidate genes in associate loci is too rough. There is no complete panel for screening and identification the key genes. According to experience, SNPs in adjacent genes also have significant variation. (1) The identification of LD block interval does not correspond to GWAS plot, especially for figure 2o, 2r and 2u. (2) In line 160-163, how many genes located in LD block on Chr.05 in signal for weight of cassava plants and the detail process for identifying the candidate gene Sc05g013530. Similar point were found in signal region on Chr.11 and Chr.12. Are there differences in gene expression level among different types of materials? (3) In figure 2, so many allelic variations were in Sc10g012040 and Sc03g001750, but only two haplotypes were compared without significance test, such as in 2f, 2i and 2m. What about another homozygous type.

3. The author identified the heterozygous loci in each accessions and detected trait segregation between heterozygous and homologous groups in highly heterozygous blocks using the Wilcoxon rank-sum test. Why use Wilcoxon ran-sum? Instead, the author could directly calculate the heterozygous frequency in the GWAS loci. Is there any support from other papers?

4. As to experiment for MeTIR1, the wild and cultivated species both contained GG, GT and TT at the same time. Compared to cultivar, the author only focused on decreased GT type. Actually, the decrease of TT type were is even more severe. How to exclude the influence of homozygous allelic variation on superior traits during domestication. For figure 4i, 4h and 4p, why not keep the same experiment group including GG, GT and TT. For figure 4e and 4k, why the pots in box-plot were more than N under the figure. From line 256 to line 261, it is very confusing which haplotype MeTIR1 in cassava of was used for transient overexpression and silencing. Weather the author overexpressed the corresponding gene type in F1015, R72, 4363 and baodao 9-1?

5. For MeAHL17, could the author clarify whether the AA in cultivars were derived from AG or GG in wild accessions, instead of just looking at AA and AG types. Actually, there is no experiment design for base substitution to identify the exact function characterization of "AG to AA", which was mentioned in abstract.

6. For MeAHL17 associated with cassava bacterial blight, in promoter region, what element? AG is more sensitive. Are there only AA and AG types? The expression level in Yunan8 and ZM9781 with treatment at 2 dpi should be compared to "Yunan8 and ZM9781 without treatment at dpi" in figure 5d. It is proved that both AA and AG can play a role. But there is no actual comparison between AA and AG. More data are needed to prove that AG is more sensitive than AA. And graphic data was necessary to make the function of MeAHL17 clearly.

Minor comments:

Line 119-120 Group II was constructed with several clades which should be more mentioned according to different origins, not a simple description.

Line 128-131 Although the FST values between cassava populations 129 from Brazil and Nigeria (0.120)

Line 133-134 As to figure 1d, line color is too close, red and green should be separated. How to define migration weight in 1e.

Line 142-143 "Based on 1,313,775 high-quality SNPs" should be revised to "Based on 1,313,775 high-quality SNPs from 337 cassava accessions".

Line 144-150 These descriptions should be removed to method section.

Line 396-397 As to "We found that balancing selection has had a role in maintaining genomic heterozygosity", it is a general knowledge not a result from these research.

For figure 2u	How to define the LD block? Obviously, the red line doesn't match.

For figure 6d Why there was no significance in SR weight between different types?

**Reviewer 3**

**Are you able to assess all statistics in the manuscript, including the appropriateness of statistical tests used?**

No, I do not feel adequately qualified to assess the statistics.

**Comments to author:**

**Major concerns**

**1. The authors mentioned that the whole panel contained 38 landraces, but in Fig. 1a-c, I cannot find the 'landraces' subgroup. Did the author combine them with 'cultivars'? The authors should explain these accessions.**

**2. In Fig. 2, 'epidermal type' seems like a qualitative trait (I am not sure); why authors found so many loci for this trait? Moreover, a total of 82 genes found in these LD blocks (Supp. Table 5), authors should explain the principle of candidate genes selection.**

**3. Line 191-203, I suggested authors creating a figure to show the overlapped genomic regions between the LD blocks identified by GWAS and highly heterozygous blocks, which could exhibit the significant effect of heterozygosity of trait-related loci better.**

**4. Line 206-209 and Fig. 3, authors separated the whole panel (n = 388) into wild progenitors (n = 14) and cultivars (n = 336 or 374) to compare the genetic diversity, the population size of two subgroups is not equal. I suggest the authors randomly selecting 14 accessions from the cultivar subgroup to compare genetic diversity.**

**5. Line 217-218, '…we scanned genomic regions that exhibit drastic decreases in heterozygosity above the 99%…' according to the authors' assumption, heterozygosity of most traits-related loci should be positive (Line 50-51, Line 188-189, Line 177-179). Hence, the heterozygosity of human-selected regions should increase during domestication; why authors defined the genomic regions with a decrease in heterozygosity as domestication signals?**

**6. I am a little confused about Fig. 4. Firstly, the selection signals (both for He and π) were not very high for SNP (11,848,921bp) within MeTIR1 (red dash line in Fig. 4d). I do not think this variation underwent strong selection. Perhaps some other variations (promoter or distant enhancer?) with greater differences of He and π signals between wilds and cultivars were the causal variations that impacted the expression level of MeTIR1, and further determined the SR starch content. Additionally, the genotypes of this SNP (GG, GT, and TT) are evenly distributed in wild accessions (Fig. 4g), but the SR starch content should be much lower in all wild accessions (Line 234-235), which indicated that this SNP obviously not associate with SR starch content. Why did the authors select this SNP? Also, The differences in SR starch content and SR weight were too small among genotypes. Although the authors performed further gene function experiments, it is still hard to judge that the two genes were genetically related to both traits. If authors could combine the GWAS results for SR starch content and SR weight with selection sweep results, I think the genotyping results could be better, and the conclusion could be more solid.**

**Minor concerns**

**1. In Fig.1a, there are gaps on Chr. 2 and Chr. 9 for 'Wild' and 'All' populations for the SNP density distribution. Please check. Also, I suggest the authors adding the titles of axis Y (SNP density).**

**2. How many SNPs were used for constructing phylogenetic tree and PCA analysis?**

**3. Line 491, why authors used 0.000001 as the P-value threshold for GWAS, is there any reference?**

**4. Because this manuscript used multi-models and phenotypes under multi-environments, for all the local Manhattan plots in the main-text figures, if authors directly indicate what model (MLM, GLM …) and environment (2013 or 2016) these plots belong on the figure (not only in the captions), it would be easier to for readers.**

**5. In Fig. 4, did the authors perform GWAS for 'storage root weight'? I did not find it in the Supplementary Figures.**

**6. Line 206-209, '…we split the cassava genome into heterozygous and homozygous regions according to heterozygous SNPs…', this sentence is too long and not very clear, did the authors mean to separate the population SNP-set into heterozygous and homozygous regions? Additionally, the FST value is the divergence level between two groups; I did not see which groups used to calculate the FST value here.**

**7. Line 486, does 'integrity > 0.8' mean 'missing < 20%'? If so, please use the same description as above (line 440).**

**Authors Response**

**Point-by-point responses to the reviewers’ comments:**

Reviewer #1:

1. The manuscript of Hu et al. describes the analysis of "Resequencing of 388 cassava accessions identifies valuable loci and selection for variation in heterozygosity". The authors figure out the genomic variation map, agro trait loci by GWAS, heterozygous blocks during domestication and one case of allelic variation in MeTIR1 and MeAHL17. Some of the conclusions are lack of evidence to support. There are several issues with the manuscript that need to be addressed before the manuscript is published.

*Reply: Thank you very much for your comments. Your insights and suggestions are extremely important and reasonable, which helped us to improve the quality of the manuscript and avoid errors in our interpretation. Based on your valuable suggestions, we have made careful revisions on our manuscript. We hope that these revisions are satisfactory.*

2. In the analyzed accessions, only 5 and 10 Wild Progenitor accessions from Africa and South America, respectively, how can you explain the domestication and dispersal route? In addition, the Breeding lines from Africa is also not enough for supporting the dispersal route that South America to Africa and finally to Asia. This is the serious problem for this analysis.

*Reply: Thank you for your criticism. The exact wild progenitors of cassava were identified until 2016 (Bredeson et al. 2016), so the collection of wild progenitors of cassava lagged behind. Currently, there are only 15 wild progenitors were released globally. Due to material limitations, current studies on domestication of cassava can only be based on these wild progenitors (Ramu et al. 2017). Therefore, we investigated the dispersal route of cassava using these wild progenitors and 34 landraces from different regions.*

*From your valuable comments, we realized that it is not appropriate to infer the dispersal route based on limited materials. In the revised manuscript, we re-analyzed population divergence and phylogenetic relationship of wild progenitors and landraces, including 18 accessions from South America, 16 accessions from Africa and 14 accessions from Asia. The sample information was supplemented in Table S4. On continent scale, the FST values between cassava populations from South America and Africa (0.071) and between populations from Africa and Asia (0.067) were lower than those between populations from South America and Asia (0.094). Cassava accessions from Africa clustered closely with the accessions from South America or Asia (Fig. 1d). On country scale, the FST values between cassava populations from Brazil and Nigeria (0.120) and between populations from Nigeria and China (0.088) were lower than those between populations from Brazil and China (0.149). Cassava accessions from Nigeria clustered closely with the accessions from Brazil or China (Fig. 1e). These results support a close phylogenetic relationship between cassava populations from South America and Africa as well as between populations from Africa and Asia (Lines 130-141).*

*References:*

*Ramu P, Esuma W, Kawuki R, Rabbi IY, Egesi C, Bredeson JV, Bart RS, Verma J, Buckler ES, Lu F. Cassava haplotype map highlights fixation of deleterious mutations during clonal propagation. Nat Genet. 2017, 49(6):959-63.*

*Bredeson JV, Lyons JB, Prochnik SE, Wu GA, Ha CM, Edsinger-Gonzales E, Grimwood J, Schmutz J, Rabbi IY, Egesi C, et al. Sequencing wild and cultivated cassava and related species reveals extensive interspecific hybridization and genetic diversity. Nat Biotechnol. 2016, 34(5):562-70.*

3. There is lack of evidence to support the dispersal route from Africa to Asia.

*Reply: We agree with your opinion that existing data are not enough to infer the dispersal route of cassava. In the revised manuscript, we revised the inappropriate description and demonstrated a close phylogenetic relationship between cassava populations from South America and Africa as well as between populations from Africa and Asia (Fig. 1d and 1e, Lines 130-141).*

4. The authors should mark which 337 cassava accessions for GWAS, because 388 cassava accessions were used for the phylogenetic and principal component analyses.

*Reply: We agree with your opinion. We performed a GWAS analysis for 33 morphological traits of 337 cassava accessions resequenced in the present study. The 337 accessions used for GWAS were marked in Table S1.*

5. It is very strange that why the authors could not divide few subgroups from the cultivars for analysis about divergence? Please explain it.

*Reply: Thank you for your suggestions. Although we tried to divide few subgroups based on phylogenetic and principal component analyses, there is no clear demarcation of geographic distribution patterns from cultivated cassava accessions. This is consistent with previous reports showing mixture of cultivars in different regions (Bredeson et al. 2016; Ramu et al. 2017). Because the extensive introduction and hybridization of cultivars may cause confusion in some cultivars, we used wild progenitors and landraces for population divergence and phylogenetic analyses in the revised manuscript.*

*References:*

*Bredeson JV, Lyons JB, Prochnik SE, Wu GA, Ha CM, Edsinger-Gonzales E, Grimwood J, Schmutz J, Rabbi IY, Egesi C, et al. Sequencing wild and cultivated cassava and related species reveals extensive interspecific hybridization and genetic diversity. Nat Biotechnol. 2016, 34(5):562-70.*

*Ramu P, Esuma W, Kawuki R, Rabbi IY, Egesi C, Bredeson JV, Bart RS, Verma J, Buckler ES, Lu F. Cassava haplotype map highlights fixation of deleterious mutations during clonal propagation. Nat Genet. 2017, 49(6):959-63.*

6. The GWAS results only based one location and only one year 2013, few phenotype traits measured in 2016. The phenotype data is lack of three replications, this is not acceptable for only published paper about GWAS, and it is the biggest problems for this article. It is also very strange that the authors used the 7 years ago's phenotype data for GWAS, why not use the year of 2017-2020?

*Reply: Thank you for your comments. Due to the difficulties in funding and personnel for investigation of many agronomic traits, the phenotype was measured for only two years. We used five statistical models to perform GWAS analysis (Fig. S2). The potential candidate genes that have annotation and transcripts were selected for further analyzing whether they are in the LD block region and have allelic variation associated with phenotypic differences (Lines 496-498). Our results were validated by multiple pieces of evidence. We found association between allelic variants of 9 genes and 7 key agronomic traits (Fig. 2, Fig. 5 and Fig. S3). The correlation between the function of the 9 genes identified from GWAS analysis and the phenotype is supported by relevant literatures (Lines 152-177). We also performed expression analysis for these candidate genes and functional verification of MeAHL17 (Fig. 5 and Fig. S4). These results support the potential roles of allelic variation in heterozygosity on key agronomic traits. These detailed analysis and supplementary data reduced false positives. Further work will be required to validate candidate genes underlying the key traits because of the limited phenotyping replicates for GWAS analysis (Lines 393-394). We hope that you find our results meaningful and can understand the difficulties.*

7. There some very strong peak signaling, such as storage root endothelial color-2013 on Chr. 1 and Chr.2, storage root number per plant-2016 on Chr.9, etc. the authors should discuss these loci in the main text.

*Reply: It is a good suggestion. We conducted a detailed analysis of the five strong peak signals, including storage root endothelial color-2013 on Chr. 1 and Chr. 2, storage root number per plant-2016 on Chr. 9, stem height-2013 on Chr. 6, and stem height-2016 on Chr. 5. A significant peak on chromosome 2 associated with SR endothelial color harbors Sc02g008280, which encodes a glycosyltransferase that is required for anthocyanin biosynthesis (Yonekura-Sakakibara et al. 2012). Cassava accessions carrying the AT or TT alleles within Sc02g008280 showed higher frequency of red endothelium of SR than those carrying the AA allele (Fig. S3). We have included these results in the revised manuscript (Lines 169-172).*

*In addition, we also identified allelic variations in candidate genes near other strong peak signals, but we did not find significant phenotypic differences between accessions carrying the heterozygous alleles and the homozygous alleles. For example, the candidate gene Sc09g008220 near a GWAS signal for storage root number per plant-2016 on Chr.9 encodes a SNARE protein involved in cell growth, division and communication (Karnahl et al. 2017). We just identified a homozygous allele (chr 9: 11,898,686) that leads to an Arg (TT) to Gly (CC) substitution in its coding sequence. The cassava accessions carrying the CC allele showed significantly more storage root number per plant than those carrying the TT allele. Because this study focused on the variation in heterozygosity, the association between homozygous allelic variation and phenotype was not included into the revised manuscript. In the discussion section, we added a statement: “In addition, it is possible to identify association between allelic variation in homozygous genotypes and agronomic traits from significant GWAS signals” (Lines 385-387).*

*References:*

*Karnahl M, Park M, Mayer U, Hiller U, Jürgens G. ER assembly of SNARE complexes mediating formation of partitioning membrane in Arabidopsis cytokinesis.*

*Elife. 2017, 6:e25327.*

*Yonekura-Sakakibara K, Fukushima A, Nakabayashi R, Hanada K, Matsuda F, Sugawara S, Inoue E, Kuromori T, Ito T, Shinozaki K, Wangwattana B, Yamazaki M, Saito K. Two glycosyltransferases involved in anthocyanin modification delineated by transcriptome independent component analysis in Arabidopsis thaliana. Plant J. 2012, 69:154-67.*

8. Fig 4d and 4j is completely same, please explain it.

*Reply: Thank you for your kind suggestions. Fig. 4d shows selection signal detected in MeTIR1 gene and Fig. 4j shows selection signal detected in MeAHL17 gene. Because MeAHL17 and MeTIR1 are located within the same selective sweep region on chromosome 2, the selection signal region as shown by the green rectangle is the same. The dashed red lines that represent the position of MeTIR1 and MeAHL17 are different.*

9. Could you explain why the MeTIR1 gene is not in the Storage root starch content by GWAS?

*Reply: Thank you for pointing this out. GWAS is used to detect the correlation strength between allelic variation and traits. Human selection not only resulted in the significant enrichment of the GG allele of MeTIR1, but also caused the very low frequency of the CC allele of MeTIR1 (Fig. 4g,4h). This may have an effect on decreasing the sensitivity of GWAS. Additionally, SR starch content is a quantitative trait controlled by multiple genes. Thus, the GWAS peaks near the potential candidate gene MeTIR1 associated with SR starch content are below the threshold.*

10. In all the suppl tables, the format should be same, such as gene name should be italic.

*Reply: Thank you for pointing this out. We formatted the supplementary tables and made gene name italic.*

Reviewer #2:

1. The authors have re-sequenced 388 cassava accessions to identified 42 associated loci for 21 traits. The authors also connected the relationship between variation in heterozygosity and domesticated traits such as storage root and biotic stress. As results, that detected 81 selective sweeps with decreased heterozygosity and found some candidate genes for starch content like MeTIR1. The author have completed many significant efforts to compile and generate the presented work, however there are still some concerns, aspects of the writing for clarification and improved readability, as well as parts of the results that need to be validated to strengthen the results. Thus, the content of the current paper is not suitable for publication.

*Reply: Thank you very much for your comments. Your insights and suggestions are extremely important and reasonable, which helped us to improve the quality of the manuscript and avoid errors in our interpretation. Based on your valuable suggestions, we have made careful revisions on our manuscript. We hope that these revisions are satisfactory.*

2. The broad scope and substantial discoveries are not reflected in the abstract, and it is not very clear that the variation in heterozygosity underlying associated 21 agronomic traits. How many variations in heterozygosity were significantly associated with key traits? For 548 genes in 81 selective sweeps, how about their function and related pathways? The process and result in base substitution is really necessary to confirm the hypothesis "allelic variation in heterozygosity is associated with key agronomic traits", thus, it is suggested to mention overexpressing and silenced cultivars. No description for the combinations of allelic variation in MeTIR1 and MeAHL17. The abstract needs re-written.

*Reply: We are grateful and fully agree. The abstract has been re-written as follows (Lines 42-65):*

*Background: Heterozygous genomes are widespread in outcrossing and clonally propagated crops, but the variation in heterozygosity underlying key agronomic traits and crop domestication remains largely unknown. Cassava is a staple crop in Africa and other tropical regions and has a highly heterozygous genome.*

*Results: We describe a genomic variation map from 388 re-sequenced genomes of cassava cultivars and wild accessions and identify 42 loci for 21 agronomic traits through a genome-wide association study. Eighteen allelic variations in heterozygosity for nine candidate genes are significantly associated with seven key agronomic traits. We detect 81 selective sweeps with decreasing heterozygosity and nucleotide diversity, harboring 548 genes, which are enriched in multiple biological processes including growth, development, hormone metabolisms and responses, and immune related processes. Artificial selection for decreased heterozygosity has contributed to the domestication of the large starchy storage root of cassava. Selection for homozygous GG allele in MeTIR1 during domestication contributes to increased starch content. Selection of homozygous AA allele in MeAHL17 is associated with increased both storage root weight and cassava bacterial blight (CBB) susceptibility. Transient overexpression and silencing verify the positive roles of MeTIR1 in increasing starch content and MeAHL17 in resistance to CBB. The allelic combinations in MeTIR1 and MeAHL17 may result in high starch content and resistance to CBB.*

*Conclusions: This study provides insights into allelic variation in heterozygosity associated with key agronomic traits and cassava domestication as well as offers valuable resources for the improvement of cassava and other highly heterozygous crops.*

3.What is the value of high heterozygosity in cassava genome, especially for wild and cultivated accessions? More detail data should be provided to confirm the relationship between variation in heterozygosity and altered traits.

*Reply: Thank you for your valuable suggestions. Previous studies reported that cassava genome has a high heterozygosity of 0.61% and 0.69-0.84% in wild progenitors and cultivars, respectively (Bredeson et al. 2016; Hu et al. 2021). This was included in the revised manuscript (Lines 101-102). Based on your valuable suggestions, more detailed data related to SNP validation (Table S3), GWAS (Fig. 2 and Fig. S3), selection of MeTIR1/MeAHL17 (Fig. 4 and Fig. 6), and functional analysis of MeAHL17 (Fig. S10) are provided to confirm the relationship between variation in heterozygosity and altered traits.*

*References:*

*Bredeson JV, Lyons JB, Prochnik SE, Wu GA, Ha CM, Edsinger-Gonzales E, Grimwood J, Schmutz J, Rabbi IY, Egesi C, et al. Sequencing wild and cultivated cassava and related species reveals extensive interspecific hybridization and genetic diversity. Nat Biotechnol. 2016, 34:562-70.*

*Hu W, Ji C, Shi H, Liang Z, Ding Z, Ye J, Ou W, Zhou G, Tie W, Yan Y, Yang J, Yang X, Wei Y, Jin Z, Xie J, Peng M, Wang W, Guo A, Xu B, Guo J, Chen S, Ma L, Wang M, Yang Zhou, Li X, Li R, Guo S, Xiao X, Wan Z, An F, Zhang J, Leng Q, Li Y, Ming R, Li K. Allele-defined genome reveals biallelic differentiation during cassava evolution. Mol Plant. 2021, 14: 851-854.*

4. The author focused on variation in heterozygosity. Thus, they should detect the accuracy of heterozygous loci after SNP calling, excluding the interference of bioinformatics analysis. As it is well known, during analyzing the homozygous allelic variations in rice, maize and cotton, we try to eliminate the interference of heterozygous sites.

*Reply: It is a reasonable concern. A total of 26 SNPs (including both heterozygous and homozygous SNPs among accessions) were randomly selected for PCR-based sequencing from 33 accessions. All PCR products were aligned against the target region of SC205 genome using Sequencher 5.4, and the genotypes were manually checked for each accession (Lines 437-440). We observed that the average accuracy of the SNP-calling is 94.2% by PCR-based sequencing (Table S3; Lines 119-120).*

5. The identification of candidate genes in associate loci is too rough. There is no complete panel for screening and identification the key genes. According to experience, SNPs in adjacent genes also have significant variation. (1) The identification of LD block interval does not correspond to GWAS plot, especially for figure 2o, 2r and 2u. (2) In line 160-163, how many genes located in LD block on Chr.05 in signal for weight of cassava plants and the detail process for identifying the candidate gene Sc05g013530. Similar point were found in signal region on Chr.11 and Chr.13. Are there differences in gene expression level among different types of materials? (3) In figure 2, so many allelic variations were in Sc10g012040 and Sc03g001750, but only two haplotypes were compared without significance test, such as in 2f, 2i and 2m. What about another homozygous type.

*Reply: We are sorry for the unclear description. The screening and identification of candidate genes from GWAS analysis are demonstrated as follows. First, the GWAS analyses were performed using different statistical models of GLM, MLM, CMLM, EMMAX and FAST-LMM. MTAs supported at least by two statistical models are defined as valid MTAs. Second, the genes located in 100kb windows which centered on the MTAs were considered as potential candidate genes for further analysis. Third, the potential candidate genes that have annotation and transcripts were selected for further analyzing whether they are in the LD block region and have allelic variation associated with phenotypic differences. We have included the principle of candidate gene selection in the methods section. (Lines 485-498). (1) In the original manuscript, red dashed lines indicated the region between GWAS SNP and candidate gene within LD block. We are sorry that this inappropriate displaying may have caused a misunderstanding of the accuracy of GWAS analysis. We agree that red dashed lines should indicate the whole region of the LD block. We have corrected this inappropriate displaying for all the identified candidate genes from GWAS analysis in Fig. 2. (2) The process for identifying the candidate genes Sc05g013530 in chr. 5, Sc11g000910 in chr. 11 and Sc18g013220 in chr. 13 were performed as above described. We examined the expression levels of Sc05g013530, Sc11g000910 and Sc18g013220 among different types of materials and did not find significant difference. This implied that the effect of non-synonymous mutations identified in these candidate genes on phenotypic variations may not be achieved through affecting expression changes. (3) From original Fig. 2f, 2i, 2m, we thought that there was no obvious difference in frequency of phenotypes between heterozygous genotype and another homozygous genotype, and thus the phenotype of another homozygous genotype was not exhibited. Based on your valuable suggestions, we realized that the lack of displaying of another homozygous haplotype without statistical analysis is not rigorous. In the revised manuscript, we have included another genotype for each candidate gene (except for Sc05g013530 containing only two genotypes at the core position) and performed statistical analysis. The results support the potential roles of allelic variation in heterozygosity on key agronomic traits (Fig. 2).*

6.The author identified the heterozygous loci in each accessions and detected trait segregation between heterozygous and homologous groups in highly heterozygous blocks using the Wilcoxon rank-sum test. Why use Wilcoxon ran-sum? Instead, the author could directly calculate the heterozygous frequency in the GWAS loci. Is there any support from other papers?

*Reply: Thank you for your suggestions. Both wilcoxon rank-sum test and two tailed t-test were used to detect the significance of trait segregation between heterozygous and homologous groups in the revised manuscript, and finally identified 79 highly heterozygous blocks that are associated with starch accumulation in the storage root (Table S10). We further observed 20 highly heterozygous blocks that overlapped with 6 GWAS signals (Table S11, Fig. S8). Moreover, cassava accessions carrying the highly heterozygous block on chromosome 1 showed higher frequency of red endothelium of SR than those carrying the corresponding homozygous block (Fig. S8i). These results further support the hypothesis that variation in heterozygosity is associated with key agronomic traits (Lines 203-214).*

*At present, there is no report demonstrating the association between highly heterozygous blocks and agronomic traits. Artificial selection has shaped crop genomes to meet human demands and has also resulted in the near fixation of a portion of the genome (Lin et al. 2014), whereas heterozygosity is associated with retaining genetic diversity (Fu et al. 2015). There is a need to utilize genetic diversity in germplasm resources for crop improvement (Varshney et al. 2017). We thus try to identify and screen highly heterozygous blocks associated with agronomic traits. We also directly calculated the heterozygous frequency of the 42 GWAS loci and found that the highest frequency of heterozygous loci only reaches 39.6% among these GWAS loci.*

*References:*

*Lin T, Zhu G, Zhang J, Xu X, Yu Q, Zheng Z, Zhang Z, Lun Y, Li S, Wang X, et al. Genomic analyses provide insights into the history of tomato breeding. Nat Genet. 2014, 46(11):1220-6.*

*Fu YB. Understanding crop genetic diversity under modern plant breeding. Theor Appl Genet. 2015, 128(11):2131-42.*

*Varshney RK, Saxena RK, Upadhyaya HD, Khan AW, Yu Y, Kim C, Rathore A, Kim D, Kim J, An S, et al. Whole-genome resequencing of 292 pigeonpea accessions identifies genomic regions associated with domestication and agronomic traits. Nat Genet. 2017, 49(7):1082-8.*

7. As to experiment for MeTIR1, the wild and cultivated species both contained GG, GT and TT at the same time. Compared to cultivar, the author only focused on decreased GT type. Actually, the decrease of TT type were is even more severe. How to exclude the influence of homozygous allelic variation on superior traits during domestication. For figure 4i, 4h and 4p, why not keep the same experiment group including GG, GT and TT. For figure 4e and 4k, why the pots in box-plot were more than N under the figure. From line 256 to line 261, it is very confusing which haplotype MeTIR1 in cassava of was used for transient overexpression and silencing. Weather the author overexpressed the corresponding gene type in F1015, R72, 4363 and baodao 9-1?

*Reply: We are sorry for the inappropriate analysis and description. Based on the Reviewer3’s concerns, the genotypes (GG, GT, and TT) of the SNP (chr 2: 11,848,921) in MeTIR1 is not a significant variation associated with SR starch content because of its evenly distribution in wild accessions. Thus, we re-analyzed variations of MeTIR1 in wild and cultivated accessions and its association with SR starch content. We found that a SNP in the upstream region (-5,067bp) of MeTIR1 might be associated with MeTIR1 expression and domestication (Fig. 4f, 4g). Then, we investigated the effect of three types of allelic variation (CC, CG, and GG) on expression of MeTIR1 and SR starch content according to your valuable suggestions (Fig. 4h, 4i). Both CG and CC alleles contributed to the increase of GG frequency during cassava domestication (Fig. 4g). SR starch content is significantly higher in accessions carrying the GG allele than in accessions carrying the CG allele, but there is no significance for SR starch content between accessions carrying GG allele and accessions carrying CC allele (Fig. 4h). This clues that selection of homozygous GG allele from heterozygous CG allele in MeTIR1 may be associated with increased SR starch content. Because the low frequency of CC allele has a potential influence on statistical analysis of phenotype (Fig. 4h), we revised the description as “Selection for homozygous GG allele in MeTIR1 during domestication contributes to increased starch content” in the revised manuscript (Lines 259-273).*

*We have added relevant expression data in Fig. 4i and 4p to keep the same experiment group. For Figure 4e and 4k, the symbol n represents the number of accessions. Each accession contains three biological replicates. When drawing the box plots, the replicates for each sample were indicated as points. To avoid misunderstandings, we have removed the points in the box plots and made a sample description in the figure legend (Lines 1023-1025).*

*In the original manuscript, because the core SNP is located in the coding sequence of MeTIR1, the coding sequence of MeTIR1 that carries the monoallele G were amplified from SC205 accession to overexpress in four cultivars F1015 (GG allele), R72 (GG allele), 4363 (GT allele) and Baodao9-1 (GT allele). MeTIR1-specific region was amplified and used to silence MeTIR1 in F1015 (GG allele) and R72 (GG allele). In the revised manuscript, the core SNP variation located in the upstream of MeTIR1 may affect phenotype through regulating expression of MeTIR1. From Figure S9, we observed that overexpression and silence of MeTIR1 in multiple materials significantly affect expression levels of MeTIR1 and starch content. These results support the positive function of MeTIR1 on increasing starch content (Lines 266-273).*

8. For MeAHL17, could the author clarify whether the AA in cultivars were derived from AG or GG in wild accessions, instead of just looking at AA and AG types. Actually, there is no experiment design for base substitution to identify the exact function characterization of "AG to AA", which was mentioned in abstract.

*Reply: We are sorry for the inappropriate analysis and description. Based on your valuable suggestions, we compared the frequency, phenotype and expression of accessions carrying AA allele with accessions carrying AG or GG alleles. Both AG and GG alleles contributed to the increase of AA frequency during cassava domestication (Fig. 4n). Storage root weight is significantly higher in accessions carrying the AA allele than in accessions carrying the AG allele, but there is no significance for storage root weight between accessions carrying AA allele and accessions carrying GG allele due to the very low frequency of GG allele (Fig. 4o). The expression of MeAHL17 is significantly different among the accessions carrying the three alleles (Fig. 4p). Because the low frequency of GG allele has a potential influence on statistical analysis of phenotype and inappropriate description of base substitution, we revised the description as “selection for homozygous AA allele in MeAHL17 may be associated with the increased SR weight during cassava domestication” throughout the revised manuscript (Lines 279-290).*

9. For MeAHL17 associated with cassava bacterial blight, in promoter region, what element? AG is more sensitive. Are there only AA and AG types? The expression level in Yunan8 and ZM9781 with treatment at 2 dpi should be compared to "Yunan8 and ZM9781 without treatment at dpi" in figure 5d. It is proved that both AA and AG can play a role. But there is no actual comparison between AA and AG. More data are needed to prove that AG is more sensitive than AA. And graphic data was necessary to make the function of MeAHL17 clearly.

*Reply: Thank you for pointing this out. After careful checking, we did not find the variation of MeAHL17 in core element. The effect of allelic variation in promoter region on transcription may involve a series of complex regulation, including transcription factor, epigenetic modification, and miRNA. Due to the very low frequency of GG allele (5/322) (Fig. 4o), the accessions carrying GG allele was not covered in phenotyping of the cassava bacterial blight resistance.*

*We are sorry for the wrong description of the expression of MeAHL17 in different cultivars in the original manuscript. Compared with 0 dpi, expression of MeAHL17 was induced 8.7-fold at 2 dpi in Yunnan8 and ZM9781 (with AG allele) and 8.2-fold at 2 dpi in ZM95308 and RuishiX3 (with AA allele). This indicates the degree of induction for MeAHL17 after Xam infection is not significantly differed between accessions carrying AG allele and those carrying AA allele. From Figure 5d, we found that the transcripts of MeAHL17 in bacterial blight-susceptible accessions (ZM95308 and RuishiX3 carrying the AA allele) were significantly lower than those in bacterial blight-resistant accessions (Yunnan8 and ZM9781 carrying the AG allele) at both 0 dpi and 2 dpi. These results implies that this allelic variation does not affect the degree of induction for MeAHL17 after Xam infection, but has an effect on MeAHL17 transcripts. This is also supported by dual luciferase assay showing that the promoter regions of MeAHL17 carrying G had higher activity than those carrying A (Fig. 4m). The original description was revised as “Although the transcripts of MeAHL17 in all the four accessions were largely induced after Xam infection at 2 days post inoculation (dpi), the transcripts of MeAHL17 in bacterial blight-susceptible accessions (ZM95308 and RuishiX3 carrying the AA allele) were significantly lower than those in bacterial blight-resistant accessions (Yunnan8 and ZM9781 carrying the AG allele) at both 0 dpi and 2 dpi (Fig. 5d).” (Lines 304-309)*

*Graphic data was included into the revised manuscript to verify the function of MeAHL17 on conferring CBB resistance (Fig. S10). Consistent with the bacterial count (Fig. 5f), the lesion areas of MeAHL17-overexpressed plants were smaller than those of VC1-transformed plants, whereas the lesion areas of MeAHL17-slienced plants were larger than those of VC2-transformed plants (Fig. S10).*

10. Minor comments:

[1] Line 119-120 Group II was constructed with several clades which should be more mentioned according to different origins, not a simple description.

*Reply: Thank you for your suggestions. Although we tried to divide few subgroups based on phylogenetic and principal component analyses, there is no clear demarcation of geographic distribution patterns from cultivated cassava accessions. This is consistent with previous reports showing mixture of cultivars from different regions (Bredeson et al. 2016; Ramu et al. 2017). Because the extensive introduction and hybridization of cultivars may cause confusion in some cultivars, we used wild species and landraces for population divergence and phylogenetic analyses (Fig. 1d, 1e). The results indicate a close phylogenetic relationship between cassava populations from South America and Africa as well as between populations from Africa and Asia (Lines 130-141).*

*References:*

*Bredeson JV, Lyons JB, Prochnik SE, Wu GA, Ha CM, Edsinger-Gonzales E, Grimwood J, Schmutz J, Rabbi IY, Egesi C, et al. Sequencing wild and cultivated cassava and related species reveals extensive interspecific hybridization and genetic diversity. Nat Biotechnol. 2016, 34(5):562-70.*

*Ramu P, Esuma W, Kawuki R, Rabbi IY, Egesi C, Bredeson JV, Bart RS, Verma J, Buckler ES, Lu F. Cassava haplotype map highlights fixation of deleterious mutations during clonal propagation. Nat Genet. 2017, 49(6):959-63.*

[2] Line 128-131 Although the FST values between cassava populations 129 from Brazil and Nigeria (0.120)

*Reply: We agree with your opinion. It is inappropriate to infer the dispersal route based on FST values of limited materials. In the revised manuscript, we re-analyzed the populations divergence and phylogenetic relationship of wild progenitors and landraces, including 18 accessions from South America, 16 accessions from Africa and 14 accessions from Asia. The sample information was supplemented in Table S4. The results demonstrate a close phylogenetic relationship between cassava populations from South America and Africa as well as between populations from Africa and Asia (Lines 130-141).*

[3] Line 133-134 As to figure 1d, line color is too close, red and green should be separated. How to define migration weight in 1e.

*Reply: Thank you for pointing this out. We redrew the line color to clearly distinguish different groups (Fig. 1). The migration weight was defined using TReeMixv1.12 (Pickrell et al. 2012). The calculation method is briefly described as follows. First, the maximum likelihood graph is searched based on the composite likelihood. Second, the maximum likelihood branch lengths and migration weights are identified. Third, the optimizing of the branch lengths and weights are iterated. Four, the optimal weight (between zero and one) on each migration edge is identified using a a golden section search. Finally, the weight of each migration edge is optimized in turn. Because the gene flow analysis did not fully support the dispersal route of cassava, we have removed this result from the revised manuscript according to Reviewer #1’s comments.*

*Reference:*

*Pickrell JK, Pritchard JK. Inference of population splits and mixtures from genome-wide allele frequency data. PLoS Genet. 2012;8(11):e1002967.*

[4] Line 142-143 "Based on 1,313,775 high-quality SNPs" should be revised to "Based on 1,313,775 high-quality SNPs from 337 cassava accessions".

*Reply: Thank you for your revision. "Based on 1,313,775 high-quality SNPs" has been revised to "Based on 1,313,775 high-quality SNPs from 337 cassava accessions" (Lines 147-148).*

[5] Line 144-150 These descriptions should be removed to method section.

*Reply: We agree with your opinion. These descriptions have been moved to method section (Lines 486-490).*

[6] Line 396-397 As to "We found that balancing selection has had a role in maintaining genomic heterozygosity", it is a general knowledge not a result from these research.

*Reply: We agree with your opinion. This description has been removed from the revised manuscript.*

[7] For figure 2u How to define the LD block? Obviously, the red line doesn't match.

*Reply: Thank you for pointing this out. In the original manuscript, red dashed lines indicate the region between GWAS SNP and candidate gene within LD block. We are sorry that this inappropriate displaying may have caused a misunderstanding of the accuracy of GWAS analysis. We agree with your opinion that red dashed lines should indicate the whole region of the LD block. We have corrected this inappropriate displaying for all the identified candidate genes from GWAS analysis in the revised Fig. 2.*

[8] For figure 6d Why there was no significance in SR weight between different types?

*Reply: Thank you for your comments. From Figure 6c, we can observe that allelic combinations of AAGG and AACG significantly enriched in cultivars, whereas other allelic combinations had low frequency. This resulted in no significance in SR weight among different allelic combinations.*

Reviewer #3:

1. The authors mentioned that the whole panel contained 38 landraces, but in Fig. 1a-c, I cannot find the 'landraces' subgroup. Did the author combine them with 'cultivars'? The authors should explain these accessions.

*Reply: Thank you very much for your comments. Your insights and suggestions are extremely important and reasonable, which helped us to improve the quality of the manuscript and avoid errors in our interpretation. Based on your valuable suggestions, we have made careful revisions on our manuscript. We hope that these revisions are satisfactory.*

*We marked the landraces in the variation map, phylogenetic and principal component analyses (Fig. 1a-1c). Although we tried to divide few subgroups based on phylogenetic and principal component analyses, there is no clear demarcation of geographic distribution patterns from cultivated cassava accessions and also no clear demarcation between landraces and cultivars (Fig. 1b, 1c). This is consistent with previous reports showing mixture of cultivars from different regions and between landraces and cultivars (Bredeson et al. 2016; Ramu et al. 2017). Because the extensive introduction and hybridization of cultivars may cause confusion in some cultivars, we assessed genetic distances and phylogenetic relationship among populations of wild progenitors and landraces (Fig. 1d, 1e and Table S4). The results indicate a close phylogenetic relationship between cassava populations from South America and Africa as well as between populations from Africa and Asia (Lines 130-141).*

*References:*

*Bredeson JV, Lyons JB, Prochnik SE, Wu GA, Ha CM, Edsinger-Gonzales E, Grimwood J, Schmutz J, Rabbi IY, Egesi C, et al. Sequencing wild and cultivated cassava and related species reveals extensive interspecific hybridization and genetic diversity. Nat Biotechnol. 2016, 34(5):562-70.*

*Ramu P, Esuma W, Kawuki R, Rabbi IY, Egesi C, Bredeson JV, Bart RS, Verma J, Buckler ES, Lu F. Cassava haplotype map highlights fixation of deleterious mutations during clonal propagation. Nat Genet. 2017, 49(6):959-63.*

2. In Fig. 2, 'epidermal type' seems like a qualitative trait (I am not sure); why authors found so many loci for this trait? Moreover, a total of 82 genes found in these LD blocks (Supp. Table 5), authors should explain the principle of candidate genes selection.

*Reply: We are sorry for the unclear description. The screening and identification of candidate genes from GWAS analysis are demonstrated as follows. First, the GWAS analyses were performed using different statistical models of GLM, MLM, CMLM, EMMAX and FAST-LMM. MTAs supported at least by two statistical models are defined as valid MTAs. Second, the genes located in 100kb windows which centered on the MTAs were considered as potential candidate genes for further analysis. Third, the potential candidate genes that have annotation and transcripts were selected for further analyzing whether they are in the LD block region and have allelic variation associated with phenotypic differences. We have included the principle of candidate genes selection in the methods section (Lines 485-498).*

*Epidermal type is generally divided into smooth and rough. However, the degree of rough type has difference in different accessions. Because the degree of rough type is difficult to accurately measure, we divide the phenotype of cassava accessions into rough and smooth types when performing GWAS analysis. We identified four significant signals on chr. 3, 5, 10, and 12, which covers 82 genes. Based on the above principle of candidate genes selection, we identified two candidate genes on chr 10 (Sc10g012040 and Sc10g012050) that encode auxin response factors and one candidate gene on chr 3 that encodes a mitochondrial carrier protein (Fig. 2a-2f). Auxin response factors and mitochondrial carrier protein was reported to widely participate in plant growth and development. Further detailed variation analysis indicated that the core allelic variations that caused non-synonymous mutation in these candidate genes have an effect on epidermal type (Fig. S5-S7).*

3. Line 191-203, I suggested authors creating a figure to show the overlapped genomic regions between the LD blocks identified by GWAS and highly heterozygous blocks, which could exhibit the significant effect of heterozygosity of trait-related loci better.

*Reply: It is a good suggestion. We observed 20 highly heterozygous blocks that overlapped with 6 GWAS signals (Table S11, Fig. S8). Moreover, cassava accessions carrying the highly heterozygous block on chromosome 1 showed higher frequency of red endothelium of SR than those carrying the corresponding homozygous block (Fig. S8i). These results further support the hypothesis that variation in heterozygosity is associated with key agronomic traits (Lines 208-214).*

4. Line 206-209 and Fig. 3, authors separated the whole panel (n = 388) into wild progenitors (n = 14) and cultivars (n = 336 or 374) to compare the genetic diversity, the population size of two subgroups is not equal. I suggest the authors randomly selecting 14 accessions from the cultivar subgroup to compare genetic diversity.

*Reply: We are sorry for the unclear description. Genomic diversity between heterozygous and homozygous regions was compared in 374 cultivated accessions using π and Tajima’s D values. FST value of heterozygous and homozygous regions was calculated between 14 wild progenitors and 374 cultivars in the original manuscript. In the revised manuscript, we used 14 wild progenitors and 14 randomly selected cultivars to assess the divergence of heterozygous and homozygous regions. The results showed that heterozygous regions have lower FST value than homozygous regions in most (14 out of 18) chromosomes between wild progenitors and cultivars (Fig. 3; Lines 216-226, 538-553).*

5. Line 217-218, '…we scanned genomic regions that exhibit drastic decreases in heterozygosity above the 99%…' according to the authors' assumption, heterozygosity of most traits-related loci should be positive (Line 50-51, Line 188-189, Line 177-179). Hence, the heterozygosity of human-selected regions should increase during domestication; why authors defined the genomic regions with a decrease in heterozygosity as domestication signals?

*Reply: Thank you for your comments. Heterozygosity is highly associated with nucleotide diversity. As known in various plant species, human selection resulted in decreased nucleotide diversity. In this study, we found 81 selective sweeps with decreased heterozygosity and nucleotide diversity, covering 6.5 Mb of the assembled genome, which approximately accounts for 56% of selective sweeps with reduction in nucleotide diversity (Fig. 3d; Lines 233-239). This indicates that decrease in nucleotide diversity by artificial selection is accompanied with reduction in heterozygosity during cassava domestication. In addition, all the identified eight candidate genes from GWAS analysis were not covered in the 138 selective sweeps that exhibit a decrease in nucleotide diversity during domestication (Fig. 2; Table S14), suggesting that these genes may not undergo selection. Clonal propagation is important to maintain crop heterozygosity. The heterozygous loci that showed desirable phenotype may be retained by clonal reproduction.*

6. I am a little confused about Fig. 4. Firstly, the selection signals (both for He and π) were not very high for SNP (11,848,921bp) within MeTIR1 (red dash line in Fig. 4d). I do not think this variation underwent strong selection. Perhaps some other variations (promoter or distant enhancer?) with greater differences of He and π signals between wilds and cultivars were the causal variations that impacted the expression level of MeTIR1, and further determined the SR starch content. Additionally, the genotypes of this SNP (GG, GT, and TT) are evenly distributed in wild accessions (Fig. 4g), but the SR starch content should be much lower in all wild accessions (Line 234-235), which indicated that this SNP obviously not associate with SR starch content. Why did the authors select this SNP? Also, The differences in SR starch content and SR weight were too small among genotypes. Although the authors performed further gene function experiments, it is still hard to judge that the two genes were genetically related to both traits. If authors could combine the GWAS results for SR starch content and SR weight with selection sweep results, I think the genotyping results could be better, and the conclusion could be more solid.

*Reply: This is a good suggestion. Based on your concerns, we realized that the previously identified variation in MeTIR1 is inaccurate. We genotyped all the SNP variations from the 9,919 bp total length in the upstream of MeTIR1. We found a SNP (chr2: 11,843,579 bp) in the upstream region (-5,067 bp) of MeTIR1, which might be associated with MeTIR1 expression and domestication (Fig. 4d, 4f). Compared with wild progenitors, cultivars showed a lower frequency of the heterozygous CG allele and a higher frequency of the homozygous GG allele (Fig. 4g). Starch content was significantly higher in accessions carrying the GG allele than in accessions carrying the CG allele (Fig. 4h). The effect of this allelic variation on SR starch content is more significant than the previous identified allelic variation. Accordingly, MeTIR1 exhibited significantly higher expression in accessions carrying the GG allele than in accessions carrying the CG allele (Fig. 4i) (Lines 259-266). Due to the limited total length of the upstream region of MeTIR1 (9,919 bp), the intensity of selection of this SNP is similar to that of the previous identified SNP (11,848,921 bp). Because MeAHL17 and MeTIR1 are located within the same selective sweep region and tightly linked, the strong selection of MeAHL17 possibly caused the selection of MeTIR1 by hitchhiking effect. This is also supported by analyzing combinations of allelic variation in MeTIR1 and MeAHL17 (Fig. 6; Lines 318-328).*

*As suggested, we hope that GWAS results for SR starch content and SR weight could be combined with selection sweep results. After careful checking, no significant allelic variation in heterozygosity within selection sweeps was associated with SR starch content and SR weight from GWAS analysis. GWAS is used to detect the correlation strength between allele variation and traits. Human selection not only resulted in the significant enrichment of GG allele in MeTIR1, but also caused the very low frequency of the CC allele in MeTIR1 (Fig. 4g,4h). Similarly, GG allele in MeAHL17 has very low frequency in cultivars (Fig. 4n,4o). This may have an effect on decreasing the sensitivity of GWAS. Additionally, SR starch content and SR weight are quantitative traits controlled by multiple genes. Thus, the GWAS peaks near the potential candidate genes MeTIR1 and MeAHL17 associated with SR starch content and SR weight respectively are below the threshold line.*

7. Minor concerns

[1] In Fig.1a, there are gaps on Chr. 2 and Chr. 9 for 'Wild' and 'All' populations for the SNP density distribution. Please check. Also, I suggest the authors adding the titles of axis Y (SNP density).

*Reply: We are sorry for our carelessness. We checked and redrew the SNP density distribution of wild progenitors, landraces and breeding lines. The axis Y (SNP density) was added to Fig. 1a.*

[2] How many SNPs were used for constructing phylogenetic tree and PCA analysis?

*Reply: Thank you for your kind suggestions. After filtering the low-quality SNPs, we obtained a subset of 1,344,463 high confidence SNPs for phylogenetic and PCA analyses, which was included in the revised manuscript (Lines 445, 454).*

[3] Line 491, why authors used 0.000001 as the P-value threshold for GWAS, is there any reference?

*Reply: Thank you for your suggestions. The threshold for GWAS was estimated as 0.05/total SNPs (log10(P) = -7.41). Because less effective signals were obtained above this threshold, we set the P-value threshold as 0.000001 according to previous studies (Fang et al. 2017; Ma et al. 2018) (Lines 492-494).*

*References:*

*Fang L, Wang Q, Hu Y, Jia Y, Chen J, Liu B, Zhang Z, Guan X, Chen S, Zhou B, Mei G, Sun J, Pan Z, He S, Xiao S, Shi W, Gong W, Liu J, Ma J, Cai C, Zhu X, Guo W, Du X, Zhang T. Genomic analyses in cotton identify signatures of selection and loci associated with fiber quality and yield traits. Nat Genet. 2017, 49(7):1089-1098.*

*Ma Z, He S, Wang X, Sun J, Zhang Y, Zhang G, Wu L, Li Z, Liu Z, Sun G, Yan Y, Jia Y, Yang J, Pan Z, Gu Q, Li X, Sun Z, Dai P, Liu Z, Gong W, Wu J, Wang M, Liu H, Feng K, Ke H, Wang J, Lan H, Wang G, Peng J, Wang N, Wang L, Pang B, Peng Z, Li R, Tian S, Du X. Resequencing a core collection of upland cotton identifies genomic variation and loci influencing fiber quality and yield. Nat Genet. 2018, 50(6):803-813.*

[4] Because this manuscript used multi-models and phenotypes under multi-environments, for all the local Manhattan plots in the main-text figures, if authors directly indicate what model (MLM, GLM …) and environment (2013 or 2016) these plots belong on the figure (not only in the captions), it would be easier to for readers.

*Reply: It is a good suggestion. The model and environment were added to Fig. 2, Fig. 5 and Fig. S3.*

[5] In Fig. 4, did the authors perform GWAS for 'storage root weight'? I did not find it in the Supplementary Figures.

*Reply: Thank you for pointing this out. We performed the GWAS analysis for storage root weight, but did not find any significant signal using the five statistical models, thus the manhattan plot was not included in Fig. S2.*

[6] Line 206-209, '…we split the cassava genome into heterozygous and homozygous regions according to heterozygous SNPs…', this sentence is too long and not very clear, did the authors mean to separate the population SNP-set into heterozygous and homozygous regions? Additionally, the FST value is the divergence level between two groups; I did not see which groups used to calculate the FST value here.

*Reply: We are sorry for the unclear description. We remapped Illumina short reads of SC205 to itself reference genome to identify heterozygous SNPs and define heterozygous and homozygous regions. We then compared the genomic diversity between heterozygous and homozygous regions in 374 cultivars. FST value was calculated between wild progenitors and cultivars. This information was included in the revised manuscript (Lines 538-553).*

[7] Line 486, does 'integrity > 0.8' mean 'missing < 20%'? If so, please use the same description as above (line 440).

*Reply: Thank you for your kind reminder. 'integrity > 0.8' means 'missing < 20%. We have used the same description 'integrity > 0.8' in the revised manuscript (Line 444).*

**Second round of review**

**Reviewer 1**

**The manuscript of Hu et al. describes the analysis of "Resequencing of 388 cassava accessions identifies valuable loci and selection for variation in heterozygosity". Although it has been revised according to the comments of reviewers, there are still some conclusions were not sufficient to prove. Comments are listed as below.**

**Major comments:**

**The author has responded to each of the questions raised, but there are still some questions remained to be explained. (1) The author explained there were only 15 wild progenitors released globally, so current studies on domestication of cassava can only be based on these wild progenitors, which cannot rule out the inaccuracy of domestication analysis due to the scarcity of wild materials. (2) Although the author mentions the multi-point difficulty for years, but the lack of multipoint data for many years, only with different GWAS analysis method to locate the candidate genes is not rigorous. Different statistical models used for GWAS analysis could not avoid this shortcoming. Whether there are known QTL regions of related traits that overlap with GWAS regions to demonstrate the reliability of GWAS results. Please add the third year phenotypic observation data of at least few traits to verify their reliability, this is really serious problem. (3) It is not enough only use Fst values to analyze the population divergence, it must include at least one other method. (4) Please also show the results of LD decay for different population.**

**Minor comments:**

**1. Manhattan plots should exhibit all 18 chromosomes, not only one.**

**2. Please add gene number in figure 2d.**

**3. Please mark which homozygous were presented by Hap1 and Hap3 in figure 2f and I or figure 2c and d, although it is represent in Figure S5-7.**

**4. L152-L194. These two paragraphs may be rearranged. It is not necessary to introduce all genes first, and then explain whether these genes have non synonymous mutations. Whether candidate genes of each trait have non synonymous mutations should be illustrate immediately after describing the alleles of haplotypes.**

**5. Figure S8i should illustrate allele frequency of each heterozygous.**

**6. L244. It is recommended to analyze other agronomic traits whose GWAS signal was overlapped with domestication sweeps.**

**7. L252. Please explain why not choose Sc09g001570 which highly expressed in both of the two wild progenitors.**

**8. L269. As the SNP is in the upstream region, overexpression and silencing of MeTIR1 gene in different haplotypes were not sufficient to prove this SNP is associated with starchy storage root of cassava.**

**9. Figure 4h-i. Please explain why the SR starch content was not different between haplotypes CC and GG, while the expression of MeTIR1 in these two haplotypes were significantly different. Taking into account this question, the number of accessions used for gene expression test is too small, please use at least 6 accessions per haplotype for expression test. Similar problem was also in figure 4o-p.**

**10. Figure 4d and 4j are the same. It is recommended to combine the two figures into one.**

**11. Figure 5c should add the number of each haplotype. And if there is GG haplotype, please also add CBB resistance of this haplotype.**

**12. Figure 5d-e. Four accessions were not sufficient to prove gene expression level and CBB resistance in the two haplotypes.**

**13. L310. Overexpression and silencing of MeAHL17 in two haplotypes were only able to prove MeAHL17 was positively associated with CBB resistance, but not able to prove "Selection for homozygous AA allele in MeAHL17 is associated with increased CBB susceptibility".**

**14. Figure 6d. The difference between each two allelic combinations were not significant. It is not sufficient to prove the sentence in L324 "Because the association between……of the large storage root."**

**Reviewer 2**

**I am satisfied with your revision.**

**Reviewer 3**

**The manuscript has been greatly improved, the authors have addressed all my concerns, I agree to accept this manuscript.**

**Authors Response**

**Point-by-point responses to the reviewers’ comments:**

Reviewer #1:

The manuscript of Hu et al. describes the analysis of "Resequencing of 388 cassava accessions identifies valuable loci and selection for variation in heterozygosity". Although it has been revised according to the comments of reviewers, there are still some conclusions were not sufficient to prove. Comments are listed as below.

*Reply: Thank you very much for your comments. Your comments and suggestions are insightful and reasonable, which helped us to improve the quality of the manuscript and correct errors in our interpretation. Based on your valuable suggestions, we have carefully revised our manuscript. We hope that these revisions are satisfactory.*

Major comments:

1. The author explained there were only 15 wild progenitors released globally, so current studies on domestication of cassava can only be based on these wild progenitors, which cannot rule out the inaccuracy of domestication analysis due to the scarcity of wild materials.

*Reply: We just obtained the resequencing data of six wild progenitors from another cassava research team, but these data are not released with permission. Although these data cannot be displayed formally in this manuscript, we are allowed to use these data to verify the reliability of our domestication analysis. We thus combined these data and our previous resequenced/collected data to perform domestication analysis. Resequencing of these six wild progenitors generated 85.6 Gb data with an average sequencing depth of 20× of the reference genome SC205 and identified 6,598,863 high-quality SNPs. We identified 90 selective sweeps (above the 99% threshold) that showed reduction in nucleotide diversity by comparing wild progenitors and cultivars, harboring 927 protein-coding genes. Of which, 77 selective sweeps overlapped with previously identified selective sweeps (Table Sa). In addition, we previously identified 16 growth- and development-associated genes that are differentially expressed in storage roots between wild progenitors and cultivars within selective sweep regions. These 16 genes, except for MOG1 (Sc11g009760) and GYL1 (Sc09g001570), were also covered in the newly identified 90 selective sweeps (Figure Sa). These results suggested that the domestication analysis results obtained by adding six wild progenitors are generally consistent with previous results, supporting the reliability of our domestication analysis.*

*We understand the reviewer’s concern. We added a statement in the discussion section: “more wild progenitors should be collected and re-sequenced to further verify the accuracy of our domestication analysis due to the limited number of wild progenitors used in this study”. We hope you can understand our difficulties to acquire more accessions of wild germplasm.*

*Please note: We uploaded Table Sa and Figure Sa into the submission system and named“Supporting information for Response to reviewer”.*

*Table Sa. Seventy-seven selective sweeps overlapped with previously identified selective sweeps.*

*Figure Sa. Selection signals for decrease in nucleotide diversity from wild cassava progenitors to cultivars. The horizontal blue line indicates values above the 99% thresholds for selection sweeps in nucleotide diversity. Red arrows indicate the positions of growth- and development-associated genes that are differentially expressed in storage roots between wild progenitors and cultivars within selective sweep regions with decreases in nucleotide diversity.*

2.Although the author mentions the multi-point difficulty for years, but the lack of multipoint data for many years, only with different GWAS analysis method to locate the candidate genes is not rigorous. Different statistical models used for GWAS analysis could not avoid this shortcoming. Whether there are known QTL regions of related traits that overlap with GWAS regions to demonstrate the reliability of GWAS results. Please add the third year phenotypic observation data of at least few traits to verify their reliability, this is really serious problem.

*Reply: We agree with your opinion. We collected eight important quantitative traits (including plant height, stem height, stem diameter, storage root number per plant, storage root weight, above-ground weight, cassava bacterial blight resistance and Resistant to T. cinnabarinus) in May 2021. The cassava plants were planted in May 2020 and were harvested in May 2021 at Danzhou, Hainan Province (109.5E, 19.5N). After careful calculation of these phenotypic data, we performed a GWAS analysis using different statistical models of GLM, MLM, CMLM, EMMAX and FAST-LMM. MTAs supported by at least two statistical models are defined as valid MTAs. Finally, we identified 15 additional MTAs for eight traits (Table S6; Figure S3). Combined with GWAS analysis for morphological traits during 2013 and 2016, we identified a total of 57 MTAs for 23 agronomically important traits (Table S6). By searching published articles related to QTL mapping of cassava traits, we found that four traits (storage root weight, storage root number per plant, storage root starch content, and storage root dry mass content) we studied were investigated by QTL mapping. We identified seven GWAS signals for these four traits. Of which, two GWAS signals for storage root weight and storage root number per plant overlapped with four known QTLs (Table S7) (Lines 151-157).*

3.It is not enough only use Fst values to analyze the population divergence, it must include at least one other method.

*Reply: We agree with your opinion. We further analyzed the population divergence using p-distance. On continent scale, the values of p-distance between cassava populations from South America and Africa (0.189) and between populations from Africa and Asia (1.017) were lower than those between populations from South America and Asia (0.200). On country scale, the values of p-distance between cassava populations from Brazil and Nigeria (0.174) and between populations from Nigeria and China (0.112) were lower than those between populations from Brazil and China (0.189). This is consistent with the results calculated by FST (Lines 133-141).*

4. Please also show the results of LD decay for different population.

*Reply: We agree with your opinion. Because of the difference of population size for different groups, we used 14 wild progenitors, 14 randomly selected landraces and 14 randomly selected breeding lines to perform LD decay analysis. The results showed that the LD extent in cassava was lower in the wild progenitors (7 kb; r2 = 0.2) than in the landraces (85 kb; r2 = 0.2) and breeding lines (177 kb; r2 = 0.2) (Figure S2) (Lines 129-131).*

Minor comments:

1. Manhattan plots should exhibit all 18 chromosomes, not only one.

*Reply: Thank you for your kind suggestion. Manhattan plots showing 18 chromosomes were presented in Figure S3. Because the amount of information in Figure 2 is too large, we are worried that the complete Manhattan plots would be difficult to be seen clearly in the main Figure 2.*

2. Please add gene number in figure 2d.

*Reply: Thank you for your kind reminder. Gene number was added in Figure 2d.*

3. Please mark which homozygous were presented by Hap1 and Hap3 in figure 2f and I or figure 2c and d, although it is represent in Figure S5-7.

*Reply: We agree with your opinion. The haplotype information in Sc10g012040 and Sc10g012050 as well as in the combinations of Sc10g012040 and Sc10g012050 was supplied in Figure 2f.*

4. L152-L194. These two paragraphs may be rearranged. It is not necessary to introduce all genes first, and then explain whether these genes have non synonymous mutations. Whether candidate genes of each trait have non synonymous mutations should be illustrate immediately after describing the alleles of haplotypes.

*Reply: It is a good suggestion. This section was revised according to your kind suggestion. The information of non synonymous mutations and their association with traits were illustrated immediately after describing candidate genes from GWAS analysis (Lines 151-218). This could improve the logic and readability of this part.*

5. Figure S8i should illustrate allele frequency of each heterozygous.

*Reply: Thank you for your suggestion. A total of 72 SNPs were detected in the block region of 6920000-6940000 on chromosome 1 that is potentially associated with SR endothelial color types. We identified 272 kinds of heterozygous haplotypes from 280 cassava accessions with the highly heterozygous block and trait values, suggesting extremely high frequencies of heterozygous genotypes of this region in the population. In addition, most of accessions carried the unique heterozygous alleles (at least one heterozygous SNP ) in this region, implying a high genetic diversity of this region. The frequency of heterozygous haplotypes were added in the legend of Figure S8i.*

6. L244. It is recommended to analyze other agronomic traits whose GWAS signal was overlapped with domestication sweeps.

*Reply: Thank you for your kind suggestions. Domestication of cassava has mainly resulted in increased yield and starch content of storage roots. We overlapped the GWAS signals for domestication associated traits, including SR diameter, SR weight, SR number per plant, SR dry mass content and SR starch content, with domestication sweeps and did not find overlaps. These domestication associated traits are quantitative traits and may be controlled by multiple genes with minor effect. We used a relatively strict GWAS pipeline that MTAs supported by at least two statistical models are defined as valid MTAs, which may cause some GWAS signals to be filtered out.*

7. L252. Please explain why not choose Sc09g001570 which highly expressed in both of the two wild progenitors.

*Reply: Thank you for your kind suggestions. Domestication of cassava has mainly resulted in increased yield and starch content of storage roots. Because this study focused on variation in heterozygosity, we must find allelic variation in heterozygosity of the candidate gene associated with increased yield or starch content of storage roots. Moreover, the frequency of allelic variation must show significant differences between wild progenitors and cultivars. We carefully checked the upstream and coding sequence region of Sc09g001570 and did not find allelic variation that meets the above two requirements.*

8. L269. As the SNP is in the upstream region, overexpression and silencing of MeTIR1 gene in different haplotypes were not sufficient to prove this SNP is associated with starchy storage root of cassava.

*Reply: We agree with your opinion. The sentence “These results indicate that selection for homozygous GG allele in MeTIR1 may be associated with the domestication of the starchy storage root of cassava” was removed from the revised manuscript.*

9. Figure 4h-i. Please explain why the SR starch content was not different between haplotypes CC and GG, while the expression of MeTIR1 in these two haplotypes were significantly different. Taking into account this question, the number of accessions used for gene expression test is too small, please use at least 6 accessions per haplotype for expression test. Similar problem was also in figure 4o-p.

*Reply: We agree with your opinion. We used six accessions per haplotype for expression test of MeTIR1. Due to the low frequency of GG allele in MeAHL17 (n=5), we used six (with AA allele), six (with AG allele) and five (with GG allele) accessions to perform expression test of MeAHL17. The results showed that the expression trends of MeTIR1 and MeAHL17 per haplotype are associated with phenotype variations (Figure 4i,o). The significant difference for phenotype variation of different haplotype is not completely in agreement with significant difference of gene expression among different haplotypes. The low frequency of the CC allele (n=7) in MeTIR1 and the GG allele (n=5) in MeAHL17 may affect statistical analysis of phenotypic differences of accessions carrying different haplotypes (Figure 4h,n).*

10. Figure 4d and 4j are the same. It is recommended to combine the two figures into one.

*Reply: We agree with your opinion. Figure 4d and 4j were combined as Figure 4d.*

11. Figure 5c should add the number of each haplotype. And if there is GG haplotype, please also add CBB resistance of this haplotype.

*Reply: We agree with your opinion. The number of each haplotype was added in Figure 5c. Due to the very low frequency of GG allele (5/322) (Fig. 4n), the accessions carrying GG allele was not covered in phenotyping of the cassava bacterial blight resistance.*

12. Figure 5d-e. Four accessions were not sufficient to prove gene expression level and CBB resistance in the two haplotypes.

*Reply: Thank you for your suggestion. We used six CBB-susceptible accessions carrying the AA allele and six CBB-resistant accessions with AG allele to detect expression level of MeAHL17 in the revised manuscript. The results showed that the transcripts of MeAHL17 in CBB-susceptible accessions were significantly lower than those in CBB-resistant accessions at both 0 dpi and 2 dpi (Figure 5d). For functional analysis of MeAHL17, CBB-resistant accessions were used as receptor materials for overexpression and silence of MeAHL17, and CBB-susceptible accessions were used as receptor materials for overexpression of MeAHL17. All these independent genetic transformation experiments support the positive function of MeAHL17 on CBB resistance.*

13. L310. Overexpression and silencing of MeAHL17 in two haplotypes were only able to prove MeAHL17 was positively associated with CBB resistance, but not able to prove “Selection for homozygous AA allele in MeAHL17 is associated with increased CBB susceptibility”.

*Reply: We agree with your opinion. The sentence “Selection for homozygous AA allele in MeAHL17 is associated with increased CBB susceptibility” was replaced by “These results suggest that MeAHL17 is positively associated with CBB resistance. ”in the revised manuscript (Lines 334-335).*

14. Figure 6d. The difference between each two allelic combinations were not significant. It is not sufficient to prove the sentence in L324 “Because the association between……of the large storage root.”

*Reply: We agree with your opinion. This part was revised as “the starch content and the frequency of resistance to CBB were not the most desirable in cultivars carrying the two allelic combinations” (Lines 343-344).*

**Third round of review**

**Reviewer 1**

**The authors should discuss more detailed about the GWAS results in different three years 2013, 2016 and 2020, is there some repeatable gene locus for different years for different agronomic traits? Especially for the two key genes the authors analyzed, MeTIR1 and MeAHL17, whether these two locus identified in 2020 year GWAS? Please add these discussion in the results part.**

**Authors Response**

**Point-by-point responses to the reviewers’ comments:**

Reviewer #1:

The authors should discuss more detailed about the GWAS results in different three years 2013, 2016 and 2020, is there some repeatable gene locus for different years for different agronomic traits? Especially for the two key genes the authors analyzed, MeTIR1 and MeAHL17, whether these two locus identified in 2020 year GWAS? Please add these discussion in the results part.

*Reply: Thank you very much for your kind suggestion. Your comments are insightful and reasonable, which helped us to improve the quality of the manuscript. We identified a total of 52 marker-trait associations (MTAs) for 23 agronomically important traits during 2013, 2016 and 2020. (Additional file 1: Table S6, Additional file 2: Fig. S3). Of which, seven traits were detected MTAs in at least two years, and two MTAs for stem height and storage root (SR) number per plant were repeatedly observed during 2016 and 2020 (Additional file 1: Table S7, Additional file 2: Fig. S4). This has been included into the results section of the revised manuscript.*

*We demonstrated that selection of MeTIR1 on chr 2 is associated with SR starch content and selection of MeAHL17 on chr 2 is associated with SR weight/cassava bacterial blight resistance (Fig. 4). In the phenotyping data of 2020, traits of SR weight and cassava bacterial blight resistance were measured (Additional file 1: Table S5). We identified three MTAs (chr2:15628399; chr8: 18229497; chr11: 17605354) for SR weight and three MTAs (chr13: 12894418; chr13: 13439650; chr13: 25673112) for cassava bacterial blight resistance from the data of 2020 (Additional file 1: Table S6). Although the MTA (chr2:15628399) for SR weight is close to the core variation (chr 2: 11671475) of MeAHL17, they still have a distance of 3.95Mb. Thus, it is difficult for us to determine that MeAHL17 is a candidate gene for the MTA. Human selection not only resulted in the significant enrichment of the GG allele of MeTIR1 and AA allele of MeAHL17, but also caused the very low frequency of the CC allele (n=7) of MeTIR1 and GG allele (n=5) of MeAHL17 (Fig. 4). This may have an effect on decreasing the sensitivity of GWAS.*
